# Pharmacological targeting of glutamatergic neurons within the brainstem for weight reduction

**DOI:** 10.1038/s42255-022-00677-8

**Published:** 2022-11-21

**Authors:** Marc Schneeberger, Nicola L. Brice, Kyle Pellegrino, Luca Parolari, Jordan T. Shaked, Keith J. Page, François Marchildon, Douglas W. Barrows, Thomas S. Carroll, Thomas Topilko, Victoria M. Mulligan, Robert Newman, Kevin Doyle, Roland Bürli, Daniel F. Barker, Angela Glen, María José Ortuño, Alexander R. Nectow, Nicolas Renier, Paul Cohen, Mark Carlton, Nathaniel Heintz, Jeffrey M. Friedman

**Affiliations:** 1 Laboratory of Molecular Genetics, Howard Hughes Medical Institute, The Rockefeller University, New York, NY USA; 2 Laboratory of Neurovascular Control of Homeostasis, Department of Cellular and Molecular Physiology, Yale School of Medicine, New Haven, CT USA; 3 Wu Tsai Institute for Brain and Cognition, Yale School of Medicine, New Haven, CT USA; 4 Cerevance, Cambridge, UK; 5 Laboratory of Molecular Metabolism, The Rockefeller University, New York, NY USA; 6 Bioinformatics Resource Center, The Rockefeller University, New York, NY USA; 7 Sorbonne Université, Paris Brain Institute, INSERM, CNRS, Hopital de la Pitié Salpétière, Paris, France; 8 Takeda Cambridge, Cambridge, UK; 9 Department of Genetics, Yale School of Medicine, New Haven, CT USA; 10 College of Physicians and Surgeons, Columbia University, New York, NY USA; 11 Laboratory of Molecular Biology, Howard Hughes Medical Institute, The Rockefeller University, New York, NY USA

**Keywords:** Pharmacogenetics, Neural circuits, Transcriptomics, Obesity

## Abstract

Food intake and body weight are tightly regulated by neurons within specific brain regions, including the brainstem, where acute activation of dorsal raphe nucleus (DRN) glutamatergic neurons expressing the glutamate transporter Vglut3 (DRN^Vglut3^) drive a robust suppression of food intake and enhance locomotion. Activating Vglut3 neurons in DRN suppresses food intake and increases locomotion, suggesting that modulating the activity of these neurons might alter body weight. Here, we show that DRN^Vglut3^ neurons project to the lateral hypothalamus (LHA), a canonical feeding center that also reduces food intake. Moreover, chronic DRN^Vglut3^ activation reduces weight in both leptin-deficient (ob/ob) and leptin-resistant diet-induced obese (DIO) male mice. Molecular profiling revealed that the orexin 1 receptor (Hcrtr1) is highly enriched in DRN Vglut3 neurons, with limited expression elsewhere in the brain. Finally, an orally bioavailable, highly selective Hcrtr1 antagonist (CVN45502) significantly reduces feeding and body weight in DIO. Hcrtr1 is also co-expressed with Vglut3 in the human DRN, suggesting that there might be a similar effect in human. These results identify a potential therapy for obesity by targeting DRN^Vglut3^ neurons while also establishing a general strategy for developing drugs for central nervous system disorders.

## Abstract (web summary)

Schneeberger et al. show that glutamatergic neurons within the dorsal raphe nucleus of the brainstem are enriched with the orexin 1 receptor Hcrtr1, which can be pharmacologically targeted to treat obesity in mice.

## Main

Obesity is an important medical problem associated with a set of co-morbidities that includes heart disease, diabetes and hypertension^1,2^. Weight loss ameliorates these associated diseases, but diets have limited efficacy for maintaining weight loss for most individuals^2^. Bariatric surgery has proven to be effective for maintaining long-term weight loss, as have a series of injectable agents derived from GLP-1 and other gastrointestinal peptides, which also show great promise^2–4^. However, highly efficacious oral agents for the treatment of obesity are limited. Compliance is generally superior for oral medicines versus injectable agents, and the development of orally bioavailable medicines for treating obesity would add an important option. Food intake and body weight are regulated by specific neurons in a number of brain regions, and we thus set out to use this information to identify drugs that modulate the activity of specific neural populations that regulate weight^5^.

In recent studies, we performed an unbiased screen for neurons that are activated by food restriction and identified two subsets of neurons in the DRN that regulate food intake and body weight^6,7^. In those studies, we found that a GABAergic subpopulation is activated by fasting and increases food intake, while a glutamatergic (Vglut3) subpopulation is activated by refeeding and reduces food intake (DRN^Vglut3^)^6^. The DRN, located in the brainstem just below the cerebral aqueduct, is a key hub in the central nervous system that is responsible for coordinating numerous physiologic and behavioral functions^8^. These functions include, but are not limited to, regulation of emotional state, mediating social interactions and conveying pain^9–11^. One key characteristic of the DRN is that it contains the largest pool of serotonergic neurons in the brain (one-third of the total in the entire brain). Serotonin (5-HT) regulates feeding, and modulators of 5-HT signaling have been tested extensively using non-selective pharmacologic agents^12^, and have shown some efficacy in inducing weight loss. We also found that DRN^Vglut3^ neurons express 5-HT, raising the possibility that drugs that increase the activity of these neurons might also induce weight loss^13^. We tested this and found that modulation of the activity of these neurons using chemogenetics reduces weight in obese leptin-deficient ob/ob and in leptin-resistant DIO mice. We next sought to replicate this effect pharmacologically by searching for drug targets that are enriched in DRN^Vglut3^ neurons. We performed molecular profiling and found that DRN^Vglut3^ neurons express high levels of the Hcrt1 receptor, which is only sparsely expressed outside the DRN. Finally, we show that an orally bioavailable, highly selective Hcrtr1 antagonist reduces food intake and body weight in ob/ob and DIO mice. These findings have potential clinical implications and also establish a general approach for identifying drugs that modulate neural activity as potential treatments for central nervous system disorders.

## Results

### DRN^Vglut3^ neurons integrate into the canonical feeding circuit

We previously found that DRN^Vglut3^ neurons reduce food intake in both lean and obese mice^6^. We also found that the arcuate (ARC) nucleus, a key site of leptin action and feeding control, sends dense projections to the DRN^14–19^, raising the possibility that DRN^Vglut3^ neurons are downstream of leptin-responsive neurons in the ARC^20^. Obesity is generally associated with leptin resistance, and if DRN^Vglut3^ neurons are functionally downstream, it would suggest that modulating their activity could reduce the weight of leptin-resistant obese animals. To test this, we performed a series of anatomic and functional studies to identify functional outputs of DRN^Vglut3^ neurons and delineate neural mechanism(s) by which they control food intake.

**Fig. 1:**
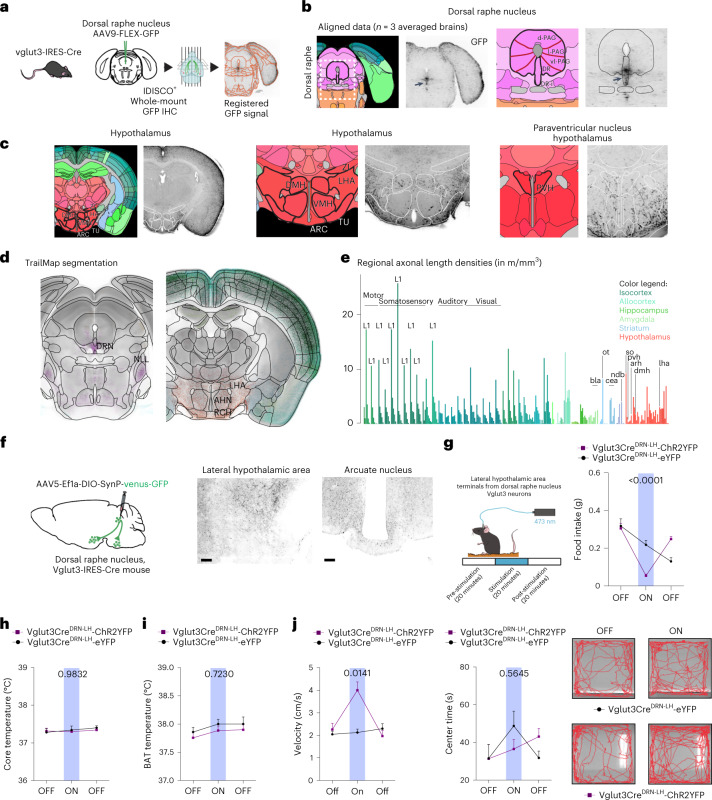
DRN^Vglut3^ neurons are integrated to the broader feeding circuitry through ascending projections. **a**, Schematic of whole-brain projection mapping of DRN^Vglut3^ neurons (IDISCO^+^/ClearMap). **b**, Allen Brain Atlas annotation and localization of the Vglut3 cell bodies expressing GFP in the DRN (*n* = 3 mice). **c**, Axonal projections from DRN^Vglut3^ neurons into numerous hypothalamic loci, such as dorsomedial hypothalamus (DMH), lateral hypothalamic area (LHA), arcuate nucleus (ARC), ventromedial hypothalamus (VMH) and paraventricular hypothalamus (PVH), following the Allen Brain Atlas annotation. **d**,**e**, Quantitative projection mapping of hypothalamic projections using TRAILMAP modified code. **f**, Left, Schematic of axonal nerve endings in the LHA, created using a synaptophysin–GFP adeno-associated virus (AAV9-DIO-Synp-Venus-GFP). Right, representative IHC validation of DRN^Vglut3^ projections to ARC and LHA. **g**, Left, Schematic of DRN^Vglut3^ terminal stimulation in the LHA to assess energy balance. Right, optogenetic (AAV5-DIO-ChR2-eYFP or AAV5-DIO-eYFP (control)) terminal stimulation from DRN^Vglut3^ to the LHA. Mice expressing ChR2 exhibited suppressed food intake when ChR2 was stimulated in the laser-on phase of a test in which mice were subjected to laser stimulation (*n* = 5–6 mice per group) (degrees of freedom (2), *F* statistics 20.31, *P* < 0.001) **h**,**i**, Activation of DRN^Vglut3^ terminals in the LHA does not impair thermoregulation, assessed as core temperature (**h**) or BAT thermogenesis (**i**) (*n* = 5-6 mice per group). **j**, Left, optogenetic photoactivation of DRN^Vglut3^ neuron terminals to the LHA enhances acute locomotion (2 degrees of freedom, *F* statistic 10.98) in an OFT. Middle, no differences are observed in the transitions or time spent in the center (measure of anxiety) of the OFT. Right, representative locomotor activity traces for optogenetic activation of DRN^Vglut3^ terminals in the LHA. Traces were taken from a stimulation (laser-on) epoch. Data are represented as mean ± s.e.m. *P* values were calculated using a two-way analysis of variance (ANOVA) with a multiple-comparisons test (Tukey post-hoc). In **a**–**e**, the number of projections of each sample in the considered regions or annotated brain areas was analyzed using independent two-sample Student’s *t*-test, assuming unequal variances, using ClearMap/TrailMap. Multiple-comparison corrections were applied to *P* values to obtain *q* values (for false-discovery rate). In **g**–**j**, a two-way repeated-measures ANOVA was used, comparing control and treated groups (*n* = 5 mice per group). The blue region in **g**–**j** highlights the laser-on epoch. Scale bars, 200 µm. *P* < 0.05 is considered significant and are indicated above the bar graphs. Brain regions indicated in Fig. 1e are as follows: bla (basolateral amygdala), cea (central amygdala), pvh (paraventricular hypothalamus), arh (arcuate nucleus), dmh (dorsomedial hypothalamus), lha (lateral hypothalamic area), ndb (nucleus of the diagonal band), so (supraoptic nucleus) and ot (optic tract). Source data

**Extended Data Fig. 1:**
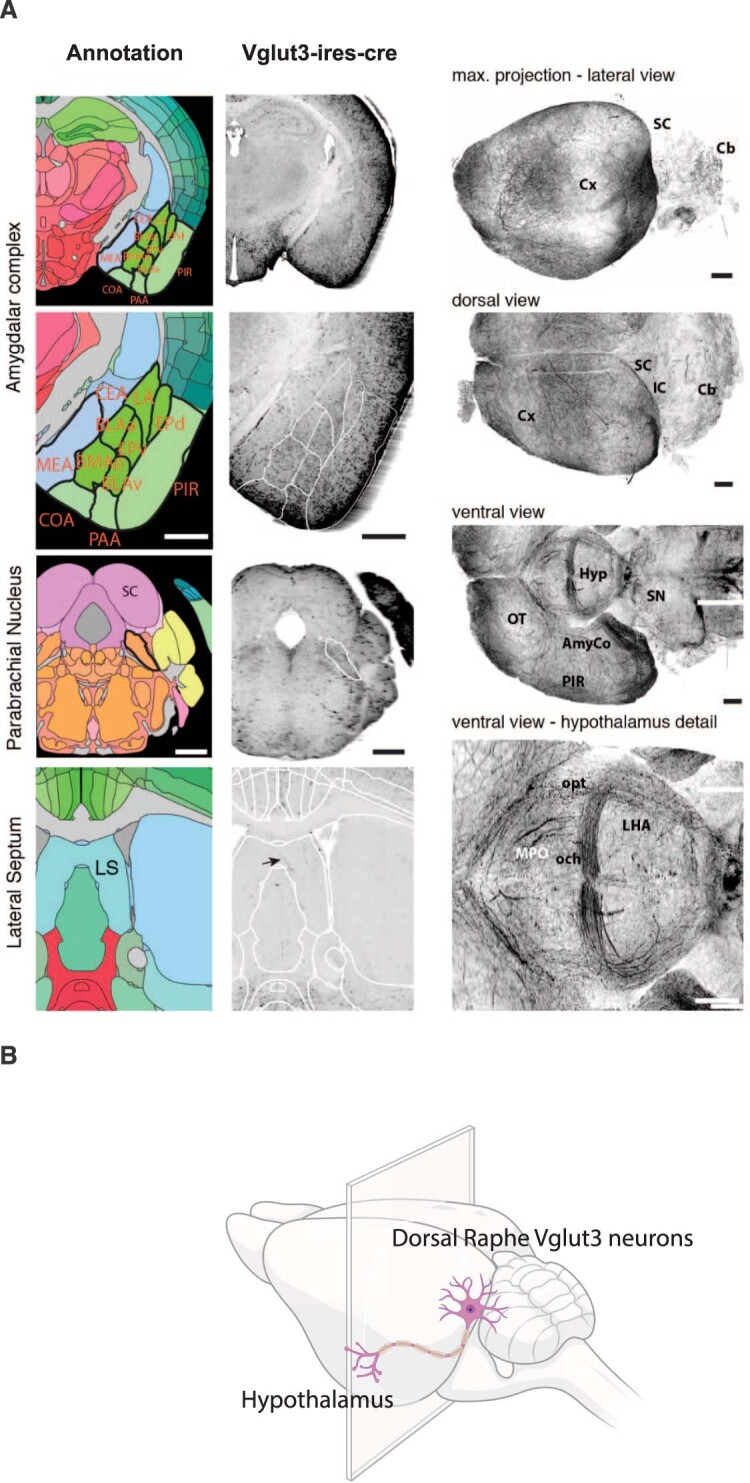
Ascending projections from DRN^Vglut3^ Neurons Demonstrate Integration to Brain Loci with Previously Reported Roles in Energy Balance Control. **(a)** Whole mount axonal projections using clearing/immunostaining technology IDISCO+ from DRN^Vglut3^ neurons to multiple extrahypothalamic loci with previously reported roles in energy homeostasis regulation. In particular, we highlight projections to the Lateral Septum and Amygdala (basolateral and central section) with known feeding regulation roles and the Parabrachial Nucleus with known implications in both feeding and thermoregulation. **(Left Panels)** Allen brain atlas annotations of the aforementioned regions. **(Middle Panels)** Coronal reslices of axonal projections to the aforementioned regions. **(Right panels)** Whole-brain, 3D reconstruction images of DRN^Vglut3^ projections throughout the brain from multiple viewpoints. Scale bars 200um. **(b)** Schema of the most prominent site of projection from DRN^Vglut3^ neurons to the LHA. See also Extended Data Video 1 for animated view of projection patterns from DRN^Vglut3^ neurons and Supplementary Table 1 for quantification of the projection targets identified in the entire brain.

To assess the anatomical relationship between DRN^Vglut3^ neurons, the ARC and other brain regions, we first mapped the axonal projections of DRN^Vglut3^ neurons by injecting an adeno-associated virus (AAV) expressing an axon-filling green fluorescence protein (GFP) (AAV9-FLEX-GFP) into the DRN of Vglut3-IRES-Cre mice, followed by whole mount clearing and mapping of GFP using the iDISCO^+^ and CLEARMAP pipeline tandem^21,22^ (Fig. 1a–c). After 6 weeks (the length of time needed for GFP expression in axons and terminals), brains were cleared, and the intact axonal architecture of DRN^Vglut3^ neurons was imaged using light sheet microscopy (Supplementary Video 1 and Supplementary Table 1). After three-dimensional (3D) mapping, DRN^Vglut3^ projections were annotated using the Allen Brain Atlas^23^. Axons traced from the soma of DRN^Vglut3^ cell bodies were localized in a dense cluster along the midline of the DRN and also projected broadly to a number of other anatomic sites (Fig. 1a–c and Extended Data Fig. 1). We detected negligible projections to the aforementioned ARC, a key site of leptin action, as well as the ventromedial hypothalamus (VMH) (Fig. 1c). This finding is consistent with the possibility that DRN^Vglut3^ neurons are downstream of leptin signaling and thus a potential cellular target for treating leptin-resistant forms of obesity. We next mapped the functional outputs of these neurons.

We found dense projections to the LHA and the dorsomedial hypothalamus (DMH), with sparser projections to the paraventricular hypothalamus (PVH). Projections from DRN^Vglut3^ neurons to the parabrachial nucleus (PBN) neurons, the amygdalar complex and the lateral septum (LS), two subcortical regions controlling feeding, were also observed (Extended Data Fig. 1a)^18,19,24,25^. In contrast to DRN^Vgat^ neurons, which do not project to cortical structures^3^, DRN^Vglut3^ neurons send widespread projections throughout different cortical layers, mostly arborizing in layer 1 of the somatosensory cortex and medial prefrontal cortex (Extended Data Fig. 1a). Together, these data show that DRN^Vglut3^ neurons send extensive ascending long-range projections to the hypothalamus and brainstem, including several canonical feeding centers, and broadly throughout the brain, but not to the ARC.

To provide a quantitative analysis of the volumetric image generated from our whole mount data of these projections, we used TrailMap^26^ (Fig. 1d), software that allows region-based quantification matched to the Allen Brain Atlas, thereby reducing imaging artifacts and false breaks in axonal projections. As a result, it generates an output data set of the preferentially innervated areas by a specific labeled neuronal type. Data generated using TrailMap confirmed prominent axonal projections from DRN^Vglut3^ neurons to the LHA (Fig. 1e and Supplementary Table 1), a canonical feeding center^27^. Quantitation of these data revealed dense projections to the tuberal area (TU), supraoptic nucleus (SO), lateral preoptic area (LPO), LHA and VMH, with negligible projections to the ARC and PVH (Fig. 1d,e and Supplementary Table 1). The well-established role of the LHA in controlling feeding^28^ led us to study the function of this projection.

**Extended Data Fig. 2:**
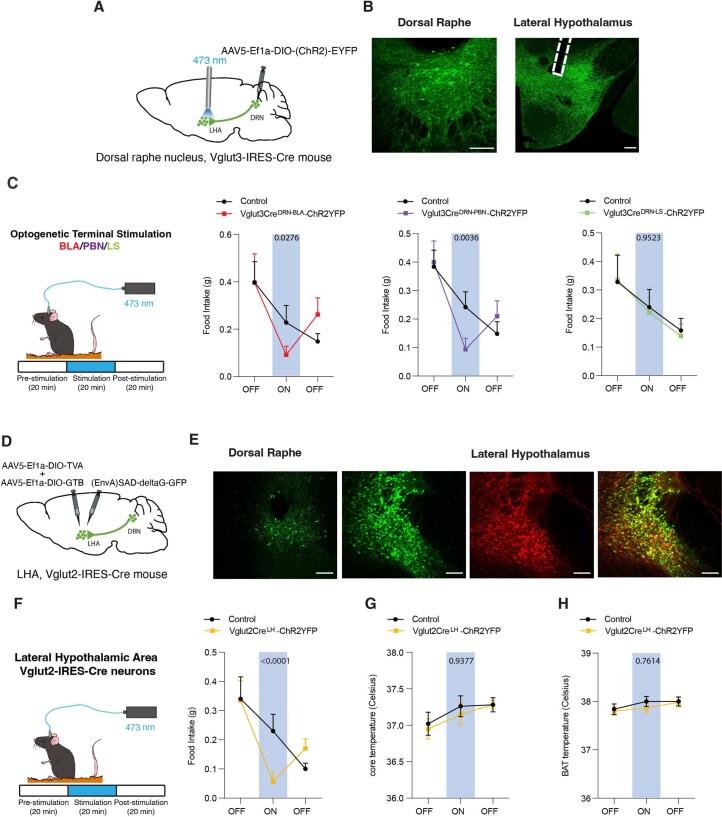
DRN^Vglut3^ Neurons Anatomic Analysis Suggests the Blockade of Energy Homeostasis occurs through LHA^Vglut2^ neurons. . (**a**) Schema for DRN^Vglut3^ terminals in the LHA, using and AAV5-DIO-ChR2-eYFP injected into the DRN and an optic fiber placed in the LHA. (**b**) Representative images of cell bodies of 1 mouse from a samples size of 3 independent biological samples. (Left panel) injected with AAV5-DIO-ChR2-eYFP in the DRN and axonal terminals (Right panel) in the LHA. Dashed line shows optic fiber location in the LHA. (C,Left) Schema representing terminal activation DRN^Vglut3^ terminals in the BLA (red), PBN (purple) and LS (green). (**c**, Right) Stimulation of terminals in the LHA (Laser On phase) significantly suppresses food intake (treatment: p < 0.001) in the BLA and PBN but not in the LS (n = 5 control groups, 5 group BLA, 6 group PBN, 7 group LS). (**d**) Schema illustrating monosynaptic retrograde tracing from Vglut2 neurons in the LHA to the DRN (n = 3 independent biological samples). (**e**, Left) Retrogradely labeled (green) neurons were found in the DRN following the monosynaptic tracing experiment (**e**, Right) Dual labelling for both mCherry and GFP is observed in the LHA following the monosynaptic tracing experiment, demonstrating both helper virus and rabies virus expression. (F-Left) Schema representing the strategy used to optogentically activate LHA^Vglut2^ cell bodies. (**f-**Right) Stimulation of Vglut2 neurons in the LHA (Laser On phase) significantly suppresses food intake (treatment: p < 0.001) (n = 5 control group, 6 group LHA). (G-H) Activation of LHA^Vglut2^ cell bodies does not impair thermoregulation as observed by measurement of BAT temperature using IPTT-300 transponders (**g**) or core body temperature through anal thermometer measurements. (**h**) (n = 5 control group, 6 group LHA). Data are presented as mean ± SEM. Two-way repeated measures ANOVA comparing control and treated groups (n = 5-7 mice per group) followed by an ad hoc Sidak’s multiple comparison test (C, F, G, H). Blue-shaded region highlights Laser On epoch. Scale bars, 200µm. p < 0.05 is considered significant. LHA (Lateral Hypothalamic Area), DRN (Dorsal Raphe Nucleus), BLA (Basolateral Amygdala), PBN (Parabrachial Nucleus), LS (Lateral Septum) and BAT (Brown adipose tissue). Source data

We next sought to identify whether the cellular targets of DRN^Vglut3^ neurons in the LHA contribute to the reduction of food intake driven by DRN^Vglut3^ neurons. To do so, we identified synapses from DRN^Vglut3^ neurons in the LHA after injection of an AAV expressing synaptophysin-venus-GFP (AAV5-DIO-SynP-Venus-GFP) into the DRN of Vglut3-IRES-Cre mice. Synaptophysin-venus-GFP preferentially localizes in nerve terminals and labels sites of synapse formation^29^. Six weeks after the AAV injection, we imaged the fluorescently labeled terminals and found synapses onto a subset of neurons in the LHA, recapitulating the dense projections to this nucleus (Fig. 1f). To demonstrate that this prominent projection (DRN^Vglut3^→LHA) is a functional projection, we injected AAV5-EF1a-DIO-ChR2-EYFP into the DRN of Vglut3-IRES-Cre mice, followed by implantation of an optical fiber above the LHA^7^ (Extended Data Fig. 2a). After 6 weeks (the time needed for animals to recover from the surgery and to express ChR2-eYFP in LHA terminals (Extended Data Fig. 2b)), we tested the effect of activating these projections by measuring food intake after an overnight fast in three 20-minute epochs (pre-stimulation, stimulation and post-stimulation) using 10-Hz laser stimulation (Fig. 1g, left). Optogenetic activation of DRN^Vglut3^ projections to the LHA led to a rapid and significant suppression of food intake averaged between the laser-on and laser-off epochs (Fig. 1g, right) (food intake in laser-on phase (20 minutes): controls, 0.218 g; Vglut3-DIO-ChR2-EYFP ,0.056 g; *P* < 0.0001). There was no effect on brown adipose temperature (BAT) or core body temperature after photoactivation (Fig. 1h,i). These results demonstrate that DRN^Vglut3^ neurons send functional projections to the LHA to reduce food intake.

We previously reported that DRN^Vglut3^ neurons control both food intake and locomotor activity^6^. Hence, we next tested whether this prominent projection target of DRN^Vglut3^ neurons to the LHA is also capable of modulating locomotor activity in an open field test (OFT). ChR2-EYFP was introduced into DRN^Vglut3^ neurons, and their terminals in the LHA were photostimulated in three 5-minute epochs (pre-stimulation, stimulation and post-stimulation) using 10-Hz laser stimulation. Activation of the terminals resulted in an increase of locomotor activity in the laser-on epoch (Fig. 1j, left), without changes in OFT center time (reflecting anxiety-like behavior) (Fig. 1j, middle). Of note, we also observed a reduction in feeding after activating DRN^Vglut3^ neurons terminals in the basolateral amygdala (BLA)^30^ and the PBN^31^, whereas activating the projections to the LS did not lead to reduced feeding (Extended Data Fig. 2c). The BLA and PBN are also well-established feeding centers, and these data reinforce the conclusion that DRN^Vglut3^ neurons are integrated in an expanded neural network that controls feeding^30,31^.

Previous studies have established a role for glutamatergic Slc17a6 (Vglut2) LHA neurons as rapid suppressors of feeding^32^, and we hypothesized that these are DRN^Vglut3^ cellular targets in the LHA. We investigated this using transneuronal monosynaptic rabies mapping^33^. AAVs expressing protein G (AAV5-Ef1A-DIO-GTB) and the avian receptor (TVA) (AAV5-Ef1A-DIO-TVA-mCherry) were first transduced into LHA^Vglut2^ neurons of Vglut2-Cre mice. Two weeks after injection (the time required for helper rabies virus to express TVA and protein G in Vglut2-expressing neurons), a protein G-deficient and protein EnvA-expressing rabies virus (EnvA-SAD-Rb-ΔG-GFP) were injected into the LHA (Extended Data Fig. 2d). As expected, we observed both mCherry and GFP expression in the LHA (Extended Data Fig. 2e). However, we also observed extensive labeling of the ventral region of the DRN (Extended Data Fig. 2e), where Vglut3 neurons are localized. These data raise the possibility that DRN^Vglut3^ neurons regulate feeding by activating Vglut2 neurons in the LHA. To test this, we injected an AAV expressing the excitatory opsin channelrhodopsin (AAV5-EF1a-DIO-ChR2-EYFP) into the LHA of Vglut2-Cre mice. Optogenetic activation of Vglut2 neurons in the LHA led to rapid and significant suppression of food intake between the laser-on and laser-off epochs after an overnight fast (Extended Data Fig. 2f, right) (food intake in laser-on phase (20 minutes): controls, 0.230 g; Vglut3-DIO-ChR2-EYFP, 0.052; *P* < 0.0001). Here again, there was no effect on BAT or core body temperature after photoactivation (Extended Data Fig. 2g,h).

Together, these data demonstrate that activating terminals from DRN^Vglut3^ neurons to projections in the LHA replicates the previously reported effect on feeding and locomotor activity seen after activating the soma. Importantly, some of the targets, including the LS (Extended Data Fig. 2c) and the central amygdala, do not show a response, and others show lesser responses (BLA, PBN), suggesting minimal or no collateralization of DRN^Vglut3^ axons. These data suggest that these neurons regulate energy balance, not only through a local DRN circuit^2^, but also through ascending projections to the LHA and elsewhere. Moreover, these data suggest that DRN^Vglut3^ neurons mediate its effects by activation of Vglut2 neurons in this region. These anatomic data integrate DRN^Vglut3^ neurons into canonical feeding circuitry downstream of sites of leptin signaling in the ARC, raising the possibility that chronic modulation of those neurons in leptin-deficient or leptin-resistant obese mice might show efficacy for reducing weight^34,35^.

### DRN^Vglut3^ neurons reduce weight in ob/ob and DIO mice

**Fig. 2:**
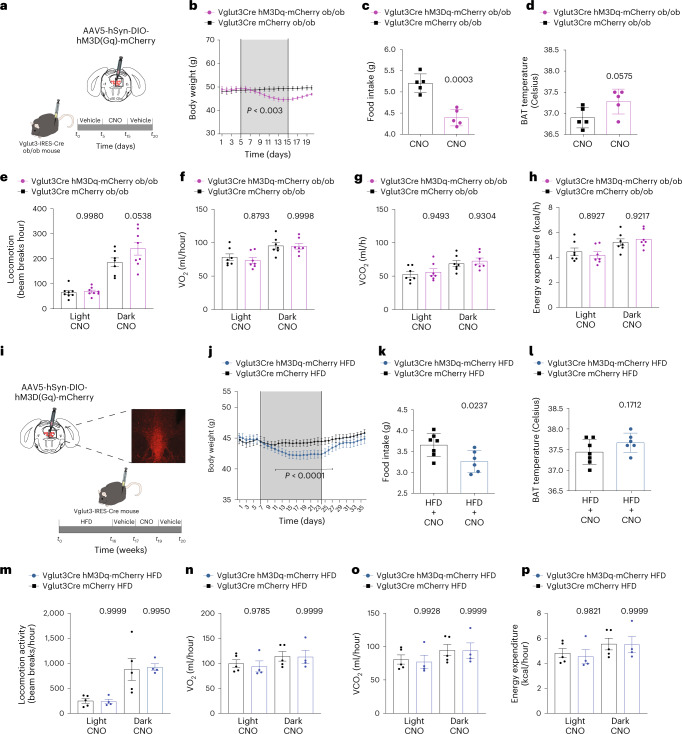
Chronic neuromodulation of DRN^Vglut3^ neurons can reduce body weight in obese mice (DIO and ob/ob). **a**, Schematic of the curative approach using DREADDs in Vglut-Cre ob/ob mice injected with the hM3(Gq) DREADD into the DRN (Vglut3-Cre^ob/ob-hM3(Gq)^ mice; *n* = 5). **b**, Weight curve after stimulating DRN^Vglut3^ neurons in ob/ob mice (*P* = 0.003). **c**, Food intake after chronic stimulation of Vglut3-Cre^ob/ob-hM3(Gq)^ mice. **d**, Adaptive thermogenesis in DRN-Vglut3-Creo^ob/ob-hM3(Gq)^ mice. In **a**–**c**, littermate DRN-Vglut3-Cre^ob/ob-mCherry^ mice were used as controls. **e**–**h**, Indirect calorimetry in DRN-Vglut3-Creo^ob/ob-hM3(Gq)^ or DRN-Vglut3-Cre^ob/ob-mCherry^ mice using metabolic cages. The parameters measured in a day–night cycle were locomotor activity (**e**) (*n* = 7–8 mice per group), oxygen consumption (**f**) (*n* = 7–8 per group), CO_2_ production (**g**) (*n* = 7–8 per group) and energy expenditure (**h**) (*n* = 7–8 per group). **i**, Schematic of the curative approach using DREADDs in DRN-Vglut3-Cre^HM3Dq^ mice with DIO (DRN-Vglut3-Cre^DIO-^^HM3Dq^ mice). The representative image shows the injection site. **j**, Body weight of mice with a DIO background after stimulation DRN^Vglut3^ neurons (*n* = 6–7 per group, *P* < 0.0001). **k**, Food intake during the chronic stimulation of DRN^Vglut3^ neurons, demonstrating hypophagia (*n* = 6–7 per group). **l**, Adaptive thermogenesis in DRN-Vglut3-Cre^DIO-HM3Dq^ mice. In **i**–**l**, littermate Vglut3-Cre^+^ DIO mice injected with AAV2/5-DIO-mCherry were used as controls. All mice received saline for 7 days, CNO for 14 days and saline for 7 days in a sequential manner. **m**–**p**, Indirect calorimetry assessment in DRN-Vglut3-Cre^DIO-HM3Dq^ and DRN-Vglut3-Cre^DIO-mcherry^ mice. The parameters measured in a day–night cycle were locomotor activity (**m**) (*n* = 4–5 per group), oxygen consumption (**n**) (*n* = 4–5 per group), CO_2_ production (**o**) (*n* = 4–5 per group) and energy expenditure (**p**) (*n* = 4–5 per group). Data are represented as mean ± s.e.m. *P* values were calculated using a two-way ANOVA with a multiple-comparisons test using a Tukey post-hoc approach (**b**,**j**) or an unpaired two-tailed Student’s *t*-test (**c**,**d**,**k**,**l**), or using CalR software and a two-sided ANCOVA regression analysis taking body weight into account (**e**–**h**,**m**–**p**). *P* < 0.05 is considered significant. The CNO dose used was 1 mg/kg. Source data

Although acute activation of DRN^Vglut3^ neurons has been shown to reduce food intake, the effect of chronic activation on the weight of obese mice has not been assessed^1^. We first tested this by mating Vglut3-IRES-Cre mice to ob/ob mice, and offspring were subjected to stereotactic injections of a Cre-dependent AAV expressing a muscarinic excitatory designer receptor exclusively activated by a designer drug (DREADD)^36^ called hM3D(Gq) (AAV5-hSyn-DIO-hM3D(Gq)-mCherry) into the DRN. Vglut3-Cre ob/ob mice injected with AAV-DIO-mCherry were used as littermate controls. After 2 weeks, the amount of time required for adequate gene expression in the DRN, the DREADD-specific ligand clozapine-*N*-oxide (CNO) or saline was injected into transgenic mice (Fig. 2a, left). CNO diminished food intake in the Vglut3-hm3D(Gq) ob/ob mice over the duration of the 2-week study (Fig. 2b) and significantly decreased body weight (Fig. 2a, right), with the treated animals losing 5% of their weight relative to controls (*P* = 0.003). Mice rapidly regained weight when the CNO treatment was stopped (Fig. 2a, right). We also monitored BAT temperature after DRN^Vglut3^ activation but, in contrast to DRN^Vgat^ neurons^7^, CNO failed to induce a significant change in thermogenesis in these animals (Fig. 2c).

We next tested whether activation of DRN^Vglut3^ neurons would alter systemic energy expenditure. To do so, a separate cohort of Vglut3-IRES-Cre ob/ob mice was injected with AAV5-hSyn-DIO-hM3D(Gq)-mCherry or AAV5-hSyn-DIO-mCherry, and mice were then placed in metabolic cages for indirect calorimetry. Activating DRN^Vglut3^ neurons via CNO injection provoked a significant decrease in food intake in animals expressing the DREADD receptor, with no effect of CNO upon locomotor activity (Fig. 2e), oxygen consumption (VO_2_) (Fig. 2f), carbon dioxide production (VCO_2_) (Fig. 2g) or total energy expenditure (Fig. 2h). Regression analysis showed a highly significant effect (*P* < 0.001) considering the body weight of each mouse, using the CalR platform and analysis of covariance (ANCOVA) statistical analyses^37^.

These results demonstrate that chronic activation of DRN^Vglut3^ neurons can reduce the food intake and body weight of leptin-deficient ob/ob mice without altering energy expenditure. Our finding that ob/ob mice have reduced food intake and body weight after DRN^Vglut3^ activation suggests that these neurons can reduce weight independently of leptin. We next tested the effect of activating DRN^Vglut3^ neurons in leptin-resistant DIO mice that have high circulating levels and a diminished response to exogenous hormone^38^ (Fig. 1).

**Extended Data Fig. 3:**
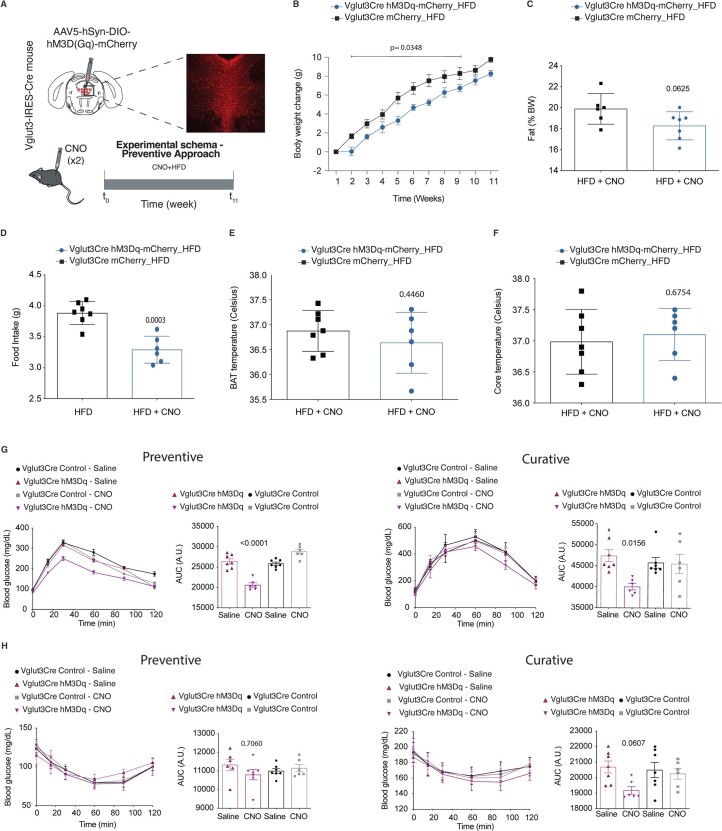
Chemogenetic Stimulation of DRN^Vglut3^Neurons fed HFD Exhibits a Significant Reduction of Energy Balance in a Preventive Setting. . (**a**) Schematic for chronic stimulation of DRN^Vglut3^ neurons in HFD fed mice injected with an AAV5-DIO-hm3Dq-mcherry in DRN^Vglut3^ neurons using the DREADD agonist clozapine-N-oxide (CNO) twice a day for 11 weeks at the same time of transitioning into a high fat diet. Representative image shows the injection site. (**b**) Weight curve illustrating weight loss after stimulating DRN^Vglut3^ neurons upon DIO. (**c**) Adiposity assessment using magnetic resonance at the last day of treatment with CNO (n = 7 control group, 6 group HM3Dq). (**d**) Food intake during the chronic stimulation of DRN^Vglut3^ neurons demonstrating hypophagia (n = 7 control group, 6 group HM3Dq). (**e**) Adaptive thermogenesis at the last day of treatment with CNO (n = 7 control group, 6 group HM3Dq). (**f**) C ore body temperature assessment at the last day of treatment with CNO. (A-F) Littermate Vglut3-Cre positive DIO mice injected with an AAV2/5-DIO-mCherry were used as controls (n = 7 control group, 6 group HM3Dq). (**g**) Glucose tolerance test and area under the curve (AUC) of the intraperitoneal glucose (2g/kg) tolerance test during the preventive (CNO and high fat diet given at the same time) (2 left panels) and curative (CNO given after the development of DIO) (2 right panels) setting (n = 7 control group, 6 group HM3Dq). (**h**) Insulin tolerance test and AUC of the insulin tolerance test during the preventive (2 left panels) and curative (2 right panels) setting (n = 7 control group, 6 group HM3Dq). Data represented as Mean ± SEM. P-value calculated as a 2-way ANOVA with multiple comparisons using a Tukey post-hoc approach (B, G-H) or two-tailed unpaired t- test (C-F). Statistical difference established at p < 0.05. Source data

**Extended Data Fig. 4:**
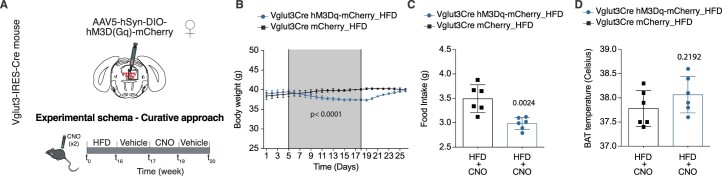
DRN^Vglut3^ Neurons Drive Reduces Both Food Intake and Body Weight in DIO Female Mice. (**a**) Schema illustrating chronic stimulation of DRN^Vglut3^ neurons in DIO female mice by injecting an AAV5-DIO-hm3Dq-mcherry in DRN^Vglut3^ mice using the DREADD agonist clozapine-N-oxide (CNO) once a day for 14 days after the development of obesity. Representative image shows the injection site. (**b**) Weight curve illustrating the body weight loss after stimulating DRN^Vglut3^ neurons upon DIO in female mice (n = 6 per group). (**c**) Food intake during the chronic stimulation of DRN^Vglut3^ neurons in females demonstrating hypophagia (n = 6 per group). (**d**) Adaptive thermogenesis at the last day of treatment with CNO (n = 6 per group). (A-D) Littermate Vglut3-Cre positive DIO mice injected with an AAV2/5-DIO-mCherry were used as controls. Data represented as Mean ± SEM. P-value calculated as a 2-way ANOVA with multiple comparisons using a Tukey post-hoc approach (B,), or two-tailed unpaired t-test (C, D). Statistical difference established at p < 0.05. Source data

Vglut3-IRES-Cre mice and littermate controls were fed a high-fat diet (HFD, 45% fat content, Research Diets) for 16 weeks, starting at 6 weeks of age. In brief, after obesity had developed in mice (at 22 weeks of age), we performed stereotactic injection of an excitatory DREADD (AAV5-hSyn-DIO-hM3D(Gq)-mCherry) into the DRN followed by treatment with CNO or saline (Fig. 2i–p). As an additional control, a separate group of Vglut3-IRES-Cre mice were injected with a Cre-dependent control virus (AAV5-hSyn-DIO-mCherry) and treated with CNO (Fig. 2i). Similar to its effect in ob/ob mice, chronic activation of DRN^Vglut3^ neurons led to a significant reduction of body weight in DIO mice (Fig. 2j) relative to control littermates (3.06% weight lost, *P* < 0.0001). Weight reduction was associated with a significant decrease in food intake (15.24% decrease, *P* = 0.0237, Fig. 2k) without effects on BAT thermogenesis (Fig. 2l). In a separate set of experiments, we also tested the ability of DRN^Vglut3^ activation to prevent obesity by beginning the CNO treatment at the same time as the animals were placed on the HFD. Similar to treatment after obesity had developed, DRN^Vglut3^ activation significantly reduced subsequent weight gain, beginning at the onset of HFD-induced weight gain (Extended Data Fig. 3a–d), with decreased adiposity and food intake but without thermogenic effects (Extended Data Fig. 3e,f). In line with the reduced weight, glucose tolerance tests performed during DRN^Vglut3^ activation showed a 22% decrease in the area under the curve both at HFD onset and after obesity had developed (*P* < 0.0001 and *P* = 0.0156, respectively). Insulin tolerance tests were unchanged between experimental groups (Extended Data Fig. 3g,h). Males and females exhibited similar results, thus ruling out sexual dimorphism (Extended Data Fig. 4a–d).

We next tested whether activation of DRN^Vglut3^ neurons in DIO animals alters systemic energy expenditure. To do so, a new cohort of Vglut3-IRES-Cre DIO mice (16 weeks on a HFD) was injected with AAV5-hSyn-DIO-hM3D(Gq)-mCherry or AAV5-hSyn-DIO-mCherry, and mice were then placed into metabolic cages to evaluate energy expenditure through indirect calorimetry. Activation of DRN^Vglut3^ neurons by injection of CNO led to a significant decrease in food intake in animals expressing the DREADD receptor, with no effect on locomotor activity (Fig. 2m), VO_2_ (Fig. 2n), VCO_2_ (Fig. 2o) or total energy expenditure (Fig. 2p). Here, regression analysis again showed a highly significant effect (*P* < 0.001) taking into account the body weight of each mouse using the CalR platform and ANCOVA statistical analyses^37^.

These data suggest that pharmacologic modulation of DRN^Vglut3^ neuronal activity using small molecules directed to cell-specific targets in those neurons may recapitulate the effect of chemogenetic modulation and similarly reduce weight in obese animals. We tested this by screening for druggable targets specifically expressed in these neurons.

### Identification of druggable targets in DRN^Vglut3^ neurons

**Fig. 3:**
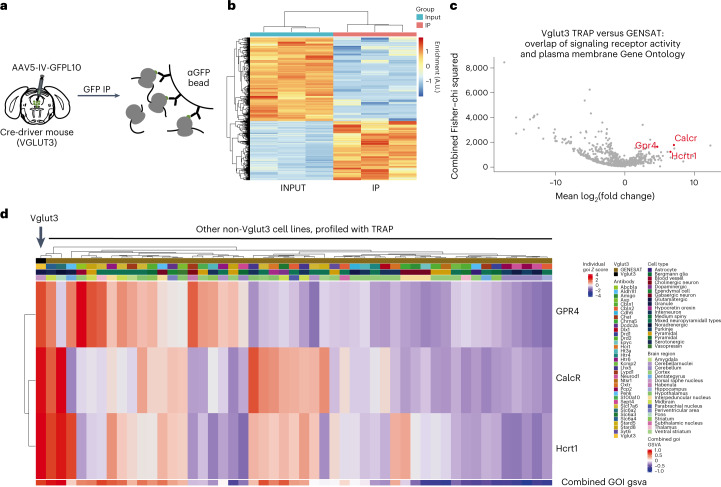
DRN^Vglut3^ neuronal profiling together with GENSAT comparison reveals three unique targets for drug discovery. **a**, Schematic illustrating the approach for AAV-mediated TRAP (vTRAP) studies using immunoprecipitation for ribosomal protein L-10 tagged with GFP (GFP-L10)^6^. **b**, Heat map of the three pairs of samples used for the identification of valuable druggable targets in DRN^Vglut3^ neurons, comparing immunoprecipitated RNA samples (IP) to each sample’s input RNA. **c**, Volcano plot showcasing the genetic markers enriched in the three IP samples in comparison to all the previously immunoprecipitated samples found in the TRAP-based GENSAT database. Additionally, two Gene Ontology filters were applied to screen for plasma-membrane-bound signaling receptors, narrowing down the list of interesting targets. Red dots highlight the differentially enriched markers of interest for small-molecule-based drug discovery in DRN^Vglut3^ cells (CalcR, Hcrtr1 and GPR4), after contrasting the data with the existing literature and in situ hybridization studies. **d**, Heatmap representing the expression levels of the three genes of interest (*calcR*, *hcrtr1* and *gar4*) in comparison to each individual immunoprecipitated sample from the TRAP experiments available in the GENSAT database, to highlight their higher expression in DRN Vglut3 cells. The legend is color coded for each TRAP driver line, cell type and brain region. The first column represents the IP data from DRN-Vglut3 neurons. Data are represented as enrichment in IP over input. *n* = 3 for IP/input TRAP experiments. The number of cell lines in GENSAT database contrasted (*n* = 50). Red indicates genetic overexpression, and blue indicates downregulation. GOI gsva refers to gene of interest gene set variation analysis. A.U., arbitrary units.

**Extended Data Fig. 5:**
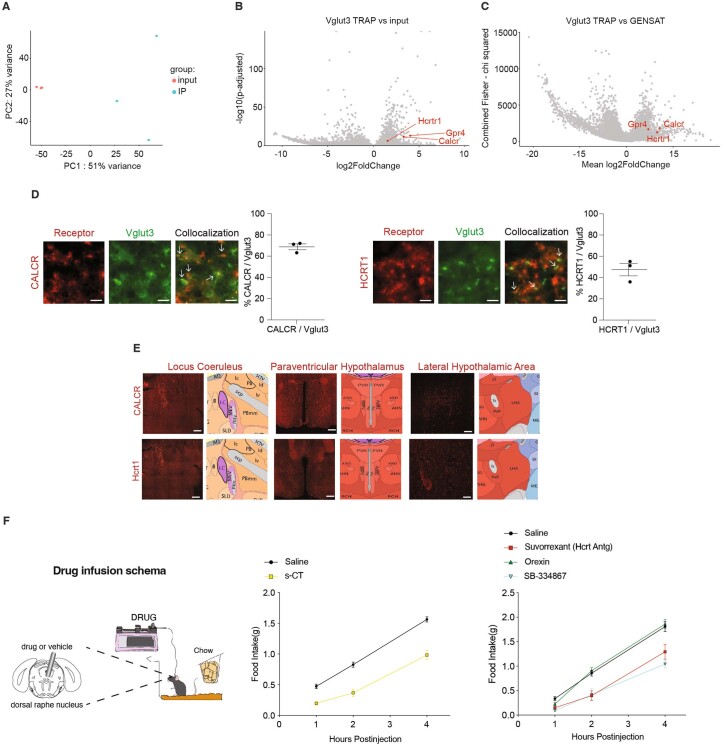
Translational strategy comparing DRN^Vglut3^ molecular profile to the GENSAT Translational Affinity Purification database reveals Druggable Targets for Weight Management. (**a**) Principal component analysis of the three pairs of samples used for the identification of valuable druggable targets in DRN^Vglut3^ neurons using TRAP. (**b**) Volcano plot for the adjusted p-value logarithmic plots versus the fold change logarithmic plots illustrating the set of markers most enriched in the IP sample versus the input. Red dots highlight differentially enriched markers for drug discovery that will be used to illustrate the enrichment after each filter is applied (CalcR, Hcrt1 and GPR4). (**c**) Volcano plot for the adjusted p-value logarithmic plots versus the fold change logarithmic plots illustrating the set of markers most enriched in the IP sample the GENSAT database. Red dots highlight differentially enriched markers for drug discovery that will be used to illustrate the enrichment after each filter is applied (CalcR, Hcrt1 and GPR4). (**d**) In situ hybridization studies showing colocalization between cell-type specific marker gene Vglut3 and receptors (CalcR or Hcrt1) in the Dorsal Raphe Nucleus. White arrows designate double-labeled cells (n = 3 per group). (**e**) In situ hybridization studies using RNAscope technology showing expression of the studied receptors in other brain locations such as the Locus Coeruleus, Paraventricular Hypothalmus and Lateral Hypothalamic Area. Scale bars 25um (**d**) 100um (**e**). (F-Left) Schema DRN infusion of drug ligands to CalcR and Hcrtr1 prior to feeding assessment. (F-middle) Salmon Calcitonin (30ng) infusion into the DRN as a direct ligand for CalcR shows feeding suppressing effects. (F-Right) Agonist (orexin (green)) (200ng) and antagonists (SB334867 (250ng) (cyan) and suvorexant (red)) (250ng) infused locally into the DRN exhibit feeding suppressant effects only at when orexin receptors are antagonized (n = 5 per group). Data are presented as mean ± SEM. Source data

To identify specific targets enriched in DRN^Vglut3^ neurons, the cell-specific transcription profiles of DRN^Vglut3^ neurons were generated using translating ribosomal affinity purification (TRAP)^39^ (Fig. 3a), followed by differential expression analysis of immunoprecipitated DRN^Vglut3^ RNAs versus the total RNA input (Fig. 3b and Extended Data Fig. 5a,b). These data included 17,234 expressed genes, of which 1,594 transcripts were significantly enriched (1.5-fold-enrichment with an adjusted *P* value (*p* < 0.05)) (Supplementary Table 2).

The DRN^Vglut3^ neuron IP samples were then compared with a comprehensive set of neuronal transcriptomes available at the GENSAT database (Extended Data Fig. 5c)^40^. Compared with other neural populations cataloged in the GENSAT database, 691 genes were enriched in DRN^Vglut3^ neurons (Supplementary Table 3). To identify druggable targets, we used the Gene Ontology browser^41^ database to identify which of these 691 transcripts were listed in both the signaling receptor activity (GO:0038023) and plasma membrane (GO:0005886) categories (MGI, informatics.jax.org (Fig. 3c)) After applying these filters, 27 druggable receptors were identified with more than 100-fold enrichment compared with the GENSAT database (highlighted in Supplementary Table 3). Next, we evaluated the brain-wide patterns of expression of those targets using the Allen Brain Atlas database^23^ to identify the most differentially expressed receptors in the DRN^Vglut3^ neurons compared with other cell types. Two neuropeptide receptors, calcitonin receptor (*calcr*), orexin receptor 1 (*hcrtr1*) and an orphan G protein-coupled receptor (*gpr4*) showed significantly higher expression in the DRN (highlighted in red in Fig. 3c and Extended Data Fig. 5b,c; Supplementary Tables 2–3). Consistent with this, aggregate differential expression analysis (Fig. 3c) and the relative expression (Fig. 3d) of these selected receptors, compared with molecular profiles of other cell types in the GENSAT database, reconfirmed the cell-specific enrichment of these genes in DRN^Vglut3^ neurons. (Absolute expression, *hcrtr1*: 9.52-fold enrichment, *calcr*: 55.01-fold enrichment; relative expression to GENSAT, *calcr*: 7.07 log_2_(fold enrichment), *hcrtr1*: 6.87 log_2_(fold enrichment).) We further validated the expression of *hcrtr1* and *calcr* using RNAscope in situ hybridization studies and quantitated their colocalization with Vglut3 (*slc17a8*) in the DRN (Extended Data Fig. 5d). In the DRN, there was 68.81% overlap of *calcr* with Vglut3 and 47.43% overlap between *hcrtr1* and Vglut3 (Extended Data Fig. 5e). In addition, 67.57% of *calcr*-expressing neurons express *slc17a8*, and 65.78% of Hcrtr1 neurons express *Slc17a8*. *calcr* and *hcrtr1* expression was also analyzed in serial coronal sections of brain and, consistent with the GENSAT profiling data, we found extremely limited expression of both genes outside the DRN, with only minimal expression in the LHA, PVH and DMH regions of the hypothalamus and in the locus coeruleus, and even sparser labeling in other brain regions, including the nucleus of the tractus solitarius (NTS) for *calcr* and the DG region of the hippocampus for *hcrtr1* (Extended Data Fig. 5e). On the basis of these data, we decided to next test the effect of Hcrtr1 and CalcR ligands on food intake and body weight. Gpr4 is an orphan G protein-coupled receptor, and because specific ligands are not currently available, it was not studied further.

### Drugs against DRN targets reduce body weight in DIO mice

Our objective was to identify drugs that activate DRN and test whether they replicate the effect of chemogenetic modulation of DRN^Vglut3^ neurons (see Fig. 2), and we set out to test this using salmon calcitonin (s-CT) and modulators of Hcrtr1. s-CT is a ligand for Calcr a G_s_-coupled receptor^42^. We note, however, that Calcr is also a component of the amylin receptor, and signaling through this heterotrimeric receptor is known to reduce food intake and body weight^43,44^. Thus, the endogenous ligand for Calcr in DRN is unclear. Hcrtr1 can be either G_s_-, G_q_- or G_i_-coupled, and although orexins are known to excite DRN neurons, the signal transduction pathway in DRN^Vglut3^ neurons specifically is unknown, we tested both Hcrtr1 agonists and antagonists^45,46^.

**Fig. 4:**
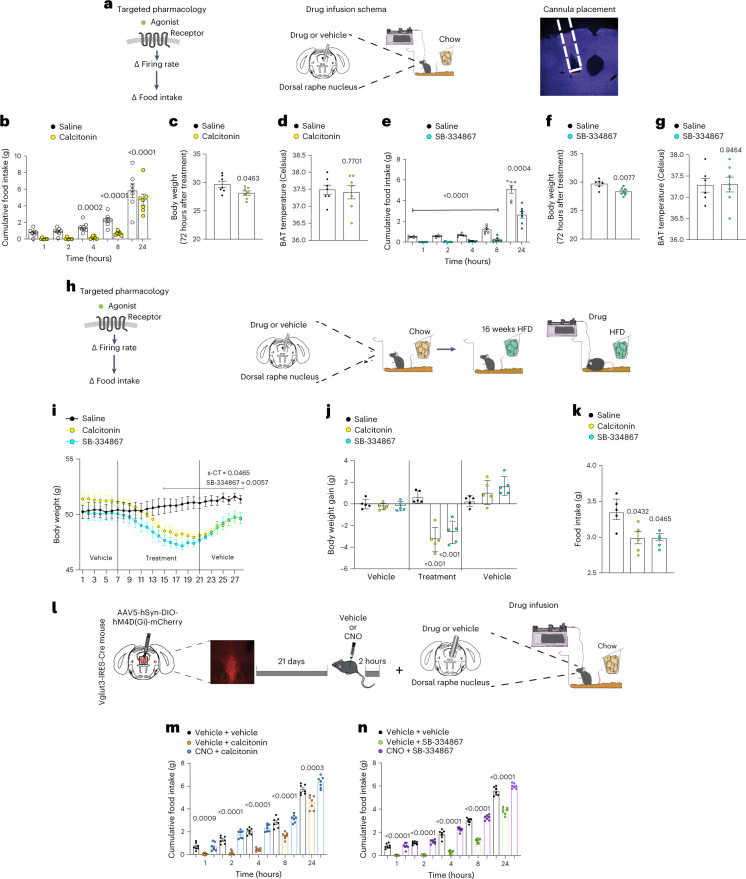
Local infusion (intra-DRN) of drug compounds to CalcR and Hcrtr1 ameliorates energy-balance defects after development of DIO. **a**, Left, schematic of profiling-based drug targeting. Middle, local intra-DRN drug-infusion schematic. Right, cannula placement validation. **b**, Acute chow-diet feeding assessment after a single dose of s-CT (30 ng) into the DRN (*n* = 7 per group). **c**, Weight assessment after 3 days of s-CT (30 ng) infusion in chow diet (*n* = 7 per group). **d**, Adaptive thermogenesis after DRN s-CT (30 ng) injection (*n* = 7 per group). **e**, Acute chow-diet feeding assessment after a single dose of SB-334867 (250 ng) into the DRN (*n* = 6 per group). **f**, Weight assessment after 3 days of SB-334867 (250 ng) infusion in chow diet (*n* = 6 per group). **g**, Adaptive thermogenesis after DRN SB-334867 (250 ng) injection (*n* = 6 per group). In **b**–**g**, saline-injected controls were used. **h**, Left, schematic of profiling-based drug targeting in mice fed a HFD. Right, schematic of drug infusion into the DRN of mice implanted with a cannula after 16 weeks of HFD feeding. **i**, Body weight after agonism of CalcR or antagonism of Hcrtr1 with s-CT (30 ng) or SB-334867 (250 ng) after intra-DRN infusion. Baseline measurements and post-treatment measurements were taken in vehicle-infused mice (7 days) (*n* = 5 per group, 2 degrees of freedom, *F* statistic 91.02, *P* = 0.0001). **j**, Weight gain after CalcR agonism or Hcrtr1 antagonism with s-CT (30 ng) or SB-334867 (250 ng) in mice in the pre-treatment, treatment and post-treatment phases (*n* = 5 per group). **k**, Food intake during the treatment window (14 days) of s-CT (30 ng) or SB-334867 (250 ng) (*n* = 6 per group). **l**, Schematic of profiling-based drug infusion in the DRN of s-CT/SB-334867 with or without DREADD inhibition of DRN Vglut3 cells. **m**,**n**, Cumulative food intake (*n* = 7 per group) of vehicle and s-CT (30 ng)- and SB-334867 (250 ng)-treated mice with and without CNO (*n* = 6 per group). Data are represented as mean ± s.e.m. *P* values were calculated using a two-way ANOVA with a multiple-comparisons test (Tukey post-hoc) (**i**), one-way ANOVA (Bonferroni post-hoc multiple comparisons) (**b**,**e**,**j**–**k**,**m**,**n**) or a two-tailed unpaired *t*-test (**c**,**d**,**f**,**g**). *P* < 0.05 was considered significant. Source data

**Extended Data Fig. 6:**
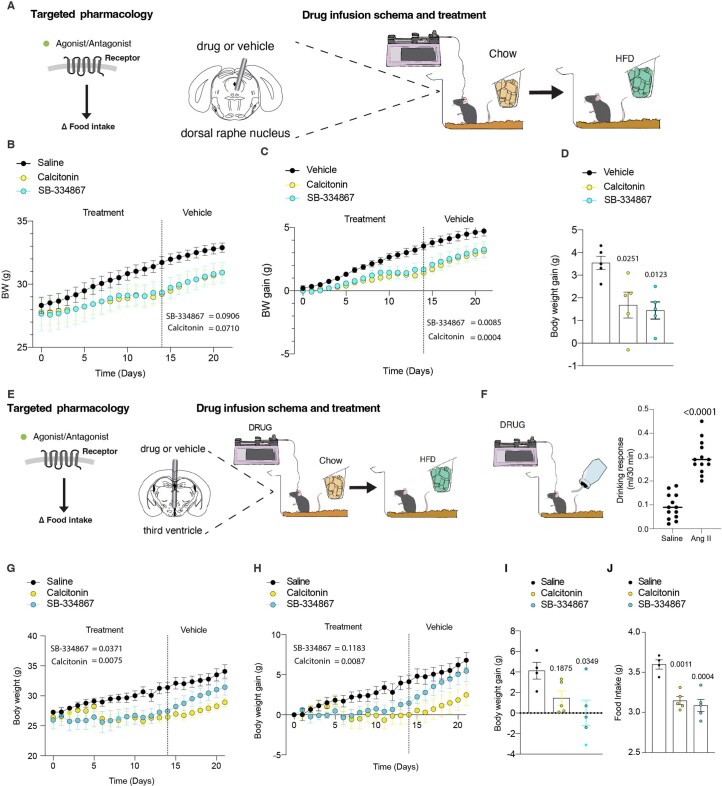
Local and Brain Wide Infusion in the Dorsal Raphe Nucleus of Drug Compounds to CalcR and Hcrtr1 (s-CT and SB-334867) Ameliorates Energy Balance Defects when transitioning from a standard Diet to HFD. **(a,Left)** Schema and profiling-based drug targeting. **(A, Right)** Drug infusion schema into the DRN of mice implanted with a local cannula to evaluate food intake when transitioning from chow diet to HFD (preventing obesity). **(b)** Weight curve illustrating the reduced body weight increase after CalcR agonism or Hcrtr1 antagonism with s-CT (30ng) or SB-334867 (250ng) in the DRN when transitioning from chow diet to HFD. Post-treatment mice are infused with vehicle (n = 5 per group). **(c)** Weight gain after CalcR agonism or Hcrtr1 antagonism with s-CT (30ng) or SB-334867 (250ng) compared to vehicle treated controls (n = 5 per group). **(d)** Food intake assessment during the treatment window of s-CT (30ng) or SB-334867 (250 ng) into the DRN (n = 5 per group). **(e)** ICV drug infusion schema of mice to evaluate feeding when transitioning from a chow diet to a HFD (preventing obesity). **(f)** Dipsogenic Angiotensin II infusion (10 ng) schematic for correct cannula placement evaluating drinking response (n = 13 per group). **(g)** Weight curve after ICV CalcR agonism or Hcrtr1 antagonism with s-CT (300ng) or SB-334867 (2.5 ug) respectively when transitioning from a chow diet to a HFD. Post-treatment mice are infused with vehicle (n = 5 per group). **(h)** Weight gain after CalcR agonism or Hcrtr1 antagonism with s-CT (300ng) or SB-334867 (2.5ug) compared to vehicle treated controls (n = 5 per group). **(i)** Weight gain during the treatment window (n = 5 per group). **(j)** Food intake assessment during the treatment window (14 days) of ICV s-CT (300ng) or SB-334867 (2.5 ug) infusion (n = 5 per group). Data represented as Mean ± SEM. P-value calculated as a 2-way ANOVA with multiple comparisons (Tukey post-hoc) (B-C, G-H), one-way ANOVA with Bonferroni post-hoc multiple comparison analysis (D, I-J) or two-tailed unpaired t-test (F). Statistical difference established at p < 0.05. Source data

We began by infusing s-CT or an Hcrtr agonist (orexin A) or Hcrtr antagonists (suvorrexant, SB-334867) directly into the DRN to test whether this could replicate the effect of chemogenetic activation (Fig. 4a and Extended Data Fig. 5f). Orexin A had no effect on feeding (Extended Data Fig. 5f). In contrast, s-CT (30 ng) infused into the DRN acutely reduced food intake in chow-fed mice (70.61% reduction in food intake compared to controls, *P* < 0.001). We then tested SB-334867 (250 ng), an Hcrtr antagonist with activity in vivo at both Hcrtr1 and Hcrtr2. Although this antagonist does show preferential binding to Hcrtr1, it also engages Hcrtr2 at the doses that are used in vivo, which leads to increased sleep^47^. Thus, at doses above 20 mg/kg intraperitoneally, this compound engages both Hcrtr1 and Hcrtr2 and induces somnolence^47^. SB-334867 significantly reduced food intake during a 24-hour infusion inside the DRN (SB-334867, 49.61% decrease in food intake compared with controls, *P* = 0.0004) (Fig. 4b,e and Extended Data Fig. 5f). s-CT and the Hcrtr antagonists also reduced body weight (calcitonin, 1.52 g of weight loss, *P* = 0.0463; SB-334867 ,1.36 g of weight loss, *P* = 0.0077) (Fig. 4c,f and Extended Data Fig. 5f). Consistent with our chemogenetic studies, BAT temperature did not change after the drug infusion (Fig. 4d,g). We next infused the same drugs into the DRN of DIO mice maintained on a HFD for 16 weeks and found that s-CT (30 ng) or SB-334867 (250 ng) infused directly into the DRN also reduced body weight (weight: controls 51.075 g, calcitonin 47.94 g and SB-334867 47.40 g, *P* = 0.0452 (calcitonin), *P* = 0.0057 (SB-334867)) (Fig. 4h–j) owing to reduced food intake (food intake: saline 3.374 g, s-CT 2.982 g and SB-334867 2.988 g, *P* = 0.0432 (calcitonin) and *P* = 0.0465 (SB-334867). We also found that s-CT and SB-334867 reduced body weight when treatment was initiated at the same time that the HFD was begun in a preventative paradigm (Extended Data Fig. 6a–d).

Finally, we confirmed that the effect of these drugs is dependent on DRN^Vglut3^ activation by simultaneously infusing either s-CT or SB-334867 into the DRN of Vglut3-Ires-Cre mice expressing an inhibitory DREADD (HM4Di) and simultaneously treating the mice with CNO intraperitoneally (i.p., 1 mg/kg) (Fig. 4l–n). We found that CNO pretreatment blocked the effects of either drug, confirming the role of DRN^Vglut3^ neurons in mediating this effect (Fig. 4l–n). In aggregate, these data show that engaging cellular targets in DRN^Vglut3^ in neurons can reduce food intake and body weight and replicate the effect of chemogenetic modulation of their activity.

### Intracerebroventricular infusion with DRN targets reduces weight in obesity

**Fig. 5:**
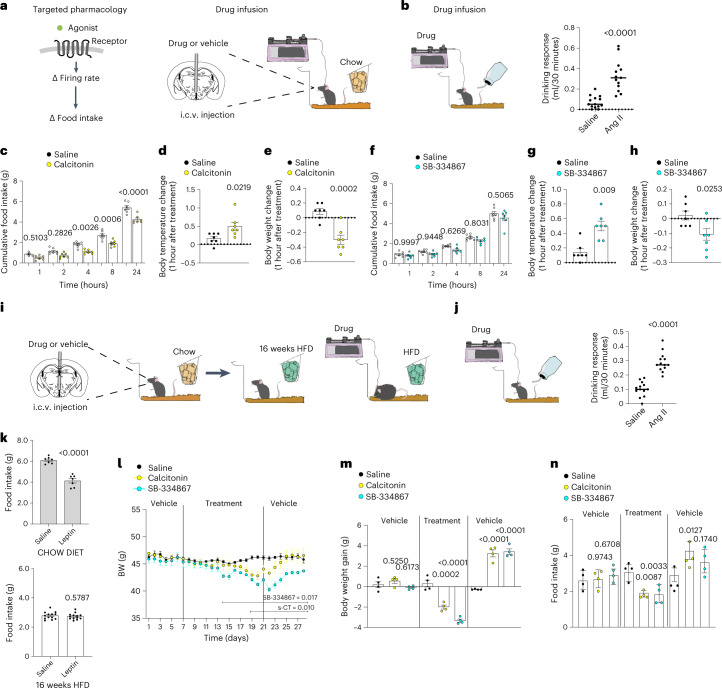
Intracerebroventricular brain-wide infusion of drug compounds to CalcR and Hcrtr1 ameliorates energy-balance defects upon DIO. **a**, Left, Schematic of profiling-based drug targeting. Middle, drug-infusion schematic for i.c.v. treatments. **b**, Dipsogenic angiotensin II infusion (10 ng) was used to verify cannula placement (*n* = 14). **c**, Acute chow-diet feeding assessment after a single i.c.v. dose of s-CT (300 ng) (*n* = 7). **d**, Adaptive thermogenesis after i.c.v. s-CT (300 ng) infusion (*n* = 7). **e**, Weight assessment after i.c.v. s-CT (300 ng) infusion in mice fed a chow diet (*n* = 7). **f**, Acute chow-diet feeding assessment after a single i.c.v. dose of SB-334867 (2.5 µg) (*n* = 7). **g**, Adaptive thermogenesis after i.c.v. SB-334867 (2.5 µg) infusion (*n* = 7). **h**, Weight assessment after i.c.v. SB-334867 (2.5 µg) infusion in mice fed a chow diet (*n* = 7). In **c**–**h**, saline-injected mice were used as controls. **i**, Drug-infusion schematic for i.c.v. treatments in mice after 16 weeks of HFD feeding. **j**, Dipsogenic angiotensin II infusion (10 ng) to control cannula placement for evaluating drinking response (*n* = 13). **k**, Twenty-four-hour food-intake assessment from a leptin-sensitivity assay (5 mg/kg) after 16 weeks of exposure to HFD (DIO) (*n* = 7). Chow-diet-fed mice were used as controls (*n* = 13). **l**, Weight after i.c.v. CalcR agonism or Hcrtr1 antagonism with s-CT (300 ng) or SB-334867 (2.5 µg), respectively. Pre-treatment baseline measurements and post-treatment measurements were taken in ACSF vehicle-infused mice for 7 days (2 degrees of freedom, *F* statistic 97.79, *P* = 0.0001) (*n* = 4). **m**, Weight gain after i.c.v. CalcR agonism or Hcrtr1 antagonism with s-CT (300 ng) or SB-334867 (2.5 µg), respectively (*n* = 4). **n**, Food-intake assessment during the i.c.v. treatment window (14 days) with s-CT (300 ng) or SB-334867 (2.5 µg) (*n* = 4). Data are represented as mean ± s.e.m. *P* values were calculated using a two-way ANOVA with a multiple-comparisons test (Tukey post-hoc) (**l**), one-way ANOVA with Bonferroni post-hoc multiple comparison analysis (**m**,**n**) or two-tailed unpaired *t*-test (**b**–**h**,**j**–**k**). *P* < 0.05 was considered significant. Source data

Our next objective was to identify whether infusion of these agents into the whole brain would mimic the effect of chemogenetically activating DRN^Vglut3^ neurons in DIO. To do so, we delivered the SB-334867 orexin receptor antagonist or s-CT through an intracerebroventricular (i.c.v.) injection (Fig. 5a). Correct cannula placement was verified by showing a dipsogenic response to angiotensin II (1 nmol in 1 µl)^48^ (Fig. 5b). Consistent with our prior data using local intra-DRN infusion, i.c.v. s-CT (300 ng) and SB-334867 (2.5 µg) injection led to reduced food intake in chow-fed wild-type mice (food intake 24 hours after infusion of s-CT 4.24 g; compared with artificial cerebrospinal fluid (ACSF)-infused controls, 5.40 g; *P* = 0.0004; food intake 8 hours after infusion of SB-334867, 2.27 g; compared with ACSF-infused controls, 2.61 g; *P* = 0.0470) (Fig. 5a,c,f). Core body temperature was not affected in s-CT-injected mice but was slightly increased in SB-334867-treated mice (Fig. 5d,g). Body weight also significantly decreased in chow-fed mice after i.c.v. treatment (body-weight loss 24 hours after infusion of s-CT −0.11 g, SB-334867 −0.30 g, compared with ACSF-infused controls 0.021 g (for s-CT), 0.086 g (for SB-334867), *P* = 0.0002 (s-CT), *P* = 0.0253 (SB-334867)) (Fig. 5e,h).

Next, cannulas were i.c.v. implanted to the lateral ventricle of DIO mice that had been fed a HFD for 16 weeks of age. Cannulas were i.c.v. implanted lateral ventricle of DIO mice that had been fed a HFD for 16 weeks (Fig. 5i). Cannula placement was again verified by showing a dipsogenic response to angiotensin II (1 nmol in 1 µl)^46^(Fig. 5j). Before assessing the effect of the drugs on DIO mice, we reconfirmed that they were leptin resistant and, as expected, found that a high i.p. dose of leptin (5 µg/g) did not reduce food intake in DIO mice but did reduce intake in chow-fed mice (Fig. 5k). We then infused s-CT and SB-334867 and found that both drugs reduced food intake and body weight in leptin-resistant DIO mice (weight: controls 46.25 g, s-CT 42.80 g and SB-334867 41.20 g, *P* = 0.001 (calcitonin), *P* < 0.0001 (SB-334867)) (Fig. 5l–n). Similarly, there was also a significant decrease of food intake and body weight (Extended Data Fig. 6e–j), when chronic i.c.v. infusions of these drugs were initiated at the same time as the mice were placed on a HFD (that is, a preventative study).

**Extended Data Fig. 7:**
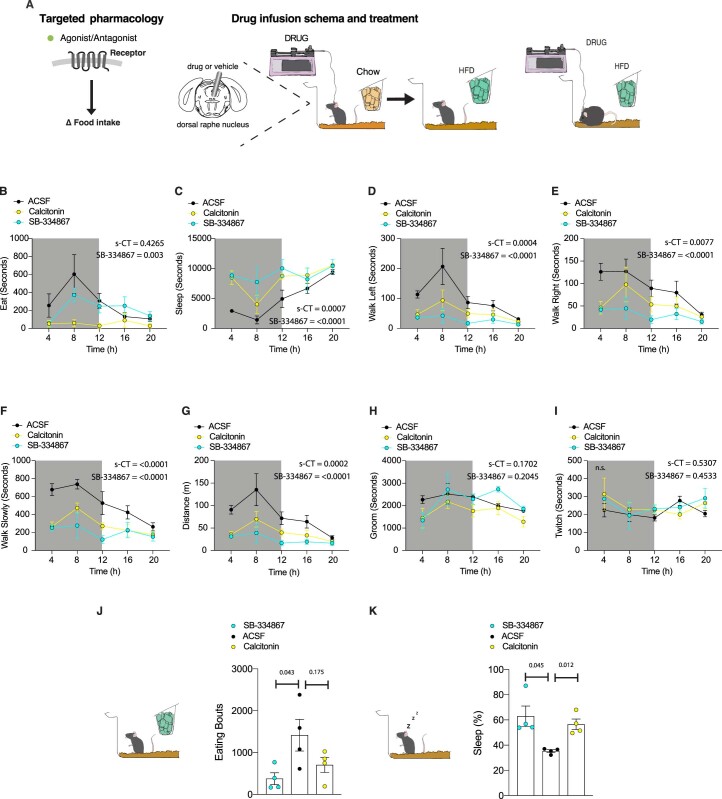
Infusion to the Dorsal Raphe Nucleus of s-CT and SB-334867 Does Not Induce Major Side Effects in Mice. **(a)** Drug infusion schema into DRN of mice implanted with a cannula to evaluate food intake effects chronically after induction of DIO. **(B-I)** 20-hour automated cage observation of mice behavior in a clear glass cage to evaluate whether s-CT or SB-334867 lead to debilitating side effects in the DRN (n = 4 per group). Behavioral paradigms evaluated are time spent **(b)** Ingesting food and **(c)** Sleeping. In addition, automated system evaluates the locomotion activity of mice as time spent **(d)** Walking Left, **(e)** Walking Right, **(f)** Walking Slowly or ambulating. As a function of that parameter, it provides the **(g)** Total Distance Travelled. Recordings provide information regarding time spent **(h)** Grooming or **(i)** Twitching in the behavioral cage which would be associated to stress behaviors. **(j-k)** Information about eating and sleeping is also calculated as amount of eating and sleeping bouts. Total eating and sleeping bouts of mice treated with ACSF, s-CT and SB- 334867. In line with our manually acquired data feeding suppression is observed in both drug treatments **(j)** and in line with previous literature there is an enhancement of sleeping time in both treatments. Data are represented as Mean ± SEM. P-value calculated as a 2-way ANOVA with multiple comparisons (Tukey post- hoc approach) (B-I), one-way ANOVA with Bonferroni post-hoc multiple comparison analysis (J-K). Statistical difference established at p < 0.05. Source data

Neither drug was associated with overt adverse effects, as assessed using an automated system for behavioral testing that monitors grooming, twitching and motor behaviors (Extended Data Fig. 7a–i). These automated assays reconfirmed the reduction in food intake (food intake: 1,415 eating bouts (controls), 703.3 eating bouts (calcitonin) and 375.3 eating bouts (SB-334867), *P* = 0.175 (calcitonin), *P* = 0.0436 (SB-334867)) (Extended Data Fig. 7j). However, both drugs led to a significant increase in time spent sleeping (controls slept 35.31% of time, calcitonin-treated mice 56.58% of time and SB-334867-treated mice 62.99% of time, *P* = 0.0457 (calcitonin), *P* = 0.0122 (SB-334867)) (Extended Data Fig. 7k). This increased sleep time was also associated with a decrease in locomotor activity (Extended Data Fig. 7d,e). Effects of s-CT on sleep have been previously reported^49^. The induction of sleep is also an on-target effect of antagonizing Hcrtr2 and, as mentioned, SB-334867 can engage Hcrtr2 in vivo at the tested dose^50^. Since mice with Hcrtr2 knockout show increased sleep time and narcolepsy, these data suggest that the effects SB-334867 on sleep are mediated by the Hcrtr2 receptor. If true, a highly selective Hcrtr1 antagonist that does not engage Hcrtr2 in vivo would reduce weight without inducing sleep. We next tested this possibility using a novel Hcrtr1 antagonist developed by Cerevance.

### CVN45502 reduces food intake and body weight in DIO

**Fig. 6:**
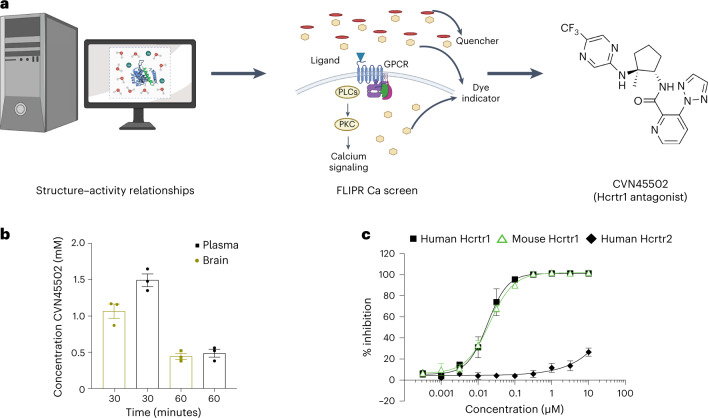
Pursuing Hcrtr1 antagonism through a specific Hcrt1 antagonist, CVN45502, is a potential strategy for weight management. **a**, Strategy and chemical structure, synthesized by Cerevance, from a highly selective orexin 1 receptor (Hcrtr1) antagonist CVN45502 using a combination of structure-activity relationships and a FLIPR Ca screen. **b**, Pharmacokinetic assessment of brain penetration for compound CVN45502 when given orally to mice, demonstrating a high brain penetrance in a 60-minute window (*n* = 3 mice). **c**, In vitro pharmacokinetic percentage of receptor inhibition for test compound CVN45502 when using in vitro assays expressing murine or human Hcrtr1 as well as human Hcrtr2. The pharmacokinetic panel shows binding to the murine and human Hcrtr1 receptor without binding to Hcrtr2 at therapeutic doses. Pharmacokinetics assay pinpoints to concentrations from 0.1 µM or greater to inhibit 100% of cells expressing Hcrt1, thus validating the dosing used of 30 mg/kg (*n* = 4 mice). Data are represented as mean ± s.e.m. *P* values were calculated using an unpaired *t*-test (**b**). *P* < 0.05 was considered significant. Figure 6a was performed with Biorender. Source data

CVN45502 is a highly selective Hcrtr1 antagonist that was developed from a high-throughput screen^51^ (Fig. 6a). After hits from the screen were selected, a medicinal chemistry campaign identified CVN45502 as a compound that had drug-like properties with central nervous system penetration suitable for clinical development (unpublished data). Pharmacokinetic profiling showed that CVN45502 is highly brain penetrant with a concentration in the brain of 1.1 ± 0.17 µM 30 minutes after a 3 mg/kg dose and a brain to plasma ratio of 0.7 at 30 minutes and 0.9 at 60 minutes (Fig. 6b). Of note, CVN45502 has a plasma protein binding in mice of 84.4%. Next, to determine the functional activity of CVN45502, Chem-1 cells stably expressing human Hcrtr1 or Hcrtr2 were assayed. CVN45502 fully inhibited the intracellular calcium released in cells expressing Hcrtr1 in response to orexin A (EC_80_ (drug concentration that elicits 80% of *E*_max_)) in a concentration-dependent manner, with a half-maximal inhibitory concentration (IC_50_) of 0.02 µM (Fig. 6c). In contrast, CVN45502 had a negligible effect on Ca^2+^ flux elicited by orexin A (EC_80_) in the Hcrtr2-expressing cell line across a wide concentration range (IC_50_ > 10 µM) (Fig. 6c). Further supporting these functional data, binding affinity (p*K*_i_) was –log_10_(7.86) ± 0.01 and –log_10_(4.66) ± 0.01 for the human Hcrtr1 and Hcrtr2 receptors, respectively. p*K*_i_ is –log_10_(*K*_i_), indicating that the selectivity of CVN45502 for human Hcrtr1 relative to Hcrtr2 in a functional assay is greater by three orders of magnitude (>1,000-fold). Cross-species activity for mouse was determined by expressing mouse Hcrtr1 in CHO-K1 cells and following the same protocol as described above for the human receptor assays. For mouse Hcrtr1, the IC_50_ was calculated as 0.018 µM, correlating well with that for the human receptor (Fig. 6c). CVN45502 (10 µM) was also screened against a broad panel of 125 receptors, ion channels, enzymes and transporters that are targets for approved drugs (Cerep, Eurofins) (Supplementary Table 4). There was less than 50% activity at all targets in the screen, indicating that CVN45502 does not engage any of these other drug targets and is highly specific for Hcrtr1.

**Fig. 7:**
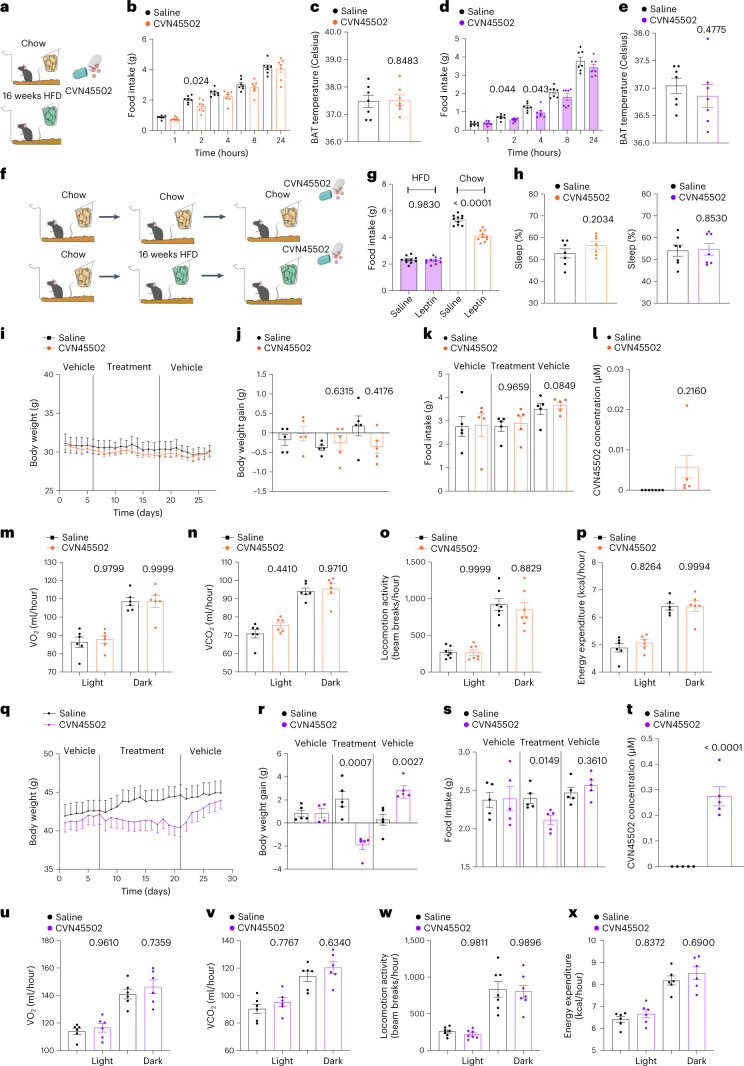
Chronic oral treatment with CVN45502 ameliorates energy-balance defects after DIO. **a**, Schematic of oral delivery of CVN45502 (30 mg/kg) in peanut butter in chow-diet-fed or HFD-fed mice. **b**, Acute chow diet feeding after CVN45502 (30 mg/kg) treatment (*n* = 7). **c**, Adaptive thermogenesis after CVN45502 (30 mg/kg) treatment in mice fed a chow diet (*n* = 7). **d**, Acute HFD feeding assessment after CVN45502 treatment (30 mg/kg) (*n* = 7). **e**, Adaptive thermogenesis after oral delivery of CVN45502 (30 mg/kg) to mice fed a HFD (*n* = 7). **f**, Schema for oral delivery of CVN45502 (30 mg/kg) after induction of DIO (16 weeks in HFD). Chow-diet-fed mice were used as controls. **g**, Feeding assessment from a leptin-sensitivity assay (5 mg/kg) in DIO and chow-diet controls (*n* = 10). **h**, Sleep assessment after CVN45502 treatment (30 mg/kg) (*n* = 10). **i**, Weight measurement following CVN45502 treatment (30 mg/kg) in chow-diet-fed mice. Pre-treatment and post-treatment measurements were taken in vehicle-treated mice (*n* = 5). **j**, Weight gain after Hcrtr1 antagonism with CVN45502 (30 mg/kg) compared with controls in chow diet (*n* = 5). **k**, Food intake after CVN45502 (30 mg/kg) treatment in chow-diet-fed mice (*n* = 5). **l**, Plasma levels of CVN45502 12 hours after treatment with CVN45502 (30 mg/kg) (*n* = 5). **m**–**p**, Indirect calorimetry assessment in chow-diet-fed mice (*n* = 7) using metabolic cages. The parameters measured are oxygen consumption (**m**), CO_2_ production (**n**), locomotor activity (**o**) and energy expenditure (**p**). **q**, Weight curve after Hcrtr1 antagonism using CVN45502 (30 mg/kg) in DIO mice (*n* = 5). **r**, Weight gain after Hcrtr1 antagonism using CVN45502 (30 mg/kg) compared with vehicle-treated controls in DIO mice (*n* = 5). **s**, Food intake after Hcrtr1 antagonism using CVN45502 (30 mg/kg) in DIO mice (*n* = 5). **t**, Plasma levels of CVN45502 12 hours after treatment with CVN45502 (30 mg/kg) (*n* = 5). **u**–**x**, Indirect calorimetry assessment in DIO mice using metabolic cages. Parameters measured are oxygen consumption (**u**) (*n* = 6), CO_2_ production (**v**) (*n* = 6), locomotor activity (**w**) (*n* = 7) and energy expenditure (**x**) (*n* = 6). Data are represented as mean ± s.e.m. *P* values were calculated using a two-way ANOVA with a multiple-comparisons test (Tukey post hoc) (**j**,**k**,**r**,**s**), one-way ANOVA (b,**d,g**) or two-tailed unpaired *t*-test (**c**,**e**,**h**,l,**t**), or using CalR software and ANCOVA regression analysis taking body weight into account (**i**–**l**,**q**–**t**). *P* < 0.05 was considered significant. Source data

We next assessed the therapeutic potential of this compound for treating obesity by feeding mice CVN45502 mixed into a 0.1 g peanut butter pellet in preclinical studies of mice fed either a chow diet or DIO mice on a HFD for 16 weeks (Fig. 7a). Mice that received only peanut butter without CVN45502 were used as controls. We first measured food intake and BAT thermogenesis acutely over the course of 24 hours and found that CVN45502 significantly reduced food intake at 2 hours post-treatment in chow-fed mice (Fig. 7b) and from 2–4 hours in the DIO mice (Fig. 7d). As was also the case after chemogenetic activation of DRN^Vglut3^ neurons, effects on BAT thermogenesis were not observed (Fig. 7c,e). We then repeated these studies over the course of 14 days (Fig. 7f). Before assessing the effect of the drugs on DIO mice, we first reconfirmed that the mice were leptin resistant. Consistent with previous reports, a high i.p. dose of leptin (5 µg/g) reduced food intake in lean, chow-fed mice, but did not alter food intake in DIO mice (Fig. 7g).

Food intake and body weight were measured at three time points: (1) after 7 days of peanut butter administration alone; (2) after a 14-day treatment of peanut butter with CVN45502 versus controls that received peanut butter alone; and (3) after a 7-day wash-out period, during which the mice received only peanut butter (Fig. 7i,q). There was no change in weight in either group during the 7-day lead in which animals received peanut butter alone.

However, after 14 days of treatment with CVN45502 (30 mg/kg), we observed a significant reduction of food intake and body weight in DIO mice (body weight gain: vehicle-treated 2.08 g, CVN45502-treated −1.92 g, *P* = 0.0007; Fig. 7q,r) (food intake: vehicle-treated 2.39 g, CVN45502-treated 2.11 g, *P* = 0.0149; Fig. 7s). In contrast, neither body weight nor food intake were significantly reduced in the chow-fed controls (Fig. 7i–l). Next, we confirmed that there was a relationship between weight loss and CVN45502 exposure in DIO mice by analyzing the drug level in a blood sample taken 12 hours after the final dose (day 7). At this time point, the mice still had a total plasma level of 0.26 ± 0.1 µM, which is significantly greater than the drug’s IC_50_ of 0.02 µM (Fig. 7m,t).

We next tested whether treatment of DIO mice with CVN45502 alters energy expenditure. Separate cohorts of DIO mice (on a HFD for 16 weeks) and lean chow-fed mice were treated with peanut butter or peanut butter with CVN45502 (30 mg/kg), as described above. Mice were next placed into metabolic cages for automated phenotyping and indirect calorimetry. Consistent with the previous data, CVN45502 led to a significant decrease in food intake assayed using the automated system, with no effect on VO_2_ (Fig. 7m,u), VCO_2_ (Fig. 7n,v), total energy expenditure (Fig. 7p,x) or locomotor activity (Fig. 7o,w). In contrast to the studies using SB-334867 the less selective hypocretin antagonist, mice treated with CVN45502 (30 mg/kg) did not show an alteration of total sleep time or locomotor activity, providing functional evidence for its selectivity for Hcrtr1 instead of Hcrtr2 (Fig. 7h).

**Extended Data Fig. 8:**
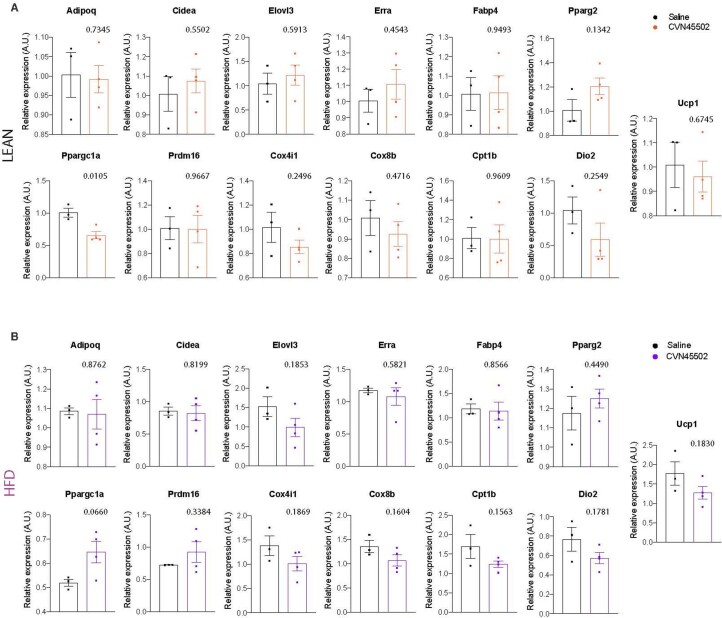
Treatment with CVN45502 (30 mg/kg) does not lead to major changes in gene expression of brown adipose tissue thermogenesis markers. **(a)** Normalized gene expression of thermogenic, brown adipose and mitochondrial genes of brown adipose samples collected from mice treated with CVN45502 (30 mg/kg) during 7 days and fed a standard chow diet. Genes selected have been extensively validated as key markers for thermogenic processes in the BAT. PPARgc1a is the only marker exhibiting a reduction in gene expression suggesting no major changes exist in BAT functionality (n = 3 control group, n = 4 CVN45502 group). **(b)** Normalized gene expression of thermogenic, brown adipose and mitochondrial genes of brown adipose samples collected from mice treated with CVN45502 (30 mg/kg) during 7 days and fed a high fat diet during 16 weeks to develop diet induced obesity. Genes selected have been extensively validated as key markers for thermogenic processes in the BAT. None of the gene expression markers exhibit a significant difference in gene expression suggesting no major changes exist in BAT functionality (n = 3 control group, n = 4 CVN45502 group). Data represented as Mean ± SEM. P-value calculated as one-way ANOVA with Tukey post hoc assessment. Statistical difference established at p < 0.05. Source data

In these studies, we first confirmed that none of these effects were seen after treatment of chow-fed or DIO mice that were given peanut butter alone. Regression analysis took into account each animal’s body weight, and the statistical analysis was performed using the CalR platform and ANCOVA statistical analysis^37^. Consistent with a lack of effects on BAT thermogenesis and indirect calorimetry, treatment with CVN45502 did not alter the mRNA levels of markers of iBAT thermogenesis either in chow-fed (Extended Data Fig. 8a) or DIO mice (Extended Data Fig. 8b).

**Extended Data Fig. 9:**
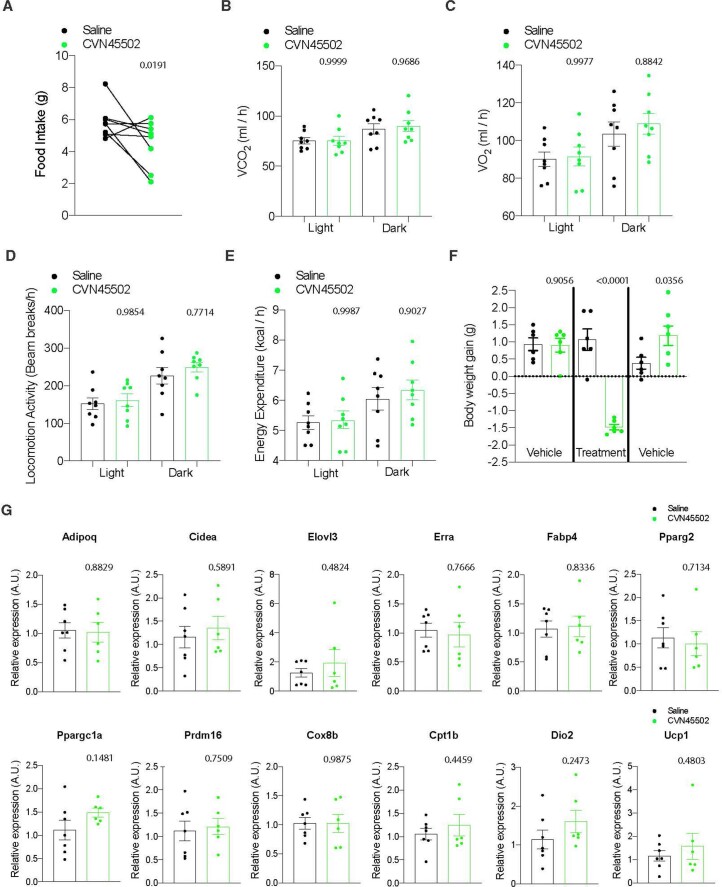
Treatment with CVN45502 (30 mg/kg) leads to a reduction in food intake with no major changes in energy expenditure in leptin deficient genetic obese ob/ob mice. **(a-f)** Energy balance phenotyping using a home-cage metabolic cage system. Parameters measured in a day/night cycle were (A) feeding, (B) CO2 production, (C) oxygen consumption, (D) locomotor activity and, (E) energy expenditure (n = 7 control group, n = 8 CVN45502 group). Significant changes supportive of weight loss only existed in food intake, similar to CVN45502 treatment in DIO mice **(**Fig. 7**)**. **(f)** body weight gain of ob/ob mice treated with CVN45502 (n = 6 control group, n = 5 CVN45502 group). **(g)** Normalized gene expression of thermogenic, brown adipose and mitochondrial genes of brown adipose samples collected from mice treated with CVN45502 (30 mg/kg) during 7 days and fed a standard chow diet. Genes selected have been extensively validated as key markers for thermogenic processes in the BAT. None of the gene expression markers exhibit a significant difference in gene expression suggesting no major changes exist in BAT functionality (n = 7 control group, n = 6 CVN45502 group). Data represented as Mean ± SEM. P-value calculated using CalR software and ANCOVA regression analysis taking body weight into account (A-E) or two-way ANOVA with repeated measures and Tukey post hoc assessment. p < 0.05 is considered significant. Source data

We next assessed the effect of CVN45502 (30 mg/kg) in leptin-deficient ob/ob mice using the indirect calorimetry system to measure both food intake and energy expenditure over the course of a 3-day lead with vehicle treatment, followed by 7 days of treatment with CVN45502 (30 mg/kg). CVN45502 significantly reduced food intake in ob/ob mice (vehicle-treated 5.865 g ± 0.3739; CVN45586 treated 4.263 g ± 0.4759) (*P* = 0.0403) (Extended Data Fig. 9a). In contrast, there was no effect on oxygen consumption, CO_2_ production, locomotion activity or energy expenditure (Extended Data Fig. 9b–e). In line with the feeding-suppressing effects of CVN45502 in ob/ob mice, 10 days of treatment significantly reduced body weight (vehicle-treated, 1.067 g ± 0.7607; CVN45586-treated, −1.483 g ± 0.1941) (Extended Data Fig. 9f). Of note, levels of RNA for a set of thermogenic genes expressed in BAT remained unaltered (Extended Data Fig. 9g), further arguing for a lack of thermogenic responses. In total, these data show that CVN45502 (30 mg/kg) can reduce the weight of leptin-resistant DIO and leptin-deficient ob/ob mice.

**Fig. 8:**
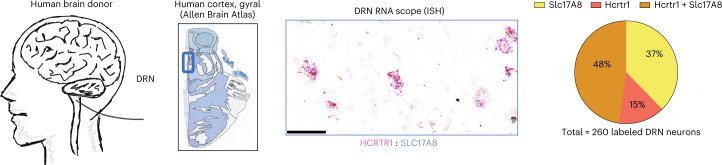
In situ hybridization in human donor samples reveals colocalization of both Hcrtr1 and Vglut3 in the DRN. Left, illustration representing human brain donors for ISH testing. Brain samples from three human donors were used. Middle, multiplex ISH studies using RNAscope of Hcrtr1 and Vglut3 (Slc17a8) in DRN sections of human donors, confirming co-labeling of Hcrtr1 and Vglut3 in the DRN and recapitulating our mouse studies in humans. Right, quantification of ISH RNAscope studies demonstrating a 48% overlap between Slc17A8 and Hcrtr1 (orange). 37% of the neurons counted were only Slc17A8-expressing (yellow), and 15% only expressed Hcrtr1 (red). Scale bar, 50 µm. Source data

**Extended Data Fig. 10:**
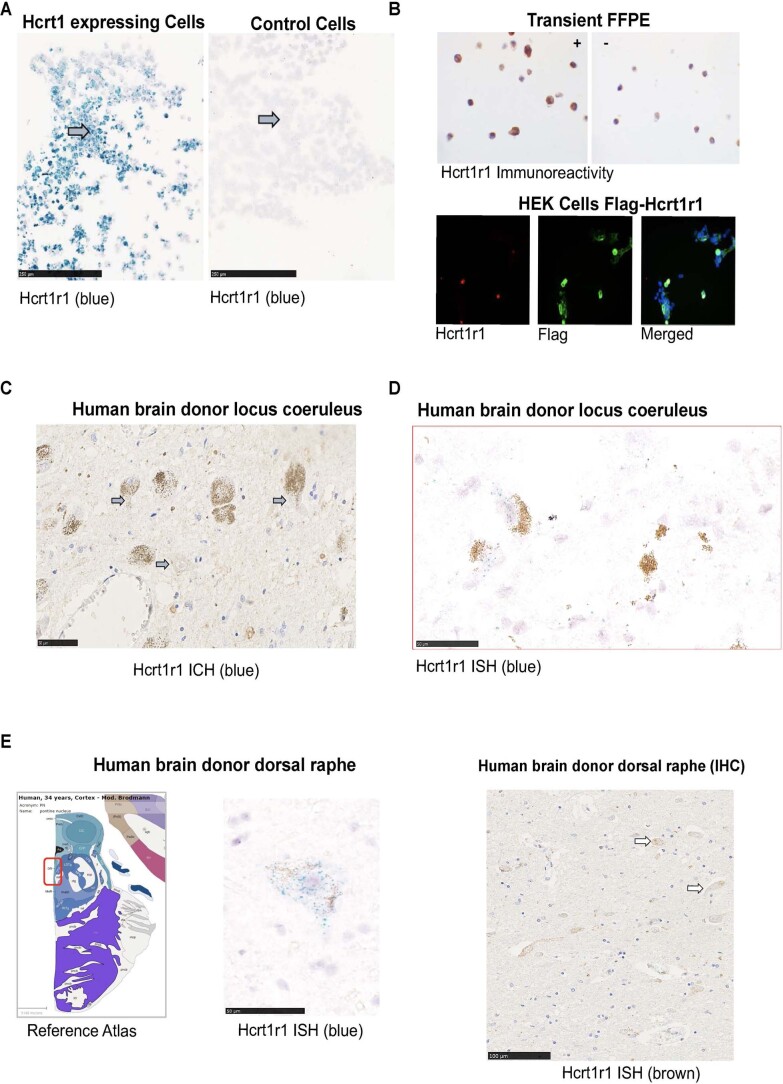
Hcrt1 is expressed in the DRN and colocalizes with Vglut3 in humans. . **(a)** In vitro studies in chemical competent cells (control) as well as chemical competent cells transfected with Hcrtr1 to validate the specificity of the probe used (Staining in blue). **(b)** In vitro immunohistochemistry (IHC) study (top) and immunofluorescence (IF) study (bottom) in Formalin Fixed Paraffin Embedded HEK 293 cells expressing Hcrt1 demonstrating antibody specificity to move forward with human anatomy studies with the selected antibody. The cells used in the bottom image are Hcrt1-FLAG-tagged HEKs. Cells were observed with a 20-fold objective lens. **(c)** Immunohistochemistry of Hcrtr1 in brain tissue of human donor at the Locus Coeruleus region. Extensive evidence shows Hcrtr1 expression in this region of the brain, hence this data validates in vivo the selected antibody. **(d)** In situ hybridization studies using an RNAscope probe for human Hcrtr1 in the locus coeruleus of human donors. This data validates the selected probe for studies in the DRN. **(e)** In situ hybridization studies staining for Hcrtr1 in DRN sections of human donors confirming labelling of Hcrtr1 in this brainstem region and recapitulating our initial starting point of this study, the DRN, in humans (Left). Immunohistochemistry of Hcrtr1 in brain tissue of human donor at the DRN region further demonstrating expression of Hcrtr1, using a validated antibody for Hcrtr1 (Right). All imaging studies were performed in 3 biological independent experiments in all cell culture or mice work. Scale bars = 50–100 um. Source data

Finally, to further evaluate whether Hcrtr1 is also colocalized in DRN^Vglut3^ cells, in humans we performed immunofluorescence (IF), immunohistochemistry (IHC) and in situ hybridization (ISH) analyses of human brain using a well-validated Hcrtr1 antibody against the third cytoplasmic domain of the human Hcrtr1 receptor together with RNA probes for Vglut3 and Hcrtr1. We validated these reagents in HEK cell lines transfected with the human Hcrtr1 receptor and confirmed the specificity of the antibody (Extended Data Fig. 10a,b). We next assayed the expression of Hcrtr1 in the locus coeruleus, a site with Hcrtr1 expression in human tissue, and confirmed a high level of Hcrtr1 expression (Extended Data Fig. 10c,d). Next, we analyzed the level of Hcrtr1 expression in DRN tissue from human donors (Fig. 8, left) using the aforementioned IHC/ISH assays for both Vglut3 and Hcrtr1 (Extended Data Fig. 10e) and found Vglut3 and Hcrtr1 co-labeling in the same cells (Fig. 8, middle). Quantitative assessment was performed using the Paxinos Atlas of the human brainstem following the ventral extension of the midline, and we found a 48% overlap between Vglut3 and Hcrtr1 (total number of labeled neurons: 260, Slc17A8 (Vglut3): 98, Hcrtr1: 38, Slc17A8 + Hcrtr1: 124) (Fig. 8, right). Together, these results demonstrate co-expression of these genes in human DRN^Vglut3^ neurons, recapitulating our in situ results in mice (Extended Data Fig. 5d) and further that suggesting Hcrtr1-specific antagonism using CVN45502 could represent a potential therapeutic approach for treating obesity in people.

## Discussion

Food intake is regulated by a distributed network of interacting neuronal clusters^16,52,53^. Two canonical centers that control appetite are the hypothalamus^15,54^ and the brainstem^15^, both of which process that report nutritional and other information pertinent to feeding^15,55^. An important interoceptive signal is the hormone leptin, which is secreted by adipocytes and serves as the afferent signal in a negative feedback loop that stably mantains adipose tissue mass^17^. Leptin reduces food intake and increases energy expenditure, and it modulates numerous other physiologic processes by binding to a receptor that is expressed at high levels in the ARC of the hypothalamus and other brain regions. Although some people with obesity hypo-secrete leptin, most have high endogenous hormone levels (a hallmark of obesity) and show a diminished response to leptin administration^17^. This suggests that obesity is generally the result of an insensitivity to high circulating levels of leptin, also known as leptin resistance^14^. In the majority of cases, neither the molecular lesion nor the relevant anatomic site leading to leptin resistance is known, and in principle, the block could be anywhere in the neural circuit downstream of leptin-sensitive neurons. However, although it is not yet possible to target the primary lesion, an alternative strategy for treating obesity would be modulating the activity of neurons regulating feeding that are downstream of the site(s) of leptin resistance. Here, we show that chemogenetic and pharmacologic activation of Vglut3-expressing neurons in the DRN can reduce body weight in leptin-deficient ob/ob mice and leptin-resistant DIO mice, indicating that these neurons are indeed functionally downstream of leptin signal.

These studies built on previous results showing that different neural populations in the DRN of the brainstem can bidirectionally control food intake^6,56–58^, locomotion^6^ and thermogenesis^7^. We found that DRN neurons expressing the GABAergic marker Slc32a1 (DRN^Vgat^) are activated by fasting and DRN neurons expressing the glutamatergic marker Slc17a8 (DRN^Vglut3^) are activated by refeeding^6^. These two neuronal populations reciprocally control food intake and locomotion^6,7^. Moreover, acute activation of DRN^Vglut3^ neurons or inhibition of DRN^Vgat^ neurons in leptin-deficient ob/ob mice suppresses food intake and augments locomotor activity^6^. These data demonstrate that these neurons regulate food intake in a leptin-independent manner, thus raising the possibility that they are downstream of the site of the block in the leptin-resistant state. Consistent with the DRN being downstream of leptin signaling, we find inputs to DRN from ARC^5^, the canonical site of leptin action, but failed to observe DRN^Vglut3^ projections to this site. Rather, we find functional projections of DRN^Vglut3^ neurons to the LHA, as well as the BLA and PBN, each of which has been previously shown to regulate feeding^30–32^. Thus, the DRN appears to be a component of a subcortical circuit that controls feeding and sends functional projections to anatomic sites that regulate homeostatic feeding in the LHA, as well as projecting to the PBN, which reduces food intake in response to aversive signals. The projection mapping further suggests that DRN^Vglut3^ neurons activate a Vglut2 population in the LHA that also reduces feeding. DRN^Vglut3^ neurons also project widely to cortical layers in a pattern not dissimilar to that of POMC neurons expressing LepR, although it is unclear whether there is crosstalk between DRN^Vglut3^ neurons and leptin at these sites.

On the basis of this, we then tested whether DRN^Vglut3^ neurons act independently of leptin action by using chemogenetics to chronically activate DRN^Vglut3^ neurons in leptin-deficient ob/ob and leptin-resistant DIO mice. We found that DRN^Vglut3^ activation significantly lowers body weight in both groups, confirming that these neurons regulate energy balance downstream of (or independently of) leptin signaling. We further explored the possibility that DRN^Vglut3^ modulation could reduce body weight in obese animals by first identifying cell-specific targets in comparisons of molecular profiling data for DRN^Vglut3^ neurons to 50 other cell types neuronal in the GENSAT database^39^. We thus identified the Hcrtr1 orexin receptor as a potential drug target expressed in DRN^Vglut3^ neurons. Orexin, also known as hypocretin, is the peptide ligand for Hcrtr1 and Hcrtr2. Orexin plays an important role in controlling arousal, stress and reward. Dual orexin receptor antagonists have been approved by the US Food and Drug Administration (FDA) to promote sleep, although studies using mouse receptor knockouts and specific tool compounds indicate that effects on sleep are mediated by the Hcrt2 receptor^59^. A recent study has also shown that lack of either of the orexin receptors diminishes weight gain in a DIO paradigm^60^; consistent with this, we found that an Hcrtr antagonist delivered i.c.v. showed a greater effect on weight than did i.c.v. infusion of calcitonin, suggesting that an orally available agent that crossed the blood–brain barrier could have a potentially beneficial effect for the management of obesity. We then tested a novel, orally bioavailable highly specific Hcrtr1 antagonist, CVN45502. Our preclinical studies indeed show that pharmacologic antagonism of Hcrtr1 using CVN45502 reduces food intake and body weight in both DIO and ob/ob mice. This effect is consistent with the effect of chemogenetic activation of DRN^Vglut3^ neurons in ob/ob mice, indeed suggesting that CVN45502 might provide a means for ‘bypassing leptin resistance’.

CVN45502 can cross the blood–brain barrier and is known to engage both the mouse and human Hcrtr1 receptor without evident effects on a broad selectivity panel (Supplementary Table 5). Although CVN45502 shows a high affinity for Hcrtr1, similar to several previously described tool compounds, it demonstrates far superior selectivity over Hcrtr2 in biochemical and functional assays. CVN45502 has >1,000-fold selectivity for Hcrtr1 over Hcrtr2, and most importantly, at therapeutic doses (30 mg/kg), there is no binding to Hcrtr2 (Fig. 6c). In contrast, SB-334867 engages the Hcrtr2 receptor especially at doses above 20 mg/kg, as do SB-674042 and ACT-335827, and all have been reported to cause somnolence at the doses that we tested^48,59,61–63^ (Extended Data Fig. 8). The enhanced specificity of CVN45502 for Hcrt1r explains why it did not affect locomotion or time spent sleeping, whereas, in accordance with prior studies, we found that the less selective Hcrtr1 antagonist SB-334867 did. This finding is also consistent with knockout data showing that an Hcrtr1 knockout does not alter sleep, whereas Hcrtr2 knockout does^50,59^.

We also confirmed that, as was also the case in mice, Hcrtr1 is highly expressed in the human DRN, suggesting that CVN45502 could have similar effects in humans. CVN45502 had a significant effect in mice, leading to loss of 8% of their body weight; this is similar to weight loss seen with treatment with lorcaserin, an FDA-approved drug for obesity, which led to 6% weight loss in rats. The possible effect of CVN45502 in humans is difficult to predict because, although the weight-reducing effects of drugs in mice typically predict a similar direction of the response in humans, the magnitude of the effect can vary between species. Thus, although the magnitude of weight reduction in DIO mice was modest, it was highly significant, as was the effect on glucose tolerance, suggesting that CVN45502 could prove to be clinically useful in humans, although further clinical testing will be necessary to confirm this.

The approach we employed identified two other potential drug targets enriched in DRN^Vglut3^ neurons. One, GPR4, is a member of the proton-sensing GPCR family, with high expression in vascular endothelial cells, which increases following tissue injury in a model of renal ischemia and is associated with proinflammatory actions^64,65^. In the brain, GPR4 is almost uniquely expressed in the DRN region^23^. However, the ligand for GPR4 has not been identified, and it is not conclusively known whether it is G_s_-, G_q_- or G_i_-coupled. Nonetheless, identifying pharmacophores for this receptor may be possible using inverse agonist screens, although GPR4 can elicit pro-inflammatory effects of this response in the periphery, which is why we instead focused on ligands for Hcrtr1 and CalcR. The CalcR is a GPCR in the same group B subfamily that is known to engage multiple ligands (amylin, vasoactive intestinal peptide, secretin, PTH and PTHrP). It is G_s_-coupled, and it activates cAMP/PKA and PKC activity. CalcRs can also form complexes with accessory proteins called RAMPs (receptor-activity-modifying proteins) which can alter the location of the receptor and its function. Another receptor that binds to the calcitonin family of peptides is the calcitonin-like receptor (CGRP)^66^. Ligands for these receptor complexes include not just calcitonin, but also amylin, a peptide that reduces body weight in animals and humans. The amylin receptor is composed of calcitonin receptor and RAMP2 and RAMP3, whereas the CGRP receptor has a trimeric structure composed of the calcitonin-like receptor, RAMP1 and the intracellular receptor component protein (RCP). Of note, high doses of calcitonin fail to reduce food intake, although lower doses of it and CGRP, another peptide derived from the same precursor, have been shown to reduce feeding in animals^67^. The calcitonin receptor subunit is expressed in a number of brain regions that play a role to control feeding including the DMH, VMH, PVH and NTS^43,44^. Since amylin and low doses of calcitonin reduce feeding and the natural ligand for the CalcR in the DRN is unclear, it is possible that the ability of amylin, a FDA-approved drug, to reduce food intake and body weight could be mediated by effects on DRN^Vglut3^ neurons in the DRN. Of note, a recent report studying 600,000 human exomes has identified 16 genes, including *Calcr*, in which rare nonsynonymous variants were associated with body-mass index (BMI)^68^. In line with our data, truncated variants of proteins that diminish CalcR signaling were associated with higher BMI, which is consistent with our finding that a CalcR agonist (s-CT) can reduce weight (Figs. 5–6)^68^.

Although we used molecular profiling to identify potential targets for the treatment of obesity, the approach we developed to identify Hcrtr1 and CalcR as drug targets is general and could be applied to other neurologic disorders. The approach is as follows: (1) show a therapeutic effect of chemogenetic modulation of a specific cell type; (2) profile that cell type and select cell-specific transcripts by comparisons with other cell types; (3) sort cell-specific transcripts for their druggability; and (4) validate target expression in humans. This approach is facilitated by the availability of a wide array of large, and growing, databases recording the expression pattern and function of all the genes in the genome.

In summary, we provide evidence that pharmacologic modulation of the activity of DRN^Vglut3^ can be used to reduce weight. We find that DRN^Vglut3^ neurons are anatomically and functionally connected to a canonical neural circuit regulating weight and that modulates feeding downstream of the ‘block’ of leptin action in leptin resistance. Finally, these studies demonstrate the feasibility of replicating the effects of chemogenetic modulation by identifying druggable targets using a novel translational approach. This approach may thus be suitable for target identification for other circuit disorders.

## Methods

### Contact for reagent and resource sharing

Further information and requests for reagents should be directed to (and will be fulfilled by) the lead contact, Jeffrey Friedman (friedj@rockefeller.edu).

### Experimental model and subject details

All experimental approaches were approved by The Rockefeller University Institutional Animal Care and Use Committee protocol number 18066-H and were in accordance with the National Institutes of Health guidelines. Adult mice (>8 weeks old) were used for all studies. Mice were housed in a 12-hour light–dark cycle (7:00–19:00) with ad libitum access to food and water unless otherwise indicated (fasting studies and HFD studies). All lines are in a wild-type (C57BL/6J) background. Genotypes and sources for mice used in the above studies are: C57Bl6J (Jackson Laboratory 000664), Ob/ob (Jackson Laboratory 000632), Vglut3-IRES-Cre (gift from B. Lowell), Vglut2-IRES-Cre (Jackson Laboratory 016963). Male mice were used for molecular studies. Male and female mice were used for physiology studies.

#### Viral vectors

All viral vectors used in these studies have been extensively used in neuroscience. For anterograde tracing, AAV9-CAG-FLEX-GFP (Addgene, 51502) and AAV5-DIO-Synaptophysin-venus-GFP (Addgene, generated from plasmid 137188) were used. For monosynaptic rabies input circuit tracing, AAV5-Ef1A-DIO-GTB (Addgene, 27056), AAV5-Ef1A-DIO-TVA-mCherry (Addgene, generated from plasmid 37084) and EnvA-SAD-Rb-ΔG-GFP (32635, Salk Institute) were used. For chemogenetic studies, AAV5-EF1a-DIO-hM3Dq-mCherry (Addgene, 44631), AAV5-EF1a-DIO-hM4Di-mCherry (Addgene, 44632) or AAV5-EF1a-DIO-mCherry (Addgene, generated from plasmid 50462) were used. For optogenetic activation studies, AAV5-EF1a-DIO-hChR2(H134R)-EYFP (Addgene, 20298) or AAV5-EF1a-DIO-EYFP (Addgene, 27056) were used.

#### Stereotaxic surgery

Mice were anesthetized using isoflurane anesthesia, with induction at 3–4% and maintenance at 1.5–2%. Coordinates were identified using the Paxinos mouse brain atlas. For chemogenetic studies, VGLUT3-IRES-Cre mice were injected with either 0.5 µl of AAV5-EF1a-DIO-hM3Dq-mCherry, AAV5-EF1a-DIO-hM4Di-mCherry or AAV5-EF1a-DIO-mCherry in the DRN using the following coordinates relative to lambda: (0 mm ML, 0 mm AP, −2.8 mm DV). For axon terminal tracing studies, 0.5 µl AAV9-FLEX-GFP or AAV5-DIO-Synaptophysin-venus-GFP (diluted 1:10) was injected into the DRN of VGLUT3-IRES-Cre mice using the same coordinates. For optogenetic studies, mice (VGLUT3-IRES-Cre) were injected with 1.0 µl AAV5-Ef1a-DIO-ChR2(H134R)-EYFP in the DRN (coordinates, relative to lambda: +0.8 mm ML, 0 mm AP, −3.0 mm DV: 15°), followed by implantation of a fiber-optic ferrule (Thor Labs) above the LHA (coordinates, relative to Bregma: −1.0 mm ML, −1.94 mm AP, −4.75 mm DV), BLA (coordinates, relative to bregma: −1.1 mm ML, −3.2 mm AP, −5.10 mm DV), PBN (coordinates, relative to bregma: −1.45 mm ML, −5.0 mm AP, −3.0 mm DV) or LS (coordinates, relative to bregma: −0.0 mm ML, −0.58 mm AP, −3.0 mm DV). For rabies tracing studies, mice (Vglut2-IRES-Cre) were injected with AAV5-Ef1A-DIO-GTB, AAV5-Ef1A-DIO- TVA-mCherry into the LHA (coordinates, relative to bregma: −1.0 mm ML, −1.94 mm AP, −5.25 mm DV) first, and three weeks later an EnvA-SAD-Rb-ΔG-GFP was injected into the same coordinates. For pharmacology studies, wild-type mice had a cannula (Plastics One) placed in the DRN (coordinates, relative to lambda: +0.8 mm ML, 0 mm AP, −3.0 mm DV:15°) or in the third ventricle (coordinates, relative to bregma: +0.0 mm ML, −1.8 mm AP, −5.3 mm DV). For tracing, and chemogenetic studies, the skin was closed with a surgical clip. For optogenetic and pharmacology studies, the skin was closed using sutures. All DV coordinates listed are relative to the pial surface.

#### Chemogenetic studies to evaluate energy metabolism

Mice were injected with a DREADD (hM3D(Gq)), (hM4D(Gi)) or control virus in the DRN, followed by a recovery period of at least 3 weeks (see above for viruses injected). Mice were habituated with sham injections at least 5 days prior to the assay. Mice were fed a HFD for 16 weeks before CNO-mediated activation of DRN^Vglut3^ neurons began (curative), or they were given a HFD at the same time as CNO was administered to mediate activation of DRN^Vglut3^ neurons (preventive). In the curative approach, food intake and body weight were measured daily after CNO injection intraperitoneally at a dose of 1 mg/kg/day. In the preventive approach, body weight and food intake were measured on a daily basis for 11 weeks and mice received 1 mg/kg/day of CNO twice a day (7:00 a.m. and 7 p.m.). Adiposity measurements were performed using a magnetic resonance equipment (EchoMRI) system at week 1 and week 11 in the preventive setting, and at day 7 and 21 in the curative setting. Thermogenesis assays were performed in the home cage during the animal’s light phase (10 a.m.) at week 1 and week 11 in the preventive setting and at day 7 and 21 in the curative setting. Mice were given ad libitum access to chow diet or HFD during the entire experiment. iBAT temperature was measured using wireless implantable temperature probes IPTT-300 (Bio Medic Data Systems), and core body temperature was assessed using an anal probe (Braintree Scientific). Control studies were performed by injecting vehicle (saline) instead of CNO. All CNO injections were at a concentration of 1 mg/kg.

#### Chemogenetic studies to evaluate glucose metabolism

Mice were injected with a DREADD (hM3D(Gq)) or control virus in the DRN, followed by a recovery period of at least 3 weeks (see above for viruses injected). Mice were habituated with sham injections at least 5 days prior to the assay. Mice were fed a HFD for 16 weeks before CNO-mediated activation of DRN^Vglut3^ neurons began (curative), or they were given a HFD at the same time as CNO was administered to mediate activation of DRN^Vglut3^ neurons (preventive). In the curative approach, mice were fasted after 14 days of treatment and inject with a bolus of 2 g/kg of glucose for the glucose tolerance test, or were fed ad libitum and injected with a bolus of insulin at a dose of 0.75U/kg for insulin tolerance tests. In the preventive approach, after 11 weeks of CNO administration and a HFD, mice were fasted and injected with a bolus of 2 g/kg of glucose for the glucose tolerance test or were fed ad libitum and injected with a bolus of insulin at a dose of 0.75 U/kg for insulin tolerance tests. Blood glucose was assessed with a hand-held glucometer using tail blood drops at 0, 15, 30, 60 and 120 minutes after injection.

#### Multiplex fluorescence in situ hybridization

Mice were transcardially perfused with RNAse-free PBS, followed by 4% PFA. Brains were then collected and post-fixed in 4% PFA for 12–24 hours. Brains were then incubated in increasing concentrations of sucrose solution (10–30%) until precipitation. Brains were sliced with a temperature-controlled cryostat, and processed for FISH. Multiplex FISH was then performed using the RNAscope system (ACDBio). Probes for the following mRNAs were used: *Slc17a8*, *Hcrt1* and *Calcr*. Briefly, RNAscope (Advanced Cell Diagnostics) was used, as per the manufacturer’s protocol. The target probe sets used included *Gpcr4-C2*, *Calcr*-C2, *Hcrtr1*-C2 and *Slc17a8*-C3. Formalin-fixed frozen brain tissue was sectioned using a cryostat. The sections obtained were attached on Superfrost Plus Adhesion Slides (Thermo Fisher), and a hydrophobic barrier was created using Immedge Hydrophobic Barrier Pen (Vector Laboratories). Pre-treatment was done by serial submersion of the slides in 1× PBS, nuclease-free water and 100% ethanol for 2 minutes each at room temperature. Probe hybridization was achieved by incubation of 35 µL mRNA target probes for 2 hours at 40 °C using a HyBez oven. The signal was amplified by subsequent incubation of Amp-1, Amp-2 and Amp-3, one drop each for 30, 30, and 15 minutes, respectively, at 40 °C using a HyBez oven. Each incubation step was followed by two 2-minute washes using RNAscope washing buffer. Nucleic acids were stained using manufacturer’s supplied DAPI for 30 seconds, followed by two washes with 1× PBS. The slides were cover slipped and mounted using Prolong Gold Antifade Mountant (Thermo Fisher).

#### Food intake assay for optogenetic activation studies

Feeding assays were performed in the home-cage environment. A weighing container that held 3–4 pellets of chow diet was placed in one of the corners of the cage. Mice were habituated to having the weighing container in the cage. Next food intake was annotated after the three experimental sessions for 20 minutes (laser off, laser on, laser off). Optogenetic stimulation during the laser-on phase was constant and the precise frequency is defined in manuscript. Because laser stimulation in Vglut3 neurons leads to food suppression, mice were deprived of food overnight prior to studies.

#### Open field test assay for optogenetic activation studies

The OFT was used to evaluate locomotion activity after stimulation of the projections from DRN^Vglut3^ neurons to the LHA. Standard OFT arenas were used. Of note, mice had never been exposed to the OFT arena prior to studies but had been thoroughly habituated to optic fiber attachment to the lasers. Experiments were performed in three 5-minute sessions (laser off, laser on, laser off). Optogenetic stimulation during the laser-on phase was constant and the precise frequency was 10 Hz. Total distance traveled, velocity and time spent in the center versus time spent in the borders of the OFT cage (measure of anxiety) was evaluated.

#### In vivo photostimulation

For LHA cell body and or DRN-LHA terminal photostimulation studies, two types of studies were pursued: (1) Vglut3-IRES-Cre mice were injected with an AAV5-Chr2-eYFP virus in the DRN, followed by implantation of a fiber-optic cannula over the LHA; or (2) Vglut2-IRES-Cre mice were injected with an AAV5-Chr2-eYFP directly in the LHA. There were 5–6 weeks between surgeries and experiments for post-surgery recovery and to allow sufficient time for opsin expression in the terminals or cell bodies (LHA). Experimental paradigms were conducted as reported in previous work^6,7^.

#### Food-intake assay for pharmacology studies

Feeding assays were performed in the home-cage environment. Mice had ad libitum access to food prior to, during and after the experiments. Measurements of food intake were taken at 1, 2, 4, 8 and 24 hours following injection or infusion for acute studies, and every 24 hours when fed chronically on a HFD. Drug infusions were achieved by means of infusion pump (Harvard Apparatus) attach to glass syringes (Hamilton), with an infusion rate of 0.1 µl/minute. Total drug injection never exceeded 0.5 µl in local DRN studies or 2 µl in brain-wide i.c.v. infusions.

#### iDISCO^+^ whole-brain clearing and imaging

Mice were transcardially perfused with PBS, followed by 4% PFA. Brains were then put through a 24-hour post-fixing period, after which immunolabeling and whole-brain clearing were performed according to previously established protocols^21,22^. Antibodies used for GFP labeling can be found in the Key Resources Table (Supplementary Table 5). Analysis was performed using Imaris 9.1 and ClearMap^14^. For acquisition, cleared samples were imaged in a sagittal orientation (left lateral side up) on a light sheet microscope (LaVision Biotec) equipped with a sCMOS camera and LVMI-Fluor ×4 objective lens equipped with a 6-mm working distance dipping cap. Version v144 and v210 of Inspector Microscope controller software was used. Samples were scanned in the 640-nm channel. Images were taken every 6 µm and reconstructed with ClearMap software for quantification or with Imaris 9.1 software for visualization and video recording. For autofluorescence, the 480 nm channel was used with a ×1.3 objective lens.

#### Anterograde projection mapping from DRN^Vglut3^ neurons

Two approaches were taken for anterograde projection mapping as previously reported^6,7^.

#### ClearMap analysis

All quantification analyses for whole-brain studies were performed using ClearMap software (latest version available from www.idisco.info, see also ref. ^14^). Code can be found at https://github.com/ChristophKirst/ClearMap2.

#### TrailMap analysis

Light sheet images of fluorescently labeled axons were segmented using the 3D U-net-based machine-learning pipeline TRAILMAP as described in the TRAILMAP pipeline (https://github.com/AlbertPun/TRAILMAP)^26^. Updated code for our specific study is provided along with the Supplementary Information (TubeMapTrailMAp.py).

#### Immunohistochemistry

Mice were perfused transcardially with PBS followed by 4% PFA. After a 24 hour post-fixing period, brains were sectioned on a vibratome (Leica) with a section thickness of 50 µm. The following primary antibody was used: chicken anti-GFP (1:1,000, Abcam). Secondary antibody conjugated to Alexa Fluor dye (1:500, Thermo Fisher) was used. A Zeiss LSM780 confocal microscope was used for imaging brain slices.

#### RNA-seq analysis

Sequence and transcript coordinates for mouse mm10 UCSC genome and gene models were retrieved from the Bioconductor Bsgenome. Mmusculus.UCSC.mm10(version1.4.0) and TxDb. Mmusculus. UCSC. mm10. Known Gene (version 3.4.0) Bioconductor libraries, respectively. FASTQ files for the published DRN^Vglut3^ neuron TRAP experiments were obtained from the Gene Expression Omnibus (accession: GSE87890). Transcript expressions were calculated using the Salmon quantification software^69^ (version 0.8.2) and gene expression levels as TPMs and counts were retrieved using Tximport (version 1.8.0). Normalization and rlog transformation of raw read counts in genes were performed using DESeq2 (ref. ^70^) (version 1.20.0). For visualization in genome browsers, RNA-seq reads are aligned to the genome using Rsubread’s subjunct method (version 1.30.6) and exported as bigWigs normalized to reads per million using the rtracklayer package (version 1.40.6). GSVA analysis of selected genes was performed using the GSVA (version 1.34.0)^71^ R package. Visualization of genes and gene-sets as heat maps was performed using the Pheatmap R package (version 1.0.10).

#### GENSAT TRAP comparison

Gene expression enrichments of the immunoprecipitants from Vglut3 neurons were compared with expression data in the GENSAT database previously processed as described above (see ‘RNA-seq analysis’). Adjusted *P* values for the Vglut3 neurons (TRAP IP samples) versus each individual experiment in the GENSAT database were calculated with DESeq2. A combined *P* value and chi-squared statistic for all of these comparisons for each gene were then calculated using Fisher’s method, specifically with the metap R package (version 1.4)^72^. The gene list was filtered further to those that are in both the signaling receptor activity (GO:0038023) and plasma membrane (GO:0005886) Gene Ontology categories. Selected receptors for further study were validated using ISH, and the expression of these receptors compared to the GENSAT samples was also visualized using the Complex Heatmap Bioconductor R package^73^.

#### BAT gene expression

BAT of CVN45502 treated (30 mg/kg) obese and control mice was collected after 7 days of treatment. iBAT samples were collected 12 hours after the last treatment day. Total RNA was isolated from iBAT as previously described^7^.

#### Phenotyping cages

Mice were removed from their home cage and placed individually in clear glass observation cages (36 cm × 20 cm × 20 cm). Behavioral assesments were carried out in a manner similar to that described previously for dopamine receptor D1A, D2, and D3 mutants^74^ using a rapid time-sampling behavioral checklist.

The assessment cycles occurred over a 20-hour period (0–20 hours). Under these conditions, each animal was observed on one occasion only, with all assessments made by an observer who was unaware of the genotype of each animal.

#### Identification and profiling of CVN45502

CVN45502 was developed as part of a medicinal chemistry campaign emerging from a high throughput screen. The in vitro binding affinity was determined membranes derived from CHO-K1 cells stably expressing HCRTR1 or HCRTR2 with ability for CVN45502 able to displace either [^3^H]SB674042 for HCRTR1 or [^3^H]EMPA for HCRTR2 as previously described^63^. To understand the functional potency of CVN45502, the compound was incubated at a range of concentrations with Chem-1 cells stably expressing human Hcrtr1 or Hcrtr2 prior to the addition of Hcrtr (EC_80_). The ability of CVN45502 to reduce Hcrtr-evoked Ca^2+^ influx was determined using a FLIPR screening system and fluorescent calcium dye (Molecular Devices), with IC_50_ values calculated using Graphpad Prism. Mouse functional potency was determined using the same protocol but in CHO-K1 cells expressing Gα_16_ and mouse Hcrtr1. Broad selectivity of CVN45502 was determined using a commercially available panel of 125 receptors, enzymes, ion channels and transporters (Cerep, Eurofins). CVN45502 was screened at a high concentration of 10 µM with a significant interaction considered to be >50% activity. Pharmacokinetic profiles of CVN45502 were determined in C57/Bl6 mice dosed with 3 mg/kg p.o. (0.5% methylcellulose). The mice were divided into two groups for plasma and brain analysis at either 30- or 60-minutes post-dose. Samples were analyzed via LC–MS/MS and parameters calculated using Win non-lin (Certara).

#### Human tissue analysis

The expression of Hcrtr1 mRNA in raphe nuclei from human individuals was evaluated in frozen sections of midbrain from three non-diseased donors (59-year-old male, 58-year-old female and 79-year-old female donors). Post-mortem human tissues were obtained, with full consent, from Tissues for Research (UK) and the General Section of the Douglas-Bell Canada Brain Bank (Montreal, Quebec, Canada). Duplex RNAscope assays (Advanced Cell Diagnostics, Biotechne) were completed using probes to VGlut3 (c1) and Hcrtr1 (c2). On completion of the assays, sections were scanned in bright-field at ×40 (Hamamatsu Nanozoomer) and analyzed for the presence and distribution of single or double-labeled raphe neurons. Sections including locus coeruleus and a recombinant cell line expressing Hcrtr1 were used as positive controls, and sections hybridized using probes to DapB were run as negative controls.

### Immunohistochemistry

Formalin-fixed/paraffin-embedded (FFPE) sections of the human midbrain were evaluated for neuroanatomical representation of the DRN. Following heat-induced epitope retrieval in Agilent Envision plus high-pH solution in a PT Link apparatus, sections were processed immunohistochemically using an Agilent Autostainer. Primary antibodies anti-human HCRTR1 (ThermoFisher PA5-33838) were applied at 10 mg/ml and detected using Agilent Envision plus secondary antibodies and detection reagents. On completion of the assay, sections were counterstained with hematoxylin. Negative control incubations were run in parallel using identical reagents to detect the non-specific binding of rabbit IgGs in adjacent sections. The assay reagents were controlled via the immunohistochemical detection of glial fibrillary acidic protein (GFAP, Abcam ab7260) using a rabbit polyclonal antibody under identical conditions to those described for anti-HCRTR1.

#### Oral administration of compound CVN45502

CVN45502 was administered at a dose of 30 mg/kg q.d., mixed in a pellet of 0.1 g of peanut butter, to mice fed for 16 weeks in HFD until they reached DIO. DIO was confirmed through a leptin-sensitivity assay. Mice were habituated 1 week prior to the study to receive one pellet of peanut butter. All mice successfully ate the pellet for first day of habituation in less than 2 minutes. After habituation, body weight and food intake of mice were measured for 7 days in a vehicle-treatment phase in which they received only peanut butter. Next, mice were divided in aged- and weight-matched groups. The first group of mice was treated with CVN45502 and the second group of mice received only the peanut butter pellet without the CVN45502 compound. Treatment lasted 14 days to mimic DRN and ICV treatments. A lean control group was also evaluated in parallel following the same paradigm. After the 14 treatment days, mice were placed for 7 days on the peanut-butter-only paradigm to assess whether there were irreversible consequences in weight due to the chronic treatment.

#### Home-cage indirect calorimetry assessment

Energy expenditure was assessed through measurement by indirect calorimetry of VO_2_, VCO_2_, energy expenditure and locomotor activity using an automated home cage phenotyping system (TSE Systems). Mice were singly housed in a climate-controlled chamber (temperature: 22 °C; humidity: 55%; 12-hour light–12-hour dark cycle) with ad libitum access to water and chow. After 7 days (TSE Systems) of adaptation to social isolation, the mice were treated with vehicle (4 days) or CNO (6 days) (Fig. 2), or peanut butter (0.1 g) with (6 days) or without (4 days) CVN45502. Data were collected and analyzed as recommended by the manufacturers. Averaged measurements of control periods or treatment periods (CNO/CVN45502) are represented. Locomotor activity was recorded as beam breaks converted into distance/velocity, measuring activity in three dimensions. Beam breaks by mice were analyzed in the metabolic cage using custom software. The respiratory exchange ratio (RER) and energy expenditure were calculated from VO_2_ and VCO_2_ production data. Statistical assessment of the data was conducted using CalR software and ANCOVA regressions^35^.

### Statistics and reproducibility

Statistical parameters reported in the figures and figure legends are displayed as mean ± s.e.m. Significance was defined as *P* < 0.05. Significance annotations are annotated with the actual value. Mice were randomized into control or treatment groups. Control mice were age-matched littermate controls where possible. All statistics and data analysis were performed using GraphPad Prism, CalR Matlab, R or Python. For RNA-seq, transcript abundance and differential expression were performed using cufflinks.

Sample size has been included in all figure legends. Regarding replicates, the biological replicates for imaging studies (Figs. 1 and 8 and Extended Data Figs. 1, 2, 5 and 10) in cells, mice or human donors come from *n* = 3 biologically independent cells, mice or human experiments. For circuit mapping validation studies (Fig. 1 and Extended Data Fig. 1) were performed in two independent experiments asessing feeding, thermogenesis or OFT parameters. Chemogenetics and pharmacology studies in Figs. 2, 4, 5 and 7 and Extended Data Figs. 2–4, 6 and 9 were performed in two independent groups of mice exposed to a HFD (45% calories from fat Research Diets). Feeding and weight loss were measured both manually and automatically using metabolic cages (TSE). Sequencing data analyzed in Fig. 3 and Extended Data Fig. 5 come from three independent pools of three mice injected with an AAV-IV-GFP-L10 (VTRAP) following a GFP-TRAP protocol; details on the data can be found in ‘Data availability.’ Physiological properties associated with treatments with CVN45502 (Fig. 6) have been conducted in *n* = 3 biologically independent mice. Side effects for calcitonin and SB-334867 treatments presented in Extended Data Fig. 7 were conducted in *n* = 4 biologically independent mice only once owing to limited equipment accessibility. Gene expression analyses associated with CVN45502 in BAT were conducted during review to accommodate reviewers questions, and were performed in one cohort of mice per experiment (lean, 16 weeks exposure to HFD and ob/ob) given the lack of change and the number of animals (Extended Data Figs. 8 and 9). Given that treatments with CVN45502 fail to show effects in thermogenesis or indirect calorimetry, the experiment was not repeated.

### Reporting summary

Further information on research design is available in the Nature Portfolio Reporting Summary linked to this article.

## Supplementary information

Supplementary Information Synthesis of CVN45502 compound and Supplementary Tables 1–5.

Reporting Summary

Supplementary Video 1 Ascending projections from DRN^Vglut3^ neurons in whole-mount after clearing/immunostaining pipeline IDISCO^+^. Video of the 3D whole-brain projection map from DRN^Vglut3^ neuron tracing study in which Vglut3-IRES-Cre mice have been injected with an AAV9-DIO-GFP into the DRN. Staining using GFP antibody was initiated 6 weeks after viral injection, allowing for sufficient time for proper axonal projections staining.

Supplementary Software 1 Code Trailmap

## Data Availability

Sequencing datasets can be found with the accession number GSE87890 for vTRAP RNA-seq data. All *P* values have been provided in the figures. All other data (iDISCO projection mapping, ISH studies performed with RNAscope and CVN45502 materials) are available from authors on request. Details on the newly synthesized compound have been included in the Supplementary Information. Source data are provided with this paper.

## Source data

Source Data Fig. 1 Statistical Source Data
Source Data Fig. 2 Statistical Source Data
Source Data Fig. 4 Statistical Source Data
Source Data Fig. 5 Statistical Source Data
Source Data Fig. 6 Statistical Source Data
Source Data Fig. 7 Statistical Source Data
Source Data Fig. 8 Statistical Source Data
Source Data Extended Data Fig. 2 Statistical Source Data
Source Data Extended Data Fig. 3 Statistical Source Data
Source Data Extended Data Fig. 4 Statistical Source Data
Source Data Extended Data Fig. 5 Statistical Source Data
Source Data Extended Data Fig. 6 Statistical Source Data
Source Data Extended Data Fig. 7 Statistical Source Data
Source Data Extended Data Fig. 8 Statistical Source Data
Source Data Extended Data Fig. 9 Statistical Source Data
Source Data Extended Data Fig. 10 Statistical Source Data

## Code availability

GENSAT analysis found on Fig. 3 was accomplished following a custom analysis pipeline that can be found as of the date of publication at: https://github.com/RockefellerUniversity/SchneebergerPane_NatureMetabolism2022.

Code for projection mapping quantifications can be achieved using: https://github.com/ChristophKirst/ClearMap2 and https://github.com/AlbertPun/TRAILMAP. Updated code for our specific study is provided along with the Supplementary Information (TubeMapTrailMAp.py).
