# Author Correction: Pharmacological targeting of glutamatergic neurons within the brainstem for weight reduction

**DOI:** 10.1038/s42255-022-00727-1

**Published:** 2022-12-26

**Authors:** Marc Schneeberger, Nicola L. Brice, Kyle Pellegrino, Luca Parolari, Jordan T. Shaked, Keith J. Page, François Marchildon, Douglas W. Barrows, Thomas S. Carroll, Thomas Topilko, Victoria M. Mulligan, Robert Newman, Kevin Doyle, Roland Bürli, Daniel F. Barker, Angela Glen, María José Ortuño, Alexander R. Nectow, Nicolas Renier, Paul Cohen, Mark Carlton, Nathaniel Heintz, Jeffrey M. Friedman

**Affiliations:** 1 Laboratory of Molecular Genetics, Howard Hughes Medical Institute, The Rockefeller University, New York, NY USA; 2 Laboratory of Neurovascular Control of Homeostasis, Department of Cellular and Molecular Physiology, Yale School of Medicine, New Haven, CT USA; 3 Wu Tsai Institute for Brain and Cognition, Yale School of Medicine, New Haven, CT USA; 4 Cerevance, Cambridge, UK; 5 Laboratory of Molecular Metabolism, The Rockefeller University, New York, NY USA; 6 Bioinformatics Resource Center, The Rockefeller University, New York, NY USA; 7 Sorbonne Université, Paris Brain Institute, INSERM, CNRS, Hopital de la Pitié Salpétière, Paris, France; 8 Takeda Cambridge, Cambridge, UK; 9 Department of Genetics, Yale School of Medicine, New Haven, CT USA; 10 College of Physicians and Surgeons, Columbia University, New York, NY USA; 11 Laboratory of Molecular Biology, Howard Hughes Medical Institute, The Rockefeller University, New York, NY USA

**Keywords:** Pharmacogenetics, Neural circuits, Transcriptomics, Obesity

Correction to: *Nature Metabolism* 10.1038/s42255-022-00677-8. Published online 21 November 2022.

In the version of this article originally published, the surname of co-author Thomas Topilko was misspelled as Tolpiko. The error has been corrected in the HTML and PDF versions of the article.

