# Supplementary material for: Pharmacological targeting of glutamatergic neurons within the brainstem for weight reduction
Source: Nat Metab. 2022 Nov 21;4(11):1495–513. doi: 10.1038/s42255-022-00677-8 (PMC9684079; doi:10.1038/s42255-022-00677-8)
Supplement: Supplementary file 1 — Synthesis of CVN45502 compound and Supplementary Tables 1–5. [file 42255_2022_677_MOESM1_ESM.pdf]

nature metabolism

Article https://doi.org/10.1038/s42255-022-00677-8

Pharmacological targeting of glutamatergic neurons within the brainstem for weight reduction

In the format provided by the
authors and unedited

Supplementary information: Synthesis of Compound CVN45502

Introduction

The synthetic route for preparation of compound **CVN45502** is outlined in Scheme 1 and the experimental details are provided below. In brief, a nucleophilic aromatic substitution of 3-bromopyridine-2-carbonitrile (**1)** with triazole resulted in the triazole **2**, which was hydrolysed to the corresponding carboxylic acid **3**. Separately, treatment of 1-methylcyclopent-1-ene (**4)** with chloramine T provided the tosyl-aziridine **5** and subsequent *trans*-ring opening using sodium azide afforded the cyclopentane **6**, which was reduced under hydrogenation conditions to give the amine **7**. This amine was Boc-protected (→ **8**) and then deprotection of the tosyl function under *Birch* conditions resulted in amine **9**. This amine was then coupled to the carboxylic acid **3** to give amide **10** and Boc-deprotection followed by a nucleophilic aromatic substitution of the formed amine **11** with 2-chloro-5-(trifluoromethyl)pyrazine provided the desired compound in racemic form. Separation of this racemate by chiral supercritical fluid chromatography provided enantiomerically pure **CVN45502**. The final compound has been characterized by ^{1}H- and ^{19}F- NMR spectroscopy, high-resolution mass spectroscopy, analytical HPLC and chiral supercritical fluid chromatography. Its purity is above 99% with an enantiomeric excess greater than 99%. Notably, **CVN45502** is the enantiomer exhibiting potent Orexin-1 antagonist activity and its absolute configuration has been established by X-ray crystallography.

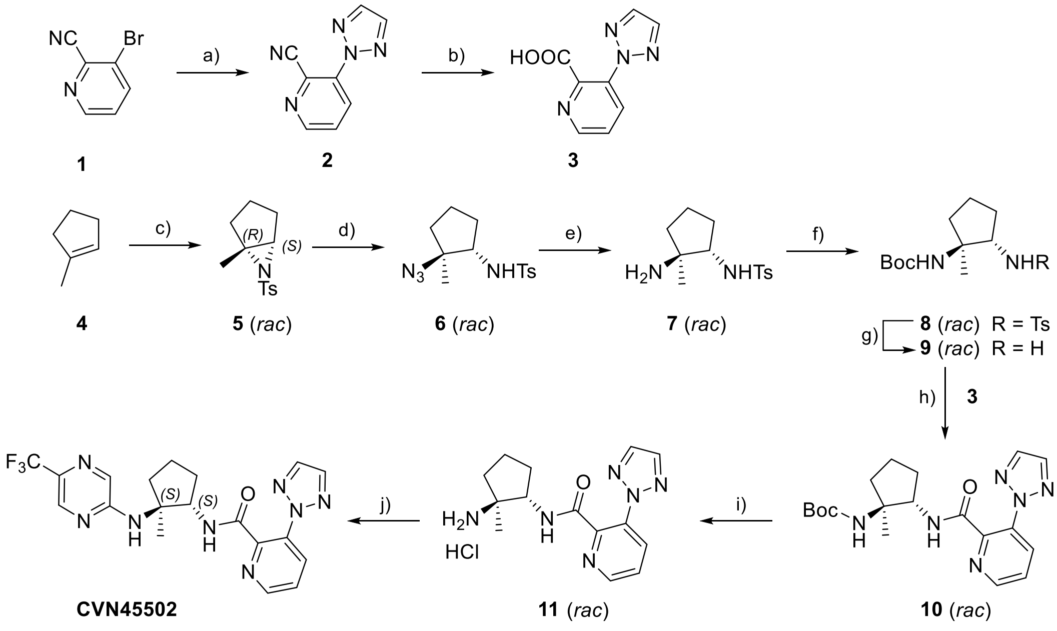

Scheme 1. Synthesis of **CVN45502**. a) triazole, Cs_2CO_3, $[CuOTf]_2C_6H_6$, DMCDA; b) aq. NaOH, EtOH; c); chloramine-T, PTAP, THF; d) NaN_3, aq. ^{i}PrOH; e) H_2, Pd-C, MeOH; f) Boc_2O, NEt_3, CH_2Cl_2; g); Li, naphthalene, THF; h) EDC·HCl, HOBt, DIEA, CH_2Cl_2; i) HCl, EtOAc; j) CTP, DIEA, DMSO, then chiral SFC separation.

General methods

Commercial reagents and solvents were used without further purification unless stated otherwise. Nuclear magnetic resonance (NMR) spectra were recorded at 300 or 400 MHz and at approximately 25 °C. The chemical shifts are reported in parts per million (ppm). Spectra were recorded using a BRUKER® AVANCE III HD (300MHz) or BRUKER AVANCE NEO (400MHz) instrument. High-resolution mass spectra were recorded on a Agilent 6230 LC/T0F instrument equipped with an ESI ion source. Analytical HPLC and LCMS were recorded on an Agilent model (1260 Infinity II) and a Shimadzu (LC20ADXR with LCMS2020) instrument, respectively. Analytical chiral supercritical fluid chromatography was performed either on a Waters instrument (model: ACQUity UPC, 4 min method) or a Shimadzu instrument (model: LC-30AD, 3 min method). Preparative chiral supercritical fluid chromatography was performed on a Waters instrument (model: prep-SFC350). The following analytical methods were applied: (a) analytical HPLC: column: Agilent HPH-C18, 4.6 * 100 mm 2.7 µm; mobile phase A: 0.05% aq. NH_3, mobile phase B: CH_3CN; Needle Wash: MeCN : H_2O = 1:1; T: 40 °C; flow rate: 1.2 mL/min; 50%-95% B 6 min, 95% B 2 min. (b) chiral SFC (4 min method): column: Cellulose-SC 100 x 4.6 mm 3.0 µm; mobile phase A: CO_2, mobile phase B: ^{i}PropOH (20 mM NH_3); flow rate: 3.0 mL/min; 10%-50% B 2.5 min, 50% B for 1.2 min, (c) chiral SFC (3 min method): column: CHIRALPACK IC-3 50 x 3.0 mm, 3.0 µm; mobile phase A: CO_2, mobile phase B: ^{i}PrOH (20 mM NH_3); flow rate: 2.0 mL/min; 10%-50% B 2.0 min, 50% B for1.0 min.

Experimental details

*3-(1,2,3-triazol-2-yl)pyridine-2-carbonitrile (**2**).* At RT under N_2 atmopshere, a solution of 3-bromopyridine-2-carbonitrile **1** (100 g, 546 mmol) and 2*H*-1,2,3-triazole (75.5 g, 1.09 mol) in DMF (1 L) was treated with Cs_2CO_3 (356 g, 1.09 mmol), copper(I) trifluoromethanesulfonate benzene complex (55.0 g, 109 mmol) and (1*S*,2*S*)-*N*1,*N*2-dimethylcyclohexane-1,2-diamine (3.89 g, 27.3 mmol). The resulting mixture was stirred for 2 h at 120 °C under N_2, cooled to , and diluted with H_2O (2 L). The resulting mixture was extracted with EtOAc (2 x 1 L). The combined organic layers were washed with brine (1 x 1 L), dried over Na_2SO_4 and concentrated under reduced pressure. The residue was purified by silica gel column chromatography (petrol ether/EtOAc 2:1) to afford 3-(1,2,3-triazol-2-yl)pyridine-2-carbonitrile (50 g, 53%) as a white solid. ^{1}H-NMR (400 MHz, DMSO-*d*$_6$): δ 8.84 (dd, *J* = 4.6, 1.5 Hz, 1H), 8.59 (dd, *J* = 8.6, 1.5 Hz, 1H), 8.37 (s, 2H), 7.99 (dd, *J* = 8.5, 4.6 Hz, 1H). MS (ESI): m/z 172.1 $[M + H]^+$.

*3-(1,2,3-triazol-2-yl)pyridine-2-carboxylic acid (**3**).* A RT, a solution of 3-(1,2,3-triazol-2-yl)pyridine-2-carbonitrile (50 g, 292 mmol) in EtOH (500 mL) was treated with 1M aq. NaOH solution (48 mL) and stirred for 16 h at 100 °C. The resulting mixture was concentrated under reduced pressure, cooled to RT and acidified to pH 3 with conc. HCl. The resulting mixture was extracted with CH_2Cl_2 (6 x 500 mL), dried over Na_2SO_4 and concentrated under reduced pressure, resulting in 3-(1,2,3-triazol-2-yl)pyridine-2-carboxylic acid (22.5 g, 41%) as a white solid. ^{1}H-NMR (400 MHz, DMSO-*d*$_6$): δ 12.95 (s, 1H), 8.69 (dd, *J* = 4.7, 1.4 Hz, 1H), 8.34 (dd, *J* = 8.3, 1.4 Hz, 1H), 8.18 (s, 2H), 7.74 (dd, *J* = 8.3, 4.7 Hz, 1H). MS (ESI): m/z 191.0 [M

+ H]$^+$. *1-methyl-6-(4-methylbenzenesulfonyl)-6-azabicyclo[3.1.0]hexane (**5**).* At RT under N_2 atmosphere, a stirred solution of 1-methylcyclopent-1-ene **4** (100 g, 1217 mmol) and chloramine T (388 g, 1.70 mol) in THF (2 L) was treated with PTAP (45.8 g, 122 mmol) and stirred for overnight. The mixture was poured into H_2O (3 L) and extracted with EtOAc (3 x 1.5 L). The combined organic layers were washed with brine (1 x 1 L), dried over Na_2SO_4 and concentrated under reduced pressure. The residue was purified by silica gel column chromatography (petrol ether/EtOAc 10:1) to afford 1-methyl-6-(4-methylbenzenesulfonyl)-6-azabicyclo[3.1.0]hexane (100 g, 33%) as a light yellow solid. ^{1}H-NMR (400 MHz, DMSO-d_6): δ 7.76 (d, J = 8.3 Hz, 2H), 7.41 (d, J = 8.1 Hz, 2H), 3.35 (s, 1H), 2.40 (s, 3H), 1.91 (dd, J = 13.3, 8.1 Hz, 1H), 1.75 (s, 3H), 1.70-1.62 (m, 2H), 1.62-1.42 (m, 2H), 1.28-1.11 (m, 1H). MS (ESI): m/z 252.2 [M + H]$^+$. *N-[(1S,2S)-2-azido-2-methylcyclopentyl]-4-methylbenzenesulfonamide (**6**).* A stirred solution of 1-methyl-6-(4-methylbenzenesulfonyl)-6-azabicyclo[3.1.0]hexane **5** (100 g, 398 mmol) in ^{i}PrOH (2 L) and H_2O (2 L) was treated at RT with NaN_3 (103.5 g, 1.59 mol) and stirred overnight. The mixture was diluted with H_2O (1 L) and extracted with MTBE (3 x 1.5 L). The combined organic layers were washed with brine (1 x 2 L), dried over Na_2SO_4 and concentrated under reduced pressure to afford *N*-[(1*S*,2*S*)-2-azido-2-methylcyclopentyl]-4-methylbenzenesulfonamide (120 g, crude) as a white solid, which was used in the next step without further purification. ^{1}H-NMR (400 MHz, DMSO-d_6): δ 7.83-7.64 (m, 3H), 7.39 (d, J = 8.0 Hz, 2H), 3.37 (q, J = 8.2 Hz, 1H), 2.39 (s, 3H), 1.69-1.39 (m, 5H), 1.32-1.16 (m, 4H). MS (ESI): m/z 267.2 [M – N_2 + H]$^+$. *N-[(1S,2S)-2-amino-2-methylcyclopentyl]-4-methylbenzenesulfonamide (7).* At RT under N_2 atmosphere, a solution of *N*-[(1*S*,2*S*)-2-azido-2-methylcyclopentyl]-4-methylbenzenesulfonamide **6** (120 g, 407 mmol) in MeOH (2.4 L) was treated with Pd/C (10%, 24 g). The resulting mixture was stirred at RT under H_2 atmosphere (1 atm) overnight, filtered through *Celite* and concentrated under reduced pressure to afford *N*-[(1*S*,2*S*)-2-amino-2-methylcyclopentyl]-4-methylbenzenesulfonamide **7** (64 g, 59%) as a colorless oil. The crude product was used in the next step without further purification. ^{1}H-NMR (400 MHz, DMSO-d_6): δ 7.71 (d, J = 8.2 Hz, 2H), 7.38 (d, J = 7.9 Hz, 2H), 2.99 (t, J = 8.3 Hz, 1H), 2.39 (s, 3H), 1.68-1.33 (m, 5H), 1.28-1.17 (m, 1H), 0.94 (s, 3H). MS (ESI): m/z 269.1 [M + H]$^+$.*tert-Butyl N-[(1S,2S)-1-methyl-2-(4-methylbenzenesulfonamido)cyclopentyl]carbamate (**8**).* At RT, a stirred solution of *N*-[(1*S*,2*S*)-2-amino-2-methylcyclopentyl]-4-methylbenzenesulfonamide **7** (64 g, 238 mmol) and NEt_3 (36.2 g, 358 mmol) in CH_2Cl_2 (1 L) was dropwise treated with Boc_2O (62.5 g, 286 mmol) in CH_2Cl_2 (0.3 L). The resulting mixture was stirred at RT for 5 h and concentrated under reduced pressure. The residue was purified by silica gel column chromatography (petrol ether/EtOAc 10:1) to afford *tert*-butyl *N*-[(1*S*,2*S*)-1-methyl-2-(4-methylbenzenesulfonamido)cyclopentyl]carbamate (69.5 g, 80%) as a white solid.

^{1}H-NMR (400 MHz, DMSO-d_6): δ 7.68 (d, J = 8.0 Hz, 2H), 7.60 (d, J = 8.2 Hz, 1H), 7.39 (d, J = 7.9 Hz, 2H), 6.22 (s, 1H), 3.78-3.57 (m, 1H), 2.39 (s, 3H), 2.05-1.82 (m, 1H), 1.66-1.51 (m, 1H), 1.52-1.31 (m, 12H), 1.30-1.18 (m, 1H), 1.09 (s, 3H). MS (ESI): m/z 313.1 [M-^{t}Butyl]$^+$.*tert-Butyl N-[(1S,2S)-2-amino-1-methylcyclopentyl]carbamate (**9**).* At 0 °C under argon atmosphere, a stirred solution of naphthalene (69.9 g, 545 mmol) in THF (2.3 L) was treated portion-wise with Li (10.1 g, 1454 mmol). The resulting mixture was stirred for 3 h at 0 °C under argon atmosphere and dropwise treated with a solution of *tert*-butyl *N*-[(1*S*,2*S*)-1-methyl-2-(4-methylbenzenesulfonamido)cyclopentyl]carbamate **8** (67 g, 218 mmol) in THF

(0.4 L). The mixture was stirred for 3 h at 0 °C, quenched with sat. aq. NH_4Cl (2 L) and acidified to pH 2-3 using 1M aq. HCl at 0 °C. The resulting mixture was extracted with MTBE (2 x 1 L). The combined aqueous layer was basified to pH 14 with 10% aq. NaOH solution. The resulting mixture was extracted with CH_2Cl_2 (2 x 1 L). The combined organic layers were washed with brine (1 x 1 L), dried over Na_2SO_4 and concentrated under reduced pressure to afford *tert*-butyl *N*-[(1*S*,2*S*)-2-amino-1-methylcyclopentyl]carbamate **9** (18.6 g, 48%) as a yellow solid. ^{1}H-NMR (300 MHz, DMSO-*d*$_6$): δ 6.50 (s, 1H), 3.03 (t, *J* = 8.5 Hz, 1H), 1.92–1.68 (m, 3H), 1.58-1.42 (m, 2H), 1.41-1.32 (m, 11H), 1.26-1.12 (m, 1H), 1.06 (s, 3H). MS (ESI): m/z 215.1 [M + H]$^+$.*tert-Butyl N-[(1S,2S)-1-methyl-2-[3-(1,2,3-triazol-2-yl)pyridine-2-amido]cyclopentyl]carbamate (**10**)*. At RT, a solution of *tert*-butyl *N*-[(1*S*,2*S*)-2-amino-1-methylcyclopentyl]carbamate (18.6 g, 86.8 mmol), 3-(1,2,3-triazol-2-yl)pyridine-2-carboxylic acid (17.3 g, 91.1 mmol), DIEA (22.4 g, 173.5 mmol) and EDC·HCl (20.2 g, 130 mmol) in CH_2Cl_2 (370 mL) was treated with HOBT (17.6 g, 130.2 mmol) and stirred overnight. The resulting mixture was diluted with H_2O (370 mL) and extracted with CH_2Cl_2 (2 x 150 mL). The combined organic layers were washed with brine (1 x 200 mL), dried over Na_2SO_4 and concentrated under reduced pressure. The residue was purified by silica gel column chromatography, (petrol ether/EtOAc 5:1) to afford *tert*-butyl *N*-[(1*S*,2*S*)-1-methyl-2-[3-(1,2,3-triazol-2-yl)pyridine-2-amido]cyclopentyl]carbamate (23.5 g, 70%) as a white solid. ^{1}H-NMR (400 MHz, DMSO-*d*$_6$): δ 8.80-8.66 (m, 2H), 8.31 (dd, *J* = 8.2, 1.4 Hz, 1H), 8.09 (s, 2H), 7.74 (dd, *J* = 8.2, 4.7 Hz, 1H), 6.55 (s, 1H),4.29 (q, *J* = 8.0 Hz, 1H), 2.10-1.94 (m, 1H), 1.92-1.81 (m, 2H), 1.71-1.55 (m, 3H), 1.38 (s, 9H), 1.20 (s, 3H). MS (ESI): m/z 387.1 [M + H]$^+$.

*N-[(1S,2S)-2-amino-2-methylcyclopentyl]-3-(1,2,3-triazol-2-yl)pyridine-2-carboxamide hydrochloride (**11**)*. At RT, a solution of *tert*-butyl *N*-[(1*S*,2*S*)-1-methyl-2-[3-(1,2,3-triazol-2-yl)pyridine-2-amido]cyclopentyl]carbamate **10** (23.5 g, 60.8 mmol) in EtOAc (235 mL) saturated with HCl (g) was stirred for 3 h. The precipitated solids were collected by filtration, washed with EtOAc (2 x 50 mL) and dried *in vacuo*, resulting in *N*-[(1*S*,2*S*)-2-amino-2-methylcyclopentyl]-3-(1,2,3-triazol-2-yl)pyridine-2-carboxamide hydrochloride **11** (15.5 g, 79%) as a white solid. ^{1}H-NMR (400 MHz, DMSO-*d*$_6$): δ 9.05-8.83 (m, 2H), 8.73 (dd, *J* = 4.7, 1.4 Hz, 1H), 8.36-8.29 (m, 1H), 8.23 (s, 2H), 8.16 (s, 2H), 7.77 (dd, *J* = 8.3, 4.7 Hz, 1H), 4.39-4.17 (m, 1H), 2.19-1.94 (m, 2H), 1.85-1.59 (m, 4H), 1.29 (s, 3H). MS (ESI): m/z 287.1 [M + H]$^+$.*N-[(1S,2S)-2-methyl-2-{[5-(trifluoromethyl)pyrazin-2-yl]amino}cyclopentyl]-3-(1,2,3-triazol-2-yl)pyridine-2-carboxamide (**CVN45502**)*. At RT under N_2 atmosphere, a stirred solution of *N*-[(1*S*,2*S*)-2-amino-2-methylcyclopentyl]-3-(1,2,3-triazol-2-yl)pyridine-2-carboxamide hydrochloride **11** (15.5 g, 48.0 mmol) and 2-chloro-5-(trifluoromethyl)pyrazine (8.76 g, 48.0 mmol) in DMSO (310 mL) was treated with DIEA (31.0 g, 240 mmol) and stirred for 3 h at 140 °C. The mixture was allowed to cool to RT and poured into ice/water (620 mL). The precipitated solids were collected by filtration and washed with H_2O (2 x 20 mL). The solids were purified by trituration with MTBE (50 mL) and collected by filtration. The resulting solids were dissolved with CH_2Cl_2 (500 mL) and decolorized by addition of activated carbon (30 g). The mixture was filtered and the filtrate concentrated and purified by chiral SFC (CHIRAL ART Cellulose-SC, 5*25 cm, 5 μm; mobile phase A: CO_2, mobile phase B: ^{i}PrOH; isocratic 50% B), resulting in *N*-[(1*S*,2*S*)-2-methyl-2-{[5-(trifluoromethyl)pyrazin-2-yl]amino}cyclopentyl]-3-(1,2,3-triazol-2-yl)pyridine-2-carboxamide **CVN45502** (isomer 1,

retention time 4.01 min) (5.23 g, 25%) as a white solid. ^{1}H-NMR (300 MHz, CD_2Cl_2): δ 8.71 (dd, *J* = 4.7, 1.5 Hz, 1H), 8.27 (s, 1H), 8.14 (dd, *J* = 8.2, 1.5 Hz, 1H), 7.87 (s, 2H), 7.77-7.63 (m, 2H), 7.58-7.41 (m, 2H), 4.63-4.43 (m, 1H), 2.78-2.52 (m, 1H), 2.39-2.10 (m, 1H), 2.09-1.64 (m, 4H), 1.46 (s, 3H). ^{19}F-NMR (282 MHz, CD_2Cl_2) δ -66.64. HR-MS (ESI) for $C_{19}H_{20}F_3N_8O$: 433.128 (calc.), 433.1698 (found). Analyt. HPLC: 99.6%. Chiral SFC: 99.5%.

Abbreviations

CTP	2-Chloro-5-(trifluoromethyl)pyrazine
DIEA	Diisopropylethylamine
DMCDA	(1*S*,2*S*)-*N*1,*N*2-dimethylcyclohexane-1,2-diamine
EDC	*N*-Ethyl-*N'*-(3-dimethylaminopropyl)carbodiimide
PTAP	Phenyltrimethylammonium tribromide
RT	Room temperature
SFC	Supercritical fluid chromatography

Crystallography

The absolute configuration of **CVN45502** was unambiguously determined by x-ray crystallography (Figure 1).

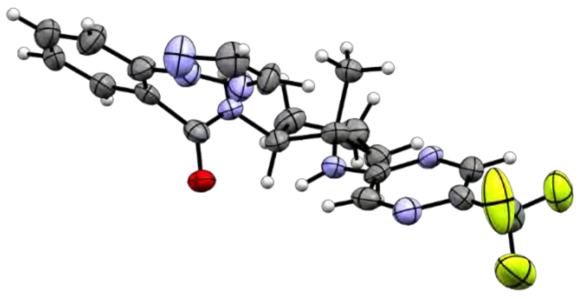

Figure 1. Solid-state structure of **CVN45502**.

The key data of the crystallography experiment is summarized below.

- audit_creation_method 'by CrystalStructure 4.1'
- References
  Rigaku (2014). CrystalStructure. Version 4.1. Rigaku Corporation, Tokyo, Japan.
  Burla, M. C., Caliandro, R., Camalli, M., Carrozzini, B., Cascarano, G. L., De Caro, L., Giacovazzo, C., Polidori, G., Siliqi, D. and Spagna R. (2007). J. Appl. Cryst. 40, 609-613.

CHEMICAL DATA

Chemical formula	$C_{20}H_{20}F_3N_7O$
Chemical formula weight	431.42
Space group crystal system	monoclinic
Space group name H-M alt	'P 1 21 1'
Space group name Hall	'P 2yb'
Space group IT number	4

loop__symmetry_equiv_pos_site_id_space_group_symop_operation_xyz
1 '+X,+Y,+Z'
2 '-X,1/2+Y,-Z'

_cell_length_a	11.4356(2)
_cell_length_b	8.85909(16)
_cell_length_c	24.1588(4)
_cell_angle_alpha	90.0000
_cell_angle_beta	96.697(7)
_cell_angle_gamma	90.0000
_cell_volume	2430.81(8)
_cell_formula_units_Z	4
_cell_measurement_reflns_used	59310
_cell_measurement_theta_min	3.69
_cell_measurement_theta_max	68.25
_cell_measurement_temperature	100

_exptl_crystal_description	platelet
_exptl_crystal_colour	colorless
_exptl_crystal_size_max	0.176
_exptl_crystal_size_mid	0.142
_exptl_crystal_size_min	0.050
_exptl_crystal_density_diffrn	1.179
_exptl_crystal_density_meas	?
_exptl_crystal_density_method	'not measured'
_exptl_crystal_F_000	896.00
_exptl_absorpt_coefficient_mu	0.794
_exptl_absorpt_correction_type	multi-scan
_exptl_absorpt_process_details	'ABSCOR (Rigaku, 1995)'
_exptl_absorpt_correction_T_min	0.692
_exptl_absorpt_correction_T_max	0.961

EXPERIMENTAL DATA

_diffrn_ambient_temperature	100
_diffrn_radiation_type	'Cu K\a'
_diffrn_radiation_wavelength	1.54187
_diffrn_measurement_device_type	'Rigaku R-AXIS RAPID 191R'
_diffrn_measurement_method	\w
_diffrn_detector_area_resol_mean	10.000
_diffrn_reflns_number	104116

_diffrn_reflns_av_R_equivalents	0.0479
_diffrn_reflns_theta_max	68.197
_diffrn_reflns_theta_min	3.684
_diffrn_reflns_theta_full	67.687
_diffrn_measured_fraction_theta_max	1.000
_diffrn_measured_fraction_theta_full	1.000
_diffrn_reflns_Laue_measured_fraction_max	1.000
_diffrn_reflns_Laue_measured_fraction_full	1.000
_diffrn_reflns_point_group_measured_fraction_max	1.000
_diffrn_reflns_point_group_measured_fraction_full	1.000
_diffrn_reflns_limit_h_min	-13
_diffrn_reflns_limit_h_max	13
_diffrn_reflns_limit_k_min	-10
_diffrn_reflns_limit_k_max	10
_diffrn_reflns_limit_l_min	-29
_diffrn_reflns_limit_l_max	29
_diffrn_standards_number	0

REFINEMENT DATA

_refine_special_details

Refinement was performed using all reflections. The weighted
R-factor (wR) and goodness of fit (S) are based on F^2^.
R-factor (gt) are based on F. The threshold expression of
F^2^ > 2.0 sigma(F^2^) is used only for calculating R-factor (gt).

_reflns_number_total	8915
_reflns_number_gt	8803
_reflns_threshold_expression	F^2^>2.0\s(F^2^)
_refine_ls_structure_factor_coef	Fsqd
_refine_ls_R_factor_all	0.0823
_refine_ls_R_factor_gt	0.0815
_refine_ls_wR_factor_ref	0.1926
_refine_ls_wR_factor_gt	0.1921
_refine_ls_number_restraints	5
_refine_ls_hydrogen_treatment	constr
_refine_ls_number_reflns	8915
_refine_ls_number_parameters	515
_refine_ls_goodness_of_fit_ref	1.247
_refine_ls_weighting_scheme	calc

_refine_ls_weighting_details
'w = 1/[\s^2^(Fo^2^)+(0.0709P)^2^+1.7543P] where P=(Fo^2^+2Fc^2^)/3'

_atom_sites_solution_hydrogens	geom
_atom_sites_solution_primary	direct
_atom_sites_solution_secondary	difmap
_refine_ls_shift/su_max	0.008
_refine_diff_density_max	0.32
_refine_diff_density_min	-0.30

_refine_ls_extinction_method none
_refine_ls_extinction_coef ?
_refine_ls_abs_structure_details
;
 Flack x determined using 3743 quotients [(I+)-(I-)]/[(I+)+(I-)]
 (Parsons and Flack (2004), Acta Cryst. A60, s61).
;
_refine_ls_abs_structure_Flack 0.03(4)

loop_
_atom_type_symbol
_atom_type_description
_atom_type_scat_dispersion_real
_atom_type_scat_dispersion_imag
_atom_type_scat_source
 'C' 'C' 0.0181 0.0091
International Tables for Crystallography
(Vol. C, Tables 4.2.6.8 and 6.1.1.4)
 'H' 'H' 0.0000 0.0000
International Tables for Crystallography
(Vol. C, Table 6.1.1.4)
 'F' 'F' 0.0727 0.0534
International Tables for Crystallography
(Vol. C, Tables 4.2.6.8 and 6.1.1.4)
 'N' 'N' 0.0311 0.0180
International Tables for Crystallography
(Vol. C, Tables 4.2.6.8 and 6.1.1.4)
 'O' 'O' 0.0492 0.0322
International Tables for Crystallography
(Vol. C, Tables 4.2.6.8 and 6.1.1.4)

ATOMIC COORDINATES AND THERMAL PARAMETERS

loop_
_atom_site_label
_atom_site_type_symbol
_atom_site_fract_x
_atom_site_fract_y
_atom_site_fract_z
_atom_site_U_iso_or_equiv
_atom_site_adp_type
_atom_site_occupancy
_atom_site_site_symmetry_multiplicity
_atom_site_calc_flag
_atom_site_refinement_flags
_atom_site_disorder_assembly
_atom_site_disorder_group
 N1A N 0.7494(11) 0.7117(14) 0.2857(4) 0.0495(13) Uani 0.5 2 d . . .

N2A N 0.8309(10) 0.7909(12) 0.3187(5) 0.0495(13) Uani 0.5 2 d . . .
C3A C 0.7982(12) 0.7545(14) 0.3675(6) 0.0495(13) Uani 0.5 2 d . . .
C4A C 0.6969(12) 0.6570(16) 0.3632(6) 0.0495(13) Uani 0.5 2 d . . .
N5A N 0.6716(10) 0.6330(14) 0.3097(5) 0.0495(13) Uani 0.5 2 d . . .
C6A C 0.7490(10) 0.708(2) 0.2266(3) 0.0495(13) Uani 0.5 2 d . . .
C7A C 0.6454(7) 0.710(2) 0.1902(5) 0.0495(13) Uani 0.5 2 d . . .
C8A C 0.6501(5) 0.710(2) 0.1330(5) 0.0495(13) Uani 0.5 2 d . . .
C9A C 0.7584(7) 0.7079(16) 0.1121(3) 0.0495(13) Uani 0.5 2 d . . .
C10A C 0.8619(5) 0.7057(14) 0.1485(4) 0.0495(13) Uani 0.5 2 d . . .
C11A C 0.8572(7) 0.7055(16) 0.2057(3) 0.0495(13) Uani 0.5 2 d . . .
N1D N 0.7661(13) 0.6943(14) 0.2767(4) 0.0585(15) Uani 0.5 2 d . . .
N2D N 0.8662(11) 0.7504(12) 0.3050(6) 0.0585(15) Uani 0.5 2 d . . .
C3D C 0.8434(13) 0.7167(15) 0.3575(7) 0.0585(15) Uani 0.5 2 d . . .
C4D C 0.7346(13) 0.6417(16) 0.3573(6) 0.0585(15) Uani 0.5 2 d . . .
N5D N 0.6952(11) 0.6165(14) 0.3051(6) 0.0585(15) Uani 0.5 2 d . . .
C6D C 0.7540(11) 0.706(2) 0.2173(4) 0.0585(15) Uani 0.5 2 d . . .
C7D C 0.6425(9) 0.712(3) 0.1876(6) 0.0585(15) Uani 0.5 2 d . . .
C8D C 0.6303(6) 0.715(2) 0.1296(6) 0.0585(15) Uani 0.5 2 d . . .
C9D C 0.7296(8) 0.7114(17) 0.1015(4) 0.0585(15) Uani 0.5 2 d . . .
C10D C 0.8411(6) 0.7054(15) 0.1313(4) 0.0585(15) Uani 0.5 2 d . . .
C11D C 0.8533(7) 0.7027(17) 0.1892(4) 0.0585(15) Uani 0.5 2 d . . .
C12A C 0.5326(5) 0.7368(6) 0.2151(2) 0.0417(13) Uani 1 2 d . . .
O13A O 0.5183(4) 0.8517(4) 0.24263(17) 0.0470(10) Uani 1 2 d . . .
N14A N 0.4482(4) 0.6312(5) 0.20365(18) 0.0410(11) Uani 1 2 d . . .
C15A C 0.3320(5) 0.6426(6) 0.2224(3) 0.0437(13) Uani 1 2 d . . .
C16A C 0.3271(5) 0.5948(6) 0.2838(3) 0.0428(13) Uani 1 2 d . . .
C17A C 0.1928(6) 0.5707(8) 0.2835(3) 0.0570(16) Uani 1 2 d . . .
C18A C 0.1546(6) 0.4910(8) 0.2290(3) 0.0587(17) Uani 1 2 d . . .
C19A C 0.2400(5) 0.5449(7) 0.1878(3) 0.0489(14) Uani 1 2 d . . .
C20A C 0.3972(6) 0.4473(7) 0.2973(3) 0.0524(16) Uani 1 2 d . . .
N21A N 0.3751(5) 0.7165(6) 0.32054(18) 0.0481(12) Uani 1 2 d . . .
C22A C 0.3864(6) 0.7159(8) 0.3754(3) 0.0523(15) Uani 1 2 d . . .
N23A N 0.3337(6) 0.6147(8) 0.4039(2) 0.0648(16) Uani 1 2 d . . .
C24A C 0.3496(8) 0.6237(10) 0.4599(3) 0.074(2) Uani 1 2 d . . .
C25A C 0.4182(8) 0.7323(9) 0.4876(3) 0.071(2) Uani 1 2 d . . .
N26A N 0.4730(6) 0.8371(8) 0.4598(3) 0.0742(18) Uani 1 2 d . . .
C27A C 0.4563(7) 0.8288(9) 0.4051(3) 0.0652(19) Uani 1 2 d . . .
C28A C 0.4380(11) 0.7446(11) 0.5490(4) 0.090(3) Uani 1 2 d . . .
F29A F 0.3751(7) 0.6383(8) 0.5733(2) 0.120(2) Uani 1 2 d . . .
F30A F 0.5475(7) 0.7241(8) 0.5704(2) 0.125(2) Uani 1 2 d . . .
F31A F 0.4048(8) 0.8784(7) 0.5676(2) 0.136(3) Uani 1 2 d . . .
N1K N 0.4538(4) 0.0412(6) 0.0646(2) 0.0452(11) Uani 1 2 d . . .
N2K N 0.4329(5) -0.0099(9) 0.0129(2) 0.0690(18) Uani 1 2 d . . .
C3K C 0.5388(7) -0.0030(9) -0.0050(3) 0.0615(18) Uani 1 2 d . . .
C4K C 0.6168(5) 0.0558(8) 0.0359(3) 0.0506(15) Uani 1 2 d . . .
N5K N 0.5643(4) 0.0822(6) 0.0808(2) 0.0519(13) Uani 1 2 d . . .
C6K C 0.3653(5) 0.0359(7) 0.1023(3) 0.0438(13) Uani 1 2 d . . .
C7K C 0.3823(5) 0.1113(6) 0.1530(3) 0.0419(13) Uani 1 2 d . . .

C8K C 0.2961(5) 0.0942(7) 0.1898(3) 0.0480(14) Uani 1 2 d . . .
C9K C 0.1952(5) 0.0159(7) 0.1743(3) 0.0487(15) Uani 1 2 d . . .
C10K C 0.1766(6) -0.0541(8) 0.1229(3) 0.0576(17) Uani 1 2 d . . .
C11K C 0.2623(6) -0.0435(8) 0.0872(3) 0.0600(17) Uani 1 2 d . . .
C12K C 0.4844(5) 0.2149(6) 0.1716(2) 0.0376(11) Uani 1 2 d . . .
O13K O 0.4904(3) 0.3418(4) 0.14957(16) 0.0409(9) Uani 1 2 d . . .
N14K N 0.5590(4) 0.1645(5) 0.21363(19) 0.0387(10) Uani 1 2 d . . .
C15K C 0.6575(5) 0.2532(6) 0.2400(2) 0.0405(13) Uani 1 2 d . . .
C16K C 0.7729(5) 0.2314(6) 0.2132(2) 0.0424(13) Uani 1 2 d . . .
C17K C 0.8640(5) 0.3043(7) 0.2583(2) 0.0449(14) Uani 1 2 d . . .
C18K C 0.8213(6) 0.2511(8) 0.3137(3) 0.0536(16) Uani 1 2 d . . .
C19K C 0.6913(5) 0.2133(8) 0.3008(2) 0.0480(14) Uani 1 2 d . . .
C20K C 0.8022(5) 0.0661(6) 0.2052(3) 0.0413(13) Uani 1 2 d . . .
N21K N 0.7625(4) 0.3111(5) 0.1597(2) 0.0408(11) Uani 1 2 d . . .
C22K C 0.8466(5) 0.3229(6) 0.1258(3) 0.0430(13) Uani 1 2 d . . .
N23K N 0.9579(4) 0.2852(5) 0.14360(19) 0.0382(10) Uani 1 2 d . . .
C24K C 1.0375(5) 0.3126(7) 0.1080(3) 0.0452(14) Uani 1 2 d . . .
C25K C 1.0100(5) 0.3773(7) 0.0573(3) 0.0438(13) Uani 1 2 d . . .
N26K N 0.8958(5) 0.4078(6) 0.0364(2) 0.0458(11) Uani 1 2 d . . .
C27K C 0.8174(6) 0.3792(6) 0.0694(2) 0.0446(14) Uani 1 2 d . . .
C28K C 1.0991(7) 0.4155(10) 0.0195(3) 0.069(2) Uani 1 2 d . . .
F29K F 1.2056(4) 0.3609(6) 0.03793(18) 0.0776(13) Uani 1 2 d . . .
F30K F 1.0741(4) 0.3723(10) -0.03119(19) 0.123(3) Uani 1 2 d . . .
F31K F 1.1128(5) 0.5668(7) 0.0164(3) 0.118(2) Uani 1 2 d . . .
H3A H 0.83742 0.78922 0.40194 0.0595 Uiso 0.5 2 calc R . .
H4A H 0.65724 0.61856 0.39263 0.0595 Uiso 0.5 2 calc R . .
H8A H 0.57937 0.71156 0.10812 0.0595 Uiso 0.5 2 calc R . .
H9A H 0.76161 0.70811 0.07302 0.0595 Uiso 0.5 2 calc R . .
H10A H 0.93592 0.70428 0.13426 0.0595 Uiso 0.5 2 calc R . .
H11A H 0.92799 0.70391 0.23061 0.0595 Uiso 0.5 2 calc R . .
H3D H 0.89434 0.7406 0.39026 0.0702 Uiso 0.5 2 calc R . .
H4D H 0.69714 0.61468 0.38898 0.0702 Uiso 0.5 2 calc R . .
H8D H 0.55409 0.71868 0.10929 0.0702 Uiso 0.5 2 calc R . .
H9D H 0.72125 0.7132 0.06193 0.0702 Uiso 0.5 2 calc R . .
H10D H 0.90894 0.70318 0.11207 0.0702 Uiso 0.5 2 calc R . .
H11D H 0.92947 0.69864 0.20957 0.0702 Uiso 0.5 2 calc R . .
H14A H 0.46412 0.5518 0.18395 0.0492 Uiso 1 2 calc R . .
H15A H 0.30599 0.75022 0.21846 0.0525 Uiso 1 2 calc R . .
H17A H 0.17606 0.50792 0.31551 0.0684 Uiso 1 2 calc R . .
H17B H 0.15167 0.66864 0.28519 0.0684 Uiso 1 2 calc R . .
H18A H 0.07241 0.51799 0.21503 0.0704 Uiso 1 2 calc R . .
H18B H 0.15964 0.38019 0.23397 0.0704 Uiso 1 2 calc R . .
H19A H 0.278 0.45758 0.17153 0.0587 Uiso 1 2 calc R . .
H19B H 0.19744 0.60458 0.15714 0.0587 Uiso 1 2 calc R . .
H20A H 0.38047 0.40841 0.33349 0.0628 Uiso 1 2 calc R . .
H20B H 0.48166 0.46793 0.29847 0.0628 Uiso 1 2 calc R . .
H20C H 0.37389 0.37211 0.26831 0.0628 Uiso 1 2 calc R . .
H21A H 0.39875 0.79815 0.30437 0.0577 Uiso 1 2 calc R . .

H24A H 0.31165 0.55186 0.48093 0.0894 Uiso 1 2 calc R . .
H27A H 0.49304 0.90232 0.38432 0.0782 Uiso 1 2 calc R . .
H3K H 0.55668 -0.03431 -0.04066 0.0737 Uiso 1 2 calc R . .
H4K H 0.6974 0.07529 0.03269 0.0608 Uiso 1 2 calc R . .
H8K H 0.30863 0.13799 0.22587 0.0576 Uiso 1 2 calc R . .
H9K H 0.13689 0.00925 0.19918 0.0585 Uiso 1 2 calc R . .
H10K H 0.10601 -0.10861 0.11225 0.0691 Uiso 1 2 calc R . .
H11K H 0.25029 -0.0913 0.05182 0.0720 Uiso 1 2 calc R . .
H14K H 0.54852 0.07296 0.22628 0.0464 Uiso 1 2 calc R . .
H15K H 0.63537 0.36239 0.23747 0.0486 Uiso 1 2 calc R . .
H17K H 0.94463 0.26778 0.2549 0.0539 Uiso 1 2 calc R . .
H17L H 0.86262 0.41581 0.25535 0.0539 Uiso 1 2 calc R . .
H18K H 0.8661 0.16094 0.32822 0.0643 Uiso 1 2 calc R . .
H18L H 0.83268 0.33201 0.34212 0.0643 Uiso 1 2 calc R . .
H19K H 0.67773 0.1046 0.30713 0.0576 Uiso 1 2 calc R . .
H19L H 0.64406 0.27269 0.32484 0.0576 Uiso 1 2 calc R . .
H20K H 0.74327 0.02138 0.17729 0.0495 Uiso 1 2 calc R . .
H20L H 0.80186 0.01241 0.24067 0.0495 Uiso 1 2 calc R . .
H20M H 0.88027 0.05799 0.19256 0.0495 Uiso 1 2 calc R . .
H21K H 0.69482 0.35541 0.14892 0.0489 Uiso 1 2 calc R . .
H24K H 1.1172 0.28525 0.11893 0.0542 Uiso 1 2 calc R . .
H27K H 0.73694 0.39577 0.05616 0.0535 Uiso 1 2 calc R . .

loop_
_atom_site_aniso_label
_atom_site_aniso_U_11
_atom_site_aniso_U_22
_atom_site_aniso_U_33
_atom_site_aniso_U_12
_atom_site_aniso_U_13
_atom_site_aniso_U_23
N1A 0.049(2) 0.031(2) 0.068(3) 0.0047(19) 0.0047(19) 0.002(2)
N2A 0.049(2) 0.031(2) 0.068(3) 0.0047(19) 0.0047(19) 0.002(2)
C3A 0.049(2) 0.031(2) 0.068(3) 0.0047(19) 0.0047(19) 0.002(2)
C4A 0.049(2) 0.031(2) 0.068(3) 0.0047(19) 0.0047(19) 0.002(2)
N5A 0.049(2) 0.031(2) 0.068(3) 0.0047(19) 0.0047(19) 0.002(2)
C6A 0.049(2) 0.031(2) 0.068(3) 0.0047(19) 0.0047(19) 0.002(2)
C7A 0.049(2) 0.031(2) 0.068(3) 0.0047(19) 0.0047(19) 0.002(2)
C8A 0.049(2) 0.031(2) 0.068(3) 0.0047(19) 0.0047(19) 0.002(2)
C9A 0.049(2) 0.031(2) 0.068(3) 0.0047(19) 0.0047(19) 0.002(2)
C10A 0.049(2) 0.031(2) 0.068(3) 0.0047(19) 0.0047(19) 0.002(2)
C11A 0.049(2) 0.031(2) 0.068(3) 0.0047(19) 0.0047(19) 0.002(2)
N1D 0.059(3) 0.029(2) 0.087(3) 0.003(2) 0.010(2) -0.002(2)
N2D 0.059(3) 0.029(2) 0.087(3) 0.003(2) 0.010(2) -0.002(2)
C3D 0.059(3) 0.029(2) 0.087(3) 0.003(2) 0.010(2) -0.002(2)
C4D 0.059(3) 0.029(2) 0.087(3) 0.003(2) 0.010(2) -0.002(2)
N5D 0.059(3) 0.029(2) 0.087(3) 0.003(2) 0.010(2) -0.002(2)

C6D 0.059(3) 0.029(2) 0.087(3) 0.003(2) 0.010(2) -0.002(2)
C7D 0.059(3) 0.029(2) 0.087(3) 0.003(2) 0.010(2) -0.002(2)
C8D 0.059(3) 0.029(2) 0.087(3) 0.003(2) 0.010(2) -0.002(2)
C9D 0.059(3) 0.029(2) 0.087(3) 0.003(2) 0.010(2) -0.002(2)
C10D 0.059(3) 0.029(2) 0.087(3) 0.003(2) 0.010(2) -0.002(2)
C11D 0.059(3) 0.029(2) 0.087(3) 0.003(2) 0.010(2) -0.002(2)
C12A 0.046(3) 0.025(3) 0.052(3) 0.004(2) -0.002(2) 0.008(2)
O13A 0.060(3) 0.026(2) 0.057(2) -0.0047(18) 0.0122(19) -0.0029(17)
N14A 0.053(3) 0.028(2) 0.042(2) -0.001(2) 0.003(2) 0.0019(19)
C15A 0.043(3) 0.029(3) 0.059(4) 0.004(2) 0.006(3) 0.000(3)
C16A 0.040(3) 0.032(3) 0.058(4) -0.001(2) 0.010(3) -0.001(2)
C17A 0.054(4) 0.053(4) 0.064(4) -0.001(3) 0.009(3) 0.002(3)
C18A 0.046(4) 0.057(4) 0.073(4) -0.001(3) 0.010(3) -0.001(3)
C19A 0.043(3) 0.038(3) 0.064(4) 0.003(3) 0.000(3) -0.003(3)
C20A 0.075(4) 0.043(3) 0.038(3) -0.004(3) 0.004(3) 0.003(3)
N21A 0.064(3) 0.044(3) 0.038(2) -0.010(3) 0.010(2) -0.003(2)
C22A 0.051(4) 0.048(3) 0.059(4) 0.003(3) 0.010(3) -0.004(3)
N23A 0.078(4) 0.067(4) 0.053(3) -0.006(3) 0.022(3) 0.007(3)
C24A 0.098(6) 0.064(5) 0.066(5) 0.008(4) 0.027(4) 0.013(4)
C25A 0.107(6) 0.051(4) 0.056(4) 0.007(4) 0.012(4) -0.004(4)
N26A 0.098(5) 0.064(4) 0.060(4) -0.001(4) 0.010(3) -0.010(3)
C27A 0.082(5) 0.050(4) 0.062(4) -0.006(4) 0.000(4) -0.008(3)
C28A 0.144(9) 0.068(6) 0.059(5) 0.029(6) 0.014(5) 0.011(4)
F29A 0.209(7) 0.098(4) 0.055(3) 0.027(5) 0.025(3) -0.001(3)
F30A 0.184(6) 0.110(5) 0.071(3) 0.021(5) -0.031(4) -0.009(3)
F31A 0.259(8) 0.082(4) 0.066(3) 0.063(5) 0.018(4) -0.014(3)
N1K 0.043(3) 0.047(3) 0.044(3) -0.002(2) 0.001(2) -0.004(2)
N2K 0.057(4) 0.098(5) 0.052(3) -0.008(3) 0.008(3) -0.010(3)
C3K 0.065(5) 0.072(5) 0.049(4) 0.001(4) 0.010(3) -0.006(3)
C4K 0.034(3) 0.056(4) 0.064(4) -0.013(3) 0.011(3) -0.009(3)
N5K 0.038(3) 0.053(3) 0.064(3) -0.003(2) 0.004(2) -0.012(3)
C6K 0.046(3) 0.034(3) 0.049(3) -0.005(3) -0.002(3) -0.001(2)
C7K 0.036(3) 0.028(3) 0.061(4) 0.011(2) 0.002(3) 0.009(2)
C8K 0.041(3) 0.038(3) 0.066(4) 0.006(3) 0.011(3) 0.008(3)
C9K 0.027(3) 0.042(3) 0.076(4) 0.005(2) 0.002(3) 0.009(3)
C10K 0.038(3) 0.052(4) 0.080(5) -0.006(3) -0.008(3) 0.012(3)
C11K 0.050(4) 0.055(4) 0.072(4) -0.017(3) -0.004(3) 0.003(3)
C12K 0.041(3) 0.030(3) 0.043(3) 0.006(2) 0.010(2) -0.006(2)
O13K 0.046(2) 0.0277(19) 0.049(2) -0.0005(16) 0.0079(17) 0.0042(16)
N14K 0.036(2) 0.026(2) 0.054(3) -0.0036(18) 0.004(2) 0.0004(19)
C15K 0.035(3) 0.030(3) 0.056(3) -0.002(2) 0.004(2) -0.006(2)
C16K 0.047(3) 0.029(3) 0.052(3) -0.001(2) 0.006(2) 0.007(2)
C17K 0.042(3) 0.037(3) 0.053(3) 0.001(2) -0.006(3) 0.002(3)
C18K 0.060(4) 0.053(4) 0.045(3) -0.005(3) -0.006(3) -0.001(3)
C19K 0.057(4) 0.042(3) 0.047(3) -0.011(3) 0.014(3) -0.000(3)
C20K 0.042(3) 0.032(3) 0.050(3) 0.003(2) 0.006(2) 0.005(2)
N21K 0.035(2) 0.029(2) 0.056(3) 0.0007(19) -0.001(2) 0.007(2)
C22K 0.042(3) 0.026(3) 0.060(4) -0.011(2) -0.002(3) 0.002(2)

N23K 0.038(2) 0.030(2) 0.046(3) -0.0072(19) 0.003(2) 0.0077(19)
C24K 0.033(3) 0.037(3) 0.064(4) -0.001(2) 0.001(3) -0.000(3)
C25K 0.037(3) 0.035(3) 0.058(4) 0.006(2) 0.002(3) -0.000(3)
N26K 0.050(3) 0.043(3) 0.047(3) 0.000(2) 0.015(2) -0.002(2)
C27K 0.054(4) 0.029(3) 0.049(3) 0.004(3) -0.000(3) -0.001(2)
C28K 0.069(5) 0.081(6) 0.060(5) 0.013(4) 0.024(4) 0.013(4)
F29K 0.049(2) 0.102(4) 0.083(3) 0.011(2) 0.018(2) 0.026(3)
F30K 0.064(3) 0.252(8) 0.058(3) 0.030(4) 0.024(2) 0.005(4)
F31K 0.107(4) 0.082(4) 0.182(6) 0.022(3) 0.086(4) 0.059(4)

_computing_data_collection 'RAPID AUTO (Rigaku, ????)'
_computing_cell_refinement 'RAPID AUTO'
_computing_data_reduction 'RAPID AUTO'
_computing_structure_solution
'Il Milione (Burla, et al., 2007)'
_computing_structure_refinement
'SHELXL2013 (Sheldrick, 2008)'
_computing_publication_material
'CrystalStructure 4.1 (Rigaku, 2014)'
_computing_molecular_graphics 'CrystalStructure 4.1'

Supplementary Table 1: Projection targets of DRN Vglut3 neurons.

id	name	acronym	Vglut3-cre
	**Extra-cortical regions**		
4	Inferior colliculus	IC	+
23	Anterior amygdalar area	AAA	++
30	Periventricular hypothalamic nucleus, anterior part	PVa	-
38	Paraventricular hypothalamic nucleus	PVH	+
56	Nucleus accumbens	ACB	+
88	Anterior hypothalamic nucleus	AHN	+
100	Interpeduncular nucleus	IPN	-
105	Superior olivary complex, medial part	SOCm	-
118	Periventricular hypothalamic nucleus, intermediate part	PVi	-
126	Periventricular hypothalamic nucleus, posterior part	PVp	+
133	Periventricular hypothalamic nucleus, preoptic part	PVpo	+
147	Locus ceruleus	LC	-
155	Lateral dorsal nucleus of thalamus	LD	-
173	Retrochiasmatic area	RCH	++
178	Ventral part of the lateral geniculate complex	LGv	-
181	Nucleus of reunions	RE	-
186	Lateral habenula	LH	-
194	Lateral hypothalamic area	LHA	+++
202	Medial vestibular nucleus	MV	++
210	Lateral mammillary nucleus	LM	+
215	Anterior pretectal nucleus	APN	++
218	Lateral posterior nucleus of the thalamus	LP	-
223	Arcuate hypothalamic nucleus	ARH	-
226	Lateral preoptic area	LPO	+
246	Midbrain reticular nucleus, retrorubral area	RR	-
263	Anteroventral preoptic nucleus	AVP	+
272	Anteroventral periventricular nucleus	AVPV	+
286	Suprachiasmatic nucleus	SCH	-
294	Superior colliculus, motor related	SCm	+
298	Magnocellular nucleus	MA	+
303	Basolateral amygdalar nucleus, anterior part	BLAa	++
311	Basolateral amygdalar nucleus, posterior part	BLAp	-
319	Basomedial amygdalar nucleus	BMA	+
342	Substantia innominata	SI	+
347	Subparaventricular zone	SBPV	-
351	Bed nuclei of the stria terminalis	BST	-
354	Medulla	MY	+
364	Parasubthalamic nucleus	PSTN	+
366	Submedial nucleus of the thalamus	SMT	++
381	Substantia nigra, reticular part	SNr	++
390	Supraoptic nucleus	SO	+
403	Medial amygdalar nucleus	MEA	+

451	Basolateral amygdalar nucleus, ventral part	BLAv	++
470	Subthalamic nucleus	STN	+
491	Medial mammillary nucleus	MM	+
502	Subiculum	SUB	-
515	Medial preoptic nucleus	MPN	+
523	Medial preoptic area	MPO	++
525	Supramammillary nucleus	SUM	+
531	Medial pretectal area	MPT	-
536	Central amygdalar nucleus	CEA	+
566	Postpiriform transition area	TR	++
583	Claustrum	CLA	++
589	Taenia tecta	TT	+
596	Diagonal band nucleus	NDB	+++
614	Tuberal nucleus	TU	++
639	Cortical amygdalar area, anterior part	COAa	+
647	Cortical amygdalar area, posterior part	COAp	++
672	Caudoputamen	CP	+
693	Ventromedial hypothalamic nucleus	VMH	+
749	Ventral tegmental area	VTA	++
754	Olfactory tubercle	OT	+++
780	Posterior amygdalar nucleus	PA	++
788	Piriform-amygdalar area	PAA	++
797	Zona incerta	ZI	+
830	Dorsomedial nucleus of the hypothalamus	DMH	++
842	Superior colliculus, superficial gray layer	SCsg	+ -
881	Parabrachial nucleus, lateral division	PBl	++
931	Pontine gray	PG	+
946	Posterior hypothalamic nucleus	PH	++
980	Dorsal premammillary nucleus	PMd	+ -
1004	Ventral premammillary nucleus	PMv	+ -
1093	Pontine reticular nucleus, caudal part	PRNc	-

	**Cortex**		
	All regions - Cortical layer 1		+++
918	Entorhinal area, lateral part - Layers 2-3-4	ENTl	++
378	Supplemental somatosensory area - Layers 4 - 5	SSs	++
541	Temporal association areas - layers 2-3	TEa	++
985	Primary motor area - layers 2-3	MOp	++
345	Primary somatosensory area, mouth - layers 2-3-4	SSp-m	++
104	Agranular insular area, dorsal part - layers 2-3	AId	++
993	Secondary motor area - layer 5	MOs	++
1057	Gustatory areas, layers 2-3-4	GU	++

Supplementary Table 2: Molecular targets enriched in DRN Vglut3 neurons in comparison to GENSAT database.

	IDs	Symbols	combined_chisq	mean_l2fc
1	18812	Prl2c3	147.70	18.04
2	56183	Nmu	690.72	16.44
3	26366	Ceacam10	937.77	16.36
4	242646	Tctex1d4	952.46	15.71
5	22788	Zp3	1179.46	15.66
6	108112	Eif4ebp3	121.29	14.90
7	74481	Batf2	1044.47	14.81
8	15567	Slc6a4	2298.66	14.35
9	20293	Ccl12	1056.74	14.22
10	320415	Gchfr	5306.01	14.21
11	12836	Col7a1	1319.85	13.78
12	67085	1700024G13Rik	1584.23	13.60
13	50916	Irx4	1715.09	13.57
14	57429	Sult5a1	1183.17	13.57
15	17972	Ncf4	1231.40	13.33
16	71898	Apol9b	968.64	13.18
17	66758	Zfp474	1443.04	13.12
18	53603	Tslp	1018.00	13.06
19	76218	6430710C18Rik	1585.21	12.86
20	67470	Abcg8	669.62	12.85
21	12958	Cryba2	1251.00	12.48
22	109215	Lncbate1	104.22	12.45
23	226278	Prlhr	1740.37	12.35
24	216343	Tph2	4304.49	12.26
25	116903	Calcb	1997.35	12.19
26	260298	Fev	2685.32	12.14
27	545667	Fam159a	1688.36	12.06
28	55985	Cxcl13	1660.36	11.77
29	100302688	Gm17455	1483.53	11.59
30	328779	Hs3st6	1726.97	11.25
31	22226	Ucn	1234.99	11.04
32	18096	Nkx6-1	1791.89	10.93
33	66402	Sln	1536.31	10.78
34	381337	Fam178b	1238.31	10.72
35	50929	Il22	1097.41	10.37
36	237038	Nox1	1176.19	10.35
37	235345	4833427G06Rik	1406.56	10.34
38	17751	Mt3	6220.36	10.31
39	67182	Pdzk1ip1	1575.62	10.25
40	100073351	Yy2	533.86	10.23
41	223672	Apol9a	1934.75	10.06
42	74511	Lrrc17	2023.71	9.87
43	19222	Ptgir	887.40	9.84
44	246730	Oas1a	1572.90	9.76
45	212108	Rln3	1211.33	9.73
46	101744	Acp7	1184.47	9.68
47	74175	Crct1	78.44	9.64
48	20618	Sncg	4556.44	9.56
49	170706	Tmem37	1484.52	9.55
50	64379	Irx6	1788.46	9.47
51	76615	Got1l1	1382.08	9.43
52	22635	Zan	1218.95	9.42
53	75040	Efcab10	3337.04	9.33
54	13166	Dbh	1858.73	9.32
55	72249	1700019G24Rik	213.98	9.30
56	218739	Sntn	1406.20	9.23
57	75465	Dynlrb2	1946.57	9.14
58	13195	Ddc	5485.44	9.07
59	16619	Klk1b27	786.21	9.04
60	269637	Cnpy1	2254.40	9.04
61	17868	Mybpc3	934.86	9.02
62	14695	Gnb3	1272.23	8.99
63	211429	Pla2g4b	971.68	8.90
64	13798	En1	2955.76	8.87
65	54422	Barhl1	1992.44	8.84
66	12895	Cpt1b	1283.26	8.83
67	26368	Ceacam9	1259.50	8.83

	IDs	Symbols	combined_chisq	mean_l2fc
798	27883	Tango2	3381.51	3.88
799	68375	Ndufa8	4011.38	3.88
800	68977	Haghl	2799.24	3.88
801	69379	C8g	487.43	3.88
802	14707	Gng5	2618.83	3.88
803	67326	1700037H04Rik	2179.14	3.87
804	69216	Svbp	1228.20	3.87
805	67713	Dnajc19	4219.21	3.87
806	104601	Mycbpap	727.79	3.86
807	13595	Ebp	1576.37	3.86
808	68523	Fam96b	1491.84	3.86
809	24030	Mrps12	2064.51	3.86
810	76917	Flywch2	1392.27	3.86
811	17150	Mfap2	633.98	3.86
812	73103	3110009E18Rik	564.94	3.86
813	84682	Cox4i2	596.28	3.86
814	69878	Snrpf	2406.77	3.86
815	27999	Fam3c	4767.13	3.86
816	68565	Mrps18a	2127.50	3.85
817	553095	Gm17750	642.00	3.85
818	66117	Fmc1	1629.54	3.85
819	545192	Baiap3	1573.41	3.85
820	272411	B3gnt6	520.78	3.85
821	227327	B3gnt7	585.90	3.85
822	21681	Alyref	2171.62	3.84
823	378462	Morn2	1497.50	3.84
824	70257	2010107E04Rik	2741.76	3.84
825	76183	Celf6	1750.27	3.84
826	52020	Umodl1	291.53	3.84
827	69964	2810403D21Rik	582.54	3.83
828	72243	1700012D01Rik	429.44	3.83
829	17965	Nbl1	1497.67	3.82
830	11556	Adrb3	606.53	3.82
831	60532	Wtap	2730.43	3.82
832	68194	Ndufb4	3166.47	3.82
833	71564	Izumo4	1753.57	3.82
834	66400	Alkbh7	1911.26	3.82
835	69798	1810044D09Rik	480.15	3.81
836	21924	Tnnc1	797.11	3.81
837	71446	Wrb	5882.36	3.81
838	13733	Adgre1	467.63	3.81
839	20262	Stmn3	3086.27	3.81
840	16852	Lgals1	982.81	3.81
841	237858	Tusc5	684.50	3.81
842	12070	Bex3	3347.22	3.81
843	68033	Cox19	1906.76	3.81
844	108832	Tmem74b	1066.44	3.80
845	18002	Nedd8	2336.49	3.80
846	403178	Plcxd1	1796.34	3.80
847	50784	Plpp2	1051.27	3.80
848	66359	Cox20	2555.49	3.80
849	12368	Casp6	716.10	3.80
850	30059	Timm10	2029.36	3.80
851	66423	Coprs	2670.37	3.80
852	70451	Dhrs13	1451.88	3.79
853	56322	Timm22	2700.71	3.79
854	100040531	Dynlt1f	1553.48	3.79
855	72536	Tagap	603.26	3.79
856	28077	Med10	1894.61	3.79
857	66379	Cox14	2630.73	3.79
858	69590	Gpx8	625.54	3.78
859	14840	Gsg1	356.44	3.78
860	22248	Unc119	2246.66	3.78
861	415115	Neurl2	612.74	3.78
862	50786	Hs6st2	2582.55	3.78
863	72657	Selenoh	1961.69	3.78
864	245020	Slc35g2	1692.62	3.77

68	54615	Npff	1404.91	8.82
69	77682	9230102O04Rik	351.58	8.80
70	73321	1700042O10Rik	423.48	8.80
71	231290	Slc10a4	2772.48	8.71
72	14399	Gabra6	1119.16	8.70
73	20957	Sycp1	989.07	8.70
74	14419	Gal	2153.53	8.58
75	67578	Patl2	1046.51	8.57
76	11522	Adh1	1101.78	8.56
77	75434	1700001C02Rik	676.34	8.53
78	17121	Mxd3	1778.74	8.48
79	382384	Odf3l2	1234.03	8.47
80	241158	Ankmy1	1306.66	8.44
81	22296	Vmn1r51	458.03	8.43
82	69387	Dnajb13	1216.81	8.37
83	100038374	Gm10714	548.90	8.33
84	192200	Wfdc12	2545.05	8.32
85	14462	Gata3	5011.13	8.27
86	70110	Ifi35	1447.72	8.16
87	70036	Dancr	1948.63	8.13
88	14601	Ghrh	1275.74	8.12
89	626359	Wdr93	998.48	8.09
90	171207	Arhgap4	995.38	8.08
91	101202684	Uchl1os	1372.48	8.04
92	14913	Guca1a	638.85	8.04
93	20753	Sprr1a	777.28	7.97
94	14528	Gch1	2728.55	7.96
95	69772	Bdh2	1400.57	7.94
96	21683	Tecta	1207.50	7.94
97	78491	Tspan2os	1116.12	7.93
98	381759	Wee2	900.29	7.92
99	20715	Serpina3g	1784.57	7.88
100	20198	S100a4	1416.25	7.82
101	69987	Spaca9	1003.83	7.81
102	19773	Rln1	981.18	7.80
103	71840	Tekt4	1223.96	7.79
104	114875	Plcz1	2060.85	7.77
105	56753	Tacstd2	1145.49	7.76
106	74466	Mfsd13b	911.66	7.74
107	102640359	LOC102640359	1437.35	7.74
108	208613	Tmem212	709.00	7.71
109	434858	Gm5643	2259.79	7.70
110	338403	Cndp1	726.83	7.61
111	17314	Mgmt	1550.45	7.60
112	212032	Hk3	990.21	7.51
113	13345	Twist2	1781.63	7.46
114	100526556	Mir3091	1578.54	7.42
115	12705	Cited1	3659.40	7.39
116	328235	Gm5083	1640.95	7.33
117	270150	Ccdc153	1971.79	7.31
118	11806	Apoa1	1380.12	7.26
119	12075	Bfsp1	1443.76	7.26
120	69047	Atp2c2	987.17	7.21
121	71111	Gpr39	670.64	7.21
122	69797	1600029I14Rik	369.36	7.18
123	434147	D930028M14Rik	771.72	7.17
124	56773	Chst5	699.84	7.17
125	140498	Rxfp2	1497.22	7.13
126	77914	Krtap17-1	402.92	7.11
127	69434	Snhg10	3037.45	7.09
128	14461	Gata2	1904.09	7.09
129	12311	Calcr	1783.04	7.07
130	102466257	Mir8114	2884.21	7.07
131	16667	Krt17	777.92	7.06
132	72088	Ush1c	626.85	7.05
133	73349	1700042G15Rik	1860.37	7.04
134	380842	Stmnd1	611.81	6.99
135	69325	1700012B09Rik	1173.68	6.97
136	434423	Dppa5a	772.95	6.96
865	100504104	Gm16062	655.04	3.77
866	751554	Mir678	592.81	3.77
867	333182	Cox6b2	659.53	3.77
868	79044	Mrps34	1793.27	3.77
869	18292	Sebox	425.90	3.76
870	69573	Hilpda	1380.19	3.76
871	78416	Rnase6	356.69	3.76
872	232680	Cpa2	613.01	3.76
873	12479	Cd1d1	1063.97	3.76
874	20084	Rps18	2029.25	3.76
875	12815	Col11a2	468.66	3.76
876	75693	3010001F23Rik	759.36	3.75
877	52530	Nhp2	1581.65	3.75
878	76501	Commd9	1799.12	3.75
879	107146	Glyat	215.20	3.74
880	269954	Ttll13	561.10	3.74
881	73647	Capn9	345.38	3.74
882	14038	Wfdc18	326.83	3.74
883	117148	Necab2	1780.36	3.74
884	56506	Cib2	1585.47	3.73
885	107197	Uqcc3	1597.61	3.73
886	237313	Il20ra	321.98	3.73
887	77875	Cyp4f41-ps	506.61	3.73
888	12856	Cox17	2532.35	3.73
889	319158	Hist1h4i	582.80	3.73
890	79043	Spsb3	1437.58	3.73
891	14109	Fau	2061.86	3.72
892	68347	Mettl26	1721.35	3.72
893	66191	Ier3ip1	3112.01	3.72
894	66046	Ndufb5	2428.66	3.72
895	213948	Atg9b	1675.00	3.72
896	73234	3110079O15Rik	274.33	3.72
897	11844	Arf5	1853.11	3.71
898	75731	Idnk	3327.43	3.71
899	75099	Lysmd4	2864.76	3.71
900	66167	Tma7	4339.33	3.71
901	11606	Agt	1089.79	3.70
902	14171	Fgf17	264.41	3.70
903	100503961	Gm19990	151.61	3.70
904	66915	Cops9	2793.14	3.70
905	69327	1700007K13Rik	877.99	3.70
906	94064	Mrpl27	3954.46	3.70
907	78304	Naa38	1956.13	3.69
908	213527	Pth2r	503.09	3.69
909	57373	Akip1	1002.92	3.69
910	67126	Atp5e	1689.80	3.69
911	54402	Stk19	1508.34	3.69
912	75578	Fggy	1504.53	3.69
913	54127	Rps28	1581.10	3.68
914	13877	Erh	2662.69	3.68
915	75692	Nr2c2ap	1322.57	3.68
916	57316	C1d	2181.06	3.68
917	243867	Fbxo46	1627.79	3.68
918	16949	Loxl1	405.71	3.68
919	14427	Galr1	640.69	3.68
920	67749	Mgarp	356.52	3.67
921	12864	Cox6c	2481.30	3.67
922	18510	Pax8	608.65	3.67
923	67116	Cuedc2	2560.64	3.67
924	71913	Tmem79	512.26	3.67
925	20655	Sod1	4083.01	3.67
926	209268	Igsf1	852.81	3.67
927	71785	Pdgfd	538.74	3.67
928	16952	Anxa1	645.84	3.67
929	56312	Nupr1	616.13	3.66
930	78670	Plekhj1	2008.79	3.66
931	12828	Col4a3	891.69	3.66
932	235312	C1qtnf5	553.46	3.66
933	56424	Stub1	1614.20	3.66

137	58992	F12	949.18	6.94
138	11944	Atp4a	1301.31	6.94
139	109267	Ssc4d	1517.98	6.92
140	56390	Sssca1	1290.66	6.87
141	407793	BC039966	1231.05	6.82
142	631323	Gm12250	1046.86	6.82
143	77945	Rpgrip1	3673.88	6.80
144	50501	Prok2	1250.62	6.80
145	71236	Rsph14	926.27	6.79
146	545758	Gm5868	1258.80	6.78
147	56734	Tulp2	1235.79	6.72
148	100502834	Gm15972	1082.67	6.71
149	71660	Rarres2	964.68	6.67
150	12587	Mia	746.84	6.66
151	78194	4930546K05Rik	2130.77	6.63
152	13449	Dok2	915.67	6.63
153	380787	A230065H16Rik	1144.27	6.62
154	16178	Il1r2	767.96	6.60
155	381418	Ctxn2	6213.85	6.59
156	100504121	Med9os	1408.88	6.58
157	230777	Hcrtr1	1234.32	6.57
158	15372	Hmx2	792.17	6.57
159	75429	Fam183b	2402.04	6.55
160	27220	Cartpt	2476.19	6.53
161	75495	Morn5	923.07	6.52
162	623131	Prr19	831.78	6.51
163	72269	Cda	1494.09	6.50
164	317755	Zar1	858.95	6.49
165	414116	D630024D03Rik	775.76	6.43
166	73474	Snhg9	1135.97	6.39
167	76113	Lpo	1880.64	6.36
168	14963	H2-Bl	1008.97	6.35
169	94346	Tmem40	892.87	6.35
170	100034726	Gm15772	479.64	6.35
171	68354	Plekhd1os	284.18	6.32
172	280621	Selenov	1606.80	6.31
173	70593	Evx1os	176.24	6.30
174	107375	Slc25a45	1243.59	6.29
175	69454	Clic3	840.85	6.29
176	19711	Resp18	5613.49	6.27
177	330086	Gm10440	805.83	6.26
178	100169864	Gm44504	1321.66	6.25
179	791403	Mhrt	977.49	6.24
180	433367	Gm5532	1182.89	6.23
181	329831	Fam166b	847.68	6.21
182	23834	Cdc6	1136.83	6.20
183	24117	Wif1	1288.57	6.18
184	15373	Hmx3	194.18	6.17
185	15564	Htr5b	1231.45	6.15
186	103611159	Gm38667	1149.48	6.15
187	218963	Gm1821	3486.55	6.13
188	78625	1700061G19Rik	1201.42	6.12
189	11814	Apoc3	448.66	6.12
190	433456	4631405J19Rik	287.93	6.11
191	100504399	C130021I20Rik	1240.73	6.11
192	73121	Rflna	1089.90	6.10
193	75600	Calml4	639.72	6.08
194	79362	Bhlhe41	10917.98	6.08
195	67405	Nts	1913.33	6.07
196	14686	Gnat2	750.80	6.06
197	100040880	Gm3020	822.82	6.06
198	317750	Slc24a5	1847.50	6.05
199	70772	Ggnbp1	1038.38	6.05
200	74720	Tmem114	1304.60	6.03
201	12683	Cidea	1488.86	5.99
202	74023	Rd3	793.63	5.98
203	627280	Vmn1r90	35.46	5.97
204	434438	Ccdc36	2061.73	5.94
205	11859	Phox2a	154.34	5.92

934	68475	Ssna1	1750.93	3.66
935	70123	2210013O21Rik	2867.95	3.66
936	94043	Tm2d1	2211.57	3.66
937	100616095	Snhg18	720.78	3.66
938	170720	Card14	196.81	3.65
939	74071	Lmntd1	694.66	3.65
940	73747	1110034G24Rik	1136.08	3.65
941	77675	5033406O09Rik	560.24	3.64
942	69029	Smdt1	2358.63	3.64
943	11957	Atp5j	4713.12	3.64
944	278240	Spin2c	1327.94	3.64
945	68576	Lamtor5	3514.49	3.64
946	66170	Chchd5	1677.01	3.64
947	11876	Artn	379.38	3.63
948	68859	Smim1	1121.92	3.63
949	74246	Gale	1151.98	3.63
950	69747	Zswim7	1238.53	3.63
951	67880	Dcxr	809.76	3.63
952	213019	Pdlim2	1241.79	3.63
953	622208	Gm6297	663.79	3.63
954	13014	Cstb	2607.48	3.63
955	56174	Nagk	1591.67	3.63
956	71242	Spata24	761.81	3.63
957	16173	Il18	1005.79	3.63
958	56438	Rbx1	5123.08	3.62
959	67308	Mrpl46	2467.83	3.62
960	73316	Calr3	603.36	3.62
961	212377	Mms22l	553.56	3.62
962	68355	2010204K13Rik	1631.80	3.62
963	56069	Il17b	260.20	3.62
964	237891	Gas2l2	608.41	3.62
965	68212	Tmbim4	3345.46	3.62
966	27280	Phlda3	2242.62	3.61
967	378702	Serf2	4132.73	3.61
968	69961	Rpp25l	1877.14	3.61
969	56520	Nme4	780.68	3.61
970	19188	Psme2	2009.10	3.61
971	54153	Rasa4	590.82	3.61
972	23934	Ly6h	1939.79	3.61
973	67042	Ift27	1432.17	3.61
974	18484	Pam	3846.21	3.60
975	69168	Bola1	1192.96	3.60
976	77920	A330102I10Rik	1182.21	3.60
977	52668	Ifi27	1769.29	3.60
978	100504333	Gm16712	413.01	3.60
979	619605	Zcchc17	4769.72	3.60
980	17256	Mea1	1825.33	3.60
981	58251	Cep295nl	316.12	3.60
982	74359	4931414P19Rik	904.11	3.59
983	20768	Sephs2	2960.40	3.59
984	108897	Aif1l	780.79	3.59
985	69638	Enho	2275.25	3.59
986	66218	Ndufb9	3708.00	3.59
987	28126	Nop16	2075.40	3.59
988	244091	Fsd2	304.16	3.59
989	13732	Emp3	656.37	3.59
990	78473	Skap1	1456.02	3.58
991	66194	Pycrl	2236.68	3.58
992	226040	Tmem252	489.39	3.58
993	11951	Atp5g1	2125.05	3.58
994	12405	Cbln2	1596.71	3.58
995	109594	Lmo1	1040.83	3.58
996	218693	Paip1	1971.59	3.58
997	15465	Hrh1	1067.84	3.58
998	20733	Spint2	1475.88	3.58
999	56282	Mrpl12	2746.51	3.57
1000	65963	Tmem176b	1248.39	3.57
1001	20638	Snrpb	1804.73	3.57
1002	103844	Inca1	830.50	3.57

206	20186	Nr1h4	486.58	5.92
207	20519	Slc22a3	2160.10	5.89
208	27356	Insl6	545.76	5.88
209	100216537	Snord104	1658.45	5.88
210	20194	S100a10	2904.08	5.87
211	66091	Ndufa3	4729.68	5.87
212	214084	Slc18a2	3189.85	5.87
213	69186	Tmem256	3037.45	5.86
214	74934	Armc4	674.92	5.86
215	93721	Cpn1	232.87	5.85
216	216635	Hbq1a	554.33	5.84
217	19132	Prph	1233.62	5.84
218	224912	Crb3	700.28	5.83
219	66120	Fkbp11	1232.14	5.82
220	546049	C330021F23Rik	1228.75	5.81
221	14999	H2-DMb1	790.26	5.80
222	319166	Hist1h2ae	796.77	5.80
223	240916	Vsig8	530.53	5.79
224	328789	Lhfpl5	2406.79	5.78
225	20532	Slc3a1	696.42	5.78
226	71583	9130008F23Rik	776.31	5.78
227	69812	Abhd11os	1552.68	5.77
228	50928	Klrg1	552.61	5.76
229	66594	Uqcr11	3224.27	5.75
230	71860	Cfap52	841.62	5.75
231	78403	2900041M22Rik	920.84	5.74
232	67267	Uqcc2	3215.16	5.73
233	320973	D330023K18Rik	2146.72	5.71
234	654359	Gm12338	847.13	5.69
235	106672	AI413582	3547.12	5.68
236	17189	Mb	1118.36	5.68
237	54352	Irx5	1973.89	5.66
238	243078	Tecrl	270.89	5.65
239	102075	Plekhg4	711.36	5.64
240	70458	2610318N02Rik	801.83	5.61
241	15375	Foxa1	1018.24	5.60
242	320923	Map7d3	158.37	5.60
243	17112	Tm4sf1	1098.51	5.60
244	12427	Ccna1	1128.82	5.55
245	67530	Uqcrb	4373.78	5.54
246	234700	Nrn1l	1083.79	5.54
247	381546	Ccdc24	703.34	5.53
248	436062	Fam92b	769.35	5.52
249	320208	Tmem91	4519.89	5.52
250	76642	1700113A16Rik	1296.51	5.52
251	66815	Mcub	1251.47	5.51
252	104759	Pld4	758.88	5.50
253	333670	Gm867	1102.76	5.49
254	69563	Mrln	464.09	5.48
255	20389	Sftpc	704.01	5.47
256	67775	Rtp4	1210.54	5.47
257	15410	Hoxb3	259.38	5.47
258	14130	Fcgr2b	905.92	5.47
259	14734	Gpc3	2442.74	5.46
260	75614	Rab26os	2288.37	5.46
261	15171	Hcrt	592.06	5.45
262	15013	H2-Q2	923.78	5.45
263	18545	Pcp2	1181.41	5.45
264	75784	1700007G11Rik	846.87	5.45
265	68039	Nmb	1200.10	5.45
266	101202	Hepacam2	1039.06	5.45
267	56351	Ptges3	7962.30	5.45
268	59005	Trappc2l	3862.75	5.44
269	71227	Daw1	220.17	5.44
270	16407	Itgae	741.32	5.43
271	15376	Foxa2	390.44	5.43
272	226527	BC026585	802.39	5.42
273	74076	4933406C10Rik	940.25	5.41
274	238021	Fscn2	810.99	5.41

1003	66055	Sf3b6	2999.87	3.57
1004	170677	Cdhr1	850.61	3.57
1005	69666	Psmg4	968.31	3.57
1006	22152	Tubb3	3389.74	3.57
1007	12857	Cox4i1	2441.26	3.57
1008	171210	Acot2	2822.79	3.57
1009	73600	1700120C14Rik	444.09	3.57
1010	233073	U2af1l4	1585.56	3.57
1011	382089	Ripply2	806.30	3.57
1012	102278	Cpne7	1474.96	3.56
1013	85030	Tnfrsf25	174.12	3.56
1014	100042049	Gm15421	1680.08	3.56
1015	14227	Fkbp2	3268.16	3.56
1016	13063	Cycs	6019.23	3.56
1017	30057	Timm8b	2215.61	3.56
1018	14548	Mrps33	2540.42	3.56
1019	73261	1700037C18Rik	787.29	3.56
1020	71907	Serpina9	557.62	3.56
1021	64685	Nmi	830.35	3.55
1022	100048895	9330020H09Rik	1159.51	3.55
1023	100503468	Gm14023	693.84	3.55
1024	319476	Lrtm1	1096.48	3.55
1025	73951	4930413G21Rik	551.06	3.55
1026	64659	Mrps14	3026.05	3.54
1027	114479	Slc5a5	1758.63	3.54
1028	81500	Sil1	1360.20	3.54
1029	20930	Surf1	2649.84	3.54
1030	76927	Tsacc	551.70	3.54
1031	381633	Gm1673	1061.75	3.54
1032	66278	Smim20	1688.44	3.54
1033	18245	Oaz1	3706.90	3.54
1034	433470	AA467197	310.48	3.53
1035	56748	Nfu1	5727.48	3.53
1036	56844	Tssc4	1831.21	3.53
1037	100041230	Hist1h4m	348.57	3.53
1038	14733	Gpc1	2198.59	3.53
1039	66357	Ostc	2044.82	3.53
1040	234388	Ccdc124	1483.76	3.53
1041	17713	Grpel1	3196.49	3.53
1042	69893	Coa7	2048.96	3.53
1043	15486	Hsd17b2	351.45	3.53
1044	67344	Tctex1d1	231.18	3.53
1045	66824	Pycard	510.85	3.52
1046	66310	Dpy30	2354.02	3.52
1047	626175	Gm6654	548.68	3.52
1048	319183	Hist1h2bj	445.00	3.52
1049	67865	Rgs10	1548.71	3.52
1050	100502888	Gm19434	620.37	3.52
1051	93757	Immp2l	755.08	3.52
1052	72821	Scn2b	5377.96	3.52
1053	67705	1810058I24Rik	1899.50	3.51
1054	18670	Abcb4	537.06	3.51
1055	71238	Sdhaf3	2140.39	3.51
1056	77414	C030013G03Rik	505.97	3.51
1057	277898	Slc15a5	854.99	3.51
1058	213541	Ythdf2	2410.20	3.51
1059	67945	Rpl41	1549.25	3.51
1060	20971	Sdc4	1019.93	3.51
1061	69303	1700001G11Rik	340.81	3.51
1062	21987	Tpd52l1	1692.99	3.50
1063	12310	Calca	700.03	3.50
1064	109672	Cyb5a	2648.13	3.50
1065	19921	Rpl19	1904.65	3.50
1066	76411	Ift43	1624.35	3.50
1067	66364	2310009A05Rik	983.67	3.50
1068	20200	S100a6	877.57	3.50
1069	12566	Cdk2	381.59	3.50
1070	12859	Cox5b	2506.97	3.50
1071	57749	Piwil1	389.46	3.50

275	68588	Cthrc1	3311.41	5.39
276	100034363	Tmsb15b2	1717.19	5.36
277	56642	Ankrd2	940.95	5.36
278	100568459	Bc1	580.42	5.36
279	67130	Ndufa6	5249.06	5.34
280	12257	Tspo	1183.60	5.34
281	18639	Pfkfb1	713.70	5.34
282	68393	Mogat1	609.58	5.33
283	14566	Gdf9	1049.07	5.33
284	78365	Lhx1os	1479.56	5.33
285	433224	Gm5512	338.08	5.33
286	319684	5031425F14Rik	386.00	5.33
287	66416	Ndufa7	3526.58	5.33
288	654465	Defb47	296.54	5.33
289	73904	4833412C05Rik	942.99	5.31
290	66353	Riiad1	1389.56	5.29
291	67264	Ndufb8	7175.63	5.29
292	109731	Maob	2971.97	5.29
293	71887	Ppm1j	683.36	5.28
294	64138	Ctsz	2633.97	5.27
295	100503859	1110015O18Rik	1360.31	5.24
296	30044	Opn4	542.88	5.24
297	54405	Ndufa1	4158.57	5.24
298	66181	Nop10	3896.88	5.24
299	75563	Dnali1	767.68	5.23
300	404545	Ano7	918.54	5.23
301	17349	Mlf1	1072.81	5.23
302	225638	Alpk2	978.39	5.23
303	66528	Smim5	378.48	5.22
304	629750	Gm11517	1347.01	5.22
305	66144	Atp6v1f	5104.15	5.21
306	22272	Uqcrq	3287.25	5.21
307	67303	3110045C21Rik	804.88	5.21
308	208188	Ghsr	1199.84	5.21
309	11705	Amh	861.29	5.20
310	66047	Mrpl54	3709.06	5.20
311	14296	Frat1	3651.56	5.19
312	666244	Tmsb15b1	1802.67	5.19
313	107260	Otub1	8950.13	5.19
314	13180	Pcbd1	3049.70	5.18
315	15186	Hdc	850.36	5.18
316	235043	Tmem205	2137.46	5.18
317	624713	Gm6525	829.63	5.17
318	102637366	Gm10037	1256.18	5.17
319	52469	Coa3	3885.54	5.17
320	71738	Mamdc2	1026.43	5.16
321	72709	C1qtnf6	702.66	5.15
322	69550	Bst2	839.24	5.15
323	237465	Ccdc38	1067.65	5.14
324	19152	Prtn3	876.07	5.14
325	13537	Dusp2	755.42	5.13
326	66774	Carlr	1268.06	5.12
327	69739	2410004I01Rik	598.23	5.12
328	26568	Slc27a3	1119.71	5.11
329	100040339	Gm2721	667.44	5.11
330	69875	Ndufa11	3065.39	5.10
331	194908	Pld6	1216.89	5.10
332	20422	Sem1	3337.18	5.10
333	66491	Polr2l	3607.42	5.10
334	14790	Grcc10	3273.29	5.09
335	100043229	Gm10046	720.52	5.08
336	100041306	Gm3264	676.08	5.07
337	236781	Gpr119	57.98	5.07
338	100039246	Plac9b	943.74	5.07
339	18383	Tnfrsf11b	1051.26	5.07
340	12777	Ccr10	721.71	5.06
341	12817	Col13a1	355.85	5.06
342	15040	H2-T23	2198.92	5.06
343	243537	Uroc1	987.33	5.06

1072	14373	G0s2	761.48	3.50
1073	13353	Dgcr6	1941.32	3.50
1074	225825	Cd226	172.28	3.49
1075	100503460	Gm19705	398.92	3.49
1076	54217	Rpl36	1546.04	3.49
1077	26922	Mecr	1101.87	3.49
1078	546143	Ccpg1os	1258.89	3.49
1079	12069	Bex2	3211.98	3.49
1080	68028	Rpl22l1	1729.85	3.49
1081	105827	Amigo2	1466.37	3.49
1082	67169	Nradd	317.90	3.49
1083	66411	Tbcb	2580.98	3.49
1084	319865	E130114P18Rik	604.95	3.49
1085	27660	1700088E04Rik	693.38	3.49
1086	27375	Tjp3	290.86	3.48
1087	107885	Mthfs	936.00	3.48
1088	12850	Coq7	1891.84	3.48
1089	68917	Hint2	1639.83	3.48
1090	66220	Zdhhc12	695.81	3.48
1091	12913	Creb3	1971.54	3.48
1092	27398	Mrpl2	1719.28	3.48
1093	11821	Aprt	1420.46	3.47
1094	26912	Gcat	786.57	3.47
1095	67606	Fibin	555.22	3.47
1096	399591	Tmsb15l	889.18	3.47
1097	18167	Npy2r	1194.40	3.47
1098	11548	Adra1b	1123.07	3.47
1099	245403	Dcaf12l2	761.29	3.47
1100	54120	Gipc2	840.39	3.47
1101	84094	Plvap	1049.60	3.47
1102	57344	As3mt	1401.92	3.47
1103	17984	Ndn	3533.63	3.47
1104	20892	Cenpx	1231.72	3.46
1105	68870	Ak8	772.60	3.46
1106	68350	Mul1	3695.49	3.46
1107	217151	Arl5c	1091.22	3.46
1108	17748	Mt1	1601.38	3.46
1109	72519	Tmem55a	4092.54	3.46
1110	66488	Fam136a	1671.55	3.46
1111	68936	Smim11	1473.93	3.46
1112	77866	E130102H24Rik	600.69	3.45
1113	434402	Gm5617	1285.99	3.45
1114	72693	Zcchc12	3164.46	3.45
1115	21402	Skp1a	3956.08	3.45
1116	66914	Vps28	2683.42	3.45
1117	27078	B9d1	1744.22	3.45
1118	17472	Gbp4	557.60	3.45
1119	381404	Pabpc1l	314.75	3.45
1120	67855	Asprv1	512.33	3.44
1121	67115	Rpl14	1634.59	3.44
1122	19941	Rpl26	1858.26	3.44
1123	432478	Tmprss9	358.57	3.44
1124	67279	Med31	1841.54	3.44
1125	67350	1700084E18Rik	364.68	3.44
1126	226654	Tstd1	447.24	3.44
1127	66309	Tmem128	1723.11	3.44
1128	16210	Impact	6645.99	3.44
1129	217140	Scrn2	554.34	3.44
1130	72273	Smim24	371.74	3.44
1131	227157	Mpp4	408.01	3.44
1132	380752	Tssc1	1652.37	3.43
1133	66184	Rps4l	1105.91	3.43
1134	29858	Pmm1	2360.19	3.43
1135	78653	Bola3	2300.31	3.43
1136	66258	Mrps17	2008.71	3.43
1137	406217	Bex4	1441.47	3.43
1138	319192	Hist2h2aa2	780.01	3.42
1139	14865	Gstm4	928.04	3.42
1140	109778	Blvra	1862.91	3.42

344	67199	Pfdn1	3416.53	5.05
345	16370	Irs4	1857.62	5.05
346	233066	Syne4	726.96	5.03
347	100503380	Snhg4	3213.80	5.03
348	66916	Ndufb7	3305.77	5.03
349	100038689	Gm10421	1047.74	5.02
350	625249	Gpx4	3504.67	5.01
351	18431	Oca2	786.82	5.01
352	78896	1500015O10Rik	637.87	5.01
353	17749	Polr2k	3703.59	5.00
354	78372	Snrnp25	2648.67	4.99
355	242681	Rab42	1447.10	4.98
356	330188	Ccdc63	671.00	4.98
357	432995	Smim22	273.65	4.98
358	100126242	Frs3os	356.91	4.98
359	100340	Smpdl3b	1120.77	4.97
360	20195	S100a11	1610.67	4.97
361	353236	Pcdhac1	860.34	4.97
362	75804	4930463O16Rik	1241.35	4.97
363	243612	Ssu2	239.48	4.96
364	219170	Fam216b	654.96	4.96
365	241112	Catip	813.93	4.95
366	621603	Aldh3b2	1180.33	4.95
367	213980	Fbxw10	850.43	4.95
368	68563	Dpm3	2283.47	4.94
369	14675	Gna14	717.07	4.94
370	76933	Ifi27l2a	601.77	4.94
371	239766	Rtp1	814.95	4.93
372	16988	Lst1	889.61	4.93
373	257882	Olfr1344	968.68	4.92
374	20044	Rps14	2655.50	4.91
375	19223	Ptgis	834.38	4.90
376	12865	Cox7a1	1235.26	4.89
377	20287	Sct	292.61	4.89
378	595136	Ndufs5	2574.41	4.89
379	68634	Tm2d3	4417.19	4.89
380	64337	Gng13	1636.88	4.89
381	67332	Snrpd3	2705.41	4.88
382	14726	Pdpn	987.54	4.87
383	11607	Agtr1a	868.26	4.87
384	237781	Mief2	2172.74	4.87
385	100043102	4632428C04Rik	978.23	4.87
386	50722	Dkkl1	756.83	4.86
387	399101	Snhg3	1955.52	4.86
388	30055	Timm13	2502.13	4.86
389	50498	Ebi3	908.46	4.85
390	66276	1810009A15Rik	2126.12	4.85
391	20364	Selenow	3953.52	4.85
392	320014	B930025P03Rik	1049.12	4.84
393	109901	Cela1	725.21	4.84
394	68202	Ndufa5	5136.64	4.83
395	73721	1110017D15Rik	1242.64	4.83
396	381347	4930412O13Rik	729.14	4.83
397	321019	Gpr183	596.46	4.83
398	76785	2410124H12Rik	344.98	4.83
399	545261	Bvht	1384.91	4.83
400	101966	D8Ertd738e	3095.88	4.82
401	19301	Pxmp2	747.92	4.80
402	78369	Icam4	938.29	4.80
403	66156	Anapc11	4260.24	4.79
404	11807	Apoa2	794.13	4.79
405	66060	Cystm1	5369.42	4.79
406	17910	Myo15	1061.67	4.79
407	67676	Rpp21	3748.33	4.79
408	69038	Tmem258	2735.42	4.78
409	66448	Mrpl20	3444.37	4.78
410	11853	Rhoc	1554.96	4.78
411	67212	Mrpl55	3200.65	4.77
412	114143	Atp6v0b	4080.64	4.77

1141	19395	Rasgrp2	1422.86	3.42
1142	56086	Set	8397.52	3.42
1143	59010	Sqrdl	1167.19	3.42
1144	67569	Mgat4c	1549.56	3.42
1145	18424	Otx2	735.99	3.42
1146	66653	Brf2	1048.48	3.42
1147	78445	C330013E15Rik	614.57	3.41
1148	59042	Cope	2401.69	3.41
1149	23831	Car14	696.57	3.41
1150	20196	S100a13	667.98	3.41
1151	68918	1190005I06Rik	513.41	3.41
1152	69895	Snhg8	1308.66	3.41
1153	66148	Dnajc15	1766.31	3.41
1154	73293	Ccdc103	502.08	3.41
1155	16763	Lad1	551.53	3.41
1156	50773	Nt5c	1743.92	3.40
1157	100504703	A730063M14Rik	510.79	3.40
1158	232910	Ap2s1	1631.80	3.40
1159	100503337	Gm9895	249.94	3.40
1160	433771	Minos1	3160.42	3.40
1161	12039	Bckdha	1068.94	3.40
1162	20316	Sdf2	1785.00	3.40
1163	227659	Slc2a6	1003.14	3.40
1164	117592	B3galt6	2198.29	3.40
1165	93843	Pnck	2410.47	3.40
1166	27973	Vkorc1	1734.74	3.40
1167	18168	Npy5r	587.58	3.40
1168	13090	Cyp2b19	667.24	3.39
1169	74485	Lrrc71	428.73	3.39
1170	68043	Eef1akmt1	1384.88	3.39
1171	67689	Aldh3b1	655.46	3.39
1172	68222	Fam166a	362.93	3.39
1173	56040	Rplp1	1749.09	3.39
1174	68512	Tomm5	2976.94	3.39
1175	232337	Zfp637	1471.59	3.39
1176	66495	Ndufb3	2285.06	3.39
1177	56295	Higd1a	3378.63	3.38
1178	100503311	Pifo	380.67	3.38
1179	75291	Zbtb3	753.74	3.38
1180	22092	Rsph1	941.31	3.38
1181	70426	Tekt5	1200.57	3.38
1182	66125	Sf3b5	1308.80	3.38
1183	230822	Ncmap	615.61	3.38
1184	228942	Cbln4	1437.87	3.37
1185	68991	Ssu72	3354.54	3.37
1186	227290	Aamp	2757.72	3.37
1187	50933	Uchl3	2210.19	3.37
1188	68499	Mrpl53	2154.32	3.37
1189	107733	Mrpl41	2060.31	3.36
1190	69535	Ten1	1908.69	3.36
1191	72093	2010320M18Rik	657.50	3.36
1192	212898	Dse	812.38	3.36
1193	20918	Eif1	5207.30	3.36
1194	214253	Etnk2	642.56	3.36
1195	66401	Nudt2	1462.57	3.35
1196	18633	Pex16	1289.23	3.35
1197	94065	Mrpl34	2266.74	3.35
1198	14910	Gt(ROSA)26Sor	814.17	3.35
1199	117160	Ttyh2	1902.74	3.35
1200	12941	Pcdha5	534.91	3.35
1201	66838	0610009L18Rik	559.27	3.35
1202	66844	Ormdl2	1198.85	3.35
1203	22122	Tsta3	1253.97	3.35
1204	66128	Mrps36	2305.83	3.35
1205	69519	Rwdd2a	2834.03	3.35
1206	140486	Igf2bp1	445.29	3.35
1207	66420	Polr2e	1368.24	3.35
1208	11595	Acan	496.79	3.35
1209	21877	Tk1	628.11	3.34

413	278097	Armcx6	1722.53	4.76
414	320315	A330048O09Rik	641.72	4.76
415	66447	Mgst3	3820.59	4.75
416	18476	Pafah1b3	3467.84	4.75
417	78312	1810041H14Rik	614.86	4.75
418	30045	Dnajc12	2535.29	4.74
419	224904	2410015M20Rik	2534.85	4.74
420	100037283	Rnaset2a	173.42	4.74
421	100040972	Tceal7	728.05	4.74
422	230075	Ndufb6	3521.68	4.74
423	11936	Fxyd2	1159.45	4.73
424	103988	Gck	1008.35	4.73
425	110257	Hba-a2	1880.75	4.73
426	380840	Lyrm4	4957.48	4.73
427	20832	Ssr4	2828.62	4.72
428	74264	Rnf138rt1	807.90	4.72
429	27279	Tnfrsf12a	1091.72	4.71
430	208501	Ndufaf8	2266.35	4.71
431	214305	Hhipl1	1122.97	4.71
432	114668	5033404E19Rik	402.15	4.70
433	433700	Spag8	839.47	4.70
434	76668	Mdh1b	672.66	4.70
435	66320	Tmem208	2643.03	4.70
436	66437	Fis1	3998.58	4.69
437	20304	Ccl5	98.01	4.69
438	112418	1700102P08Rik	752.02	4.69
439	69441	1700023F06Rik	1455.14	4.68
440	74153	Uba7	561.98	4.68
441	78444	Pgpep1l	463.36	4.68
442	319197	Gpr4	1642.89	4.67
443	100039707	Mthfsl	3389.27	4.67
444	18618	Pemt	1133.58	4.67
445	11811	Apobec2	545.34	4.67
446	68611	Mrpl28	2859.51	4.66
447	66212	Sec61b	2341.79	4.66
448	100034739	Gm17762	1276.38	4.65
449	217149	Cisd3	3630.62	4.65
450	78073	6720468P15Rik	676.98	4.64
451	102639259	Gm35612	927.79	4.64
452	15192	Hdgfl1	441.06	4.64
453	80879	Slc16a3	641.38	4.64
454	68371	Pbld1	1210.33	4.63
455	66119	Tomm6	4526.27	4.63
456	22187	Ubb	4430.69	4.62
457	105734734	Gm11715	164.24	4.62
458	330577	Saxo2	2558.08	4.62
459	57423	Atp5j2	3808.36	4.61
460	83409	Lamtor2	3326.94	4.61
461	68002	Sdhaf4	2760.23	4.61
462	12555	Cdh15	1039.50	4.61
463	17991	Ndufa2	3079.91	4.61
464	57320	Park7	2949.51	4.60
465	13611	S1pr4	875.23	4.59
466	214917	Fam173a	3118.45	4.59
467	66121	Chchd1	2472.98	4.58
468	68463	Mrpl14	2013.57	4.58
469	57780	Fxyd7	2124.04	4.58
470	66477	Usmg5	3016.04	4.57
471	14870	Gstp1	2522.75	4.57
472	66169	Tomm7	3420.46	4.57
473	104130	Ndufb11	4399.25	4.57
474	78709	Spink8	848.56	4.56
475	23961	Oas1b	796.36	4.56
476	53895	Clpp	2766.27	4.55
477	75472	Cfap126	971.49	4.55
478	433099	Ly6g6f	317.90	4.54
479	104522	AU040972	538.58	4.54
480	228778	6820408C15Rik	1353.85	4.54
481	16818	Lck	882.86	4.54

1210	72185	Dbndd1	1687.68	3.34
1211	382245	Tmem29	2022.30	3.34
1212	70316	Ndufab1	2663.43	3.34
1213	60315	Myg1	1771.43	3.34
1214	235505	Cd109	482.81	3.33
1215	66151	Prr13	3634.82	3.33
1216	71817	Tmem50a	4103.05	3.33
1217	72481	2610203C22Rik	309.95	3.33
1218	69269	Scnm1	1884.79	3.33
1219	11858	Rnd2	1664.37	3.33
1220	212555	Pqlc2	1146.03	3.33
1221	108841	Rdh13	1903.70	3.33
1222	58237	Nkain4	1070.87	3.33
1223	116849	Iltifb	305.41	3.33
1224	100061	Lrrc19	408.51	3.33
1225	57330	Gigyf1	1752.56	3.33
1226	104885	Tmem179	2099.12	3.33
1227	21855	Timm17b	1723.65	3.32
1228	19716	Bex1	2982.36	3.32
1229	78801	Ak7	545.05	3.32
1230	76846	Rps9	2167.58	3.32
1231	330830	Drc7	432.97	3.31
1232	14651	Hagh	2190.37	3.31
1233	67091	Trappc6a	870.35	3.31
1234	22342	Lin7b	2370.42	3.31
1235	212123	Dcaf15	1050.50	3.31
1236	110595	Timp4	1052.88	3.31
1237	20813	Srp14	1917.99	3.31
1238	66176	Nat9	1104.09	3.31
1239	67674	Trmt112	1986.39	3.31
1240	14775	Gpx1	1638.02	3.31
1241	244923	Klhl31	1342.49	3.31
1242	140781	Myh7	1390.59	3.30
1243	320736	Vstm4	478.73	3.30
1244	22273	Uqcrc1	3495.46	3.30
1245	215814	Ccdc28a	1529.92	3.30
1246	66576	Uqcrh	2204.60	3.30
1247	19035	Ppib	1557.49	3.30
1248	237362	Npffr1	344.16	3.30
1249	214531	Tmprss13	887.28	3.30
1250	102640845	Gm26994	241.48	3.30
1251	26961	Rpl8	1746.63	3.30
1252	110033	Kif22	865.22	3.29
1253	13849	Ephx1	974.93	3.29
1254	66399	Tsfm	1803.28	3.29
1255	102791	Tcta	1603.68	3.29
1256	75579	2310034G01Rik	403.60	3.29
1257	104367	Snora65	324.73	3.29
1258	52637	Cisd1	2558.90	3.29
1259	331374	Dgkk	1718.90	3.29
1260	17766	Nudt1	878.01	3.29
1261	83961	Nrg4	540.33	3.29
1262	77254	Yif1b	1100.53	3.29
1263	22038	Plscr1	488.31	3.29
1264	66177	Ubl5	2160.50	3.29
1265	100418235	C130030K03Rik	870.32	3.29
1266	20193	S100a1	1220.27	3.29
1267	69071	Tmem97	1155.41	3.29
1268	193217	BC018473	660.70	3.29
1269	73711	Mvb12a	1353.53	3.28
1270	76252	Atp6v0e2	2922.48	3.28
1271	246707	Emilin2	496.47	3.28
1272	67112	Fgf22	494.83	3.28
1273	244885	Sh2d7	1199.91	3.28
1274	73906	4833417C18Rik	613.22	3.28
1275	215210	Tmem120a	696.73	3.28
1276	50873	Park2	1787.62	3.28
1277	252974	Tspear	307.60	3.28
1278	27425	Atp5l	1766.57	3.28

482	14570	Arhgdig	2668.83	4.54
483	20643	Snrpe	2602.47	4.54
484	60409	Trappc4	4678.77	4.54
485	68176	Fam212a	876.25	4.53
486	67735	4930528A17Rik	866.10	4.53
487	319859	E030011O05Rik	610.98	4.53
488	74165	Fbxl22	412.31	4.53
489	67673	Elob	3295.49	4.53
490	380773	Slirp	4013.02	4.53
491	104362	Meig1	987.36	4.52
492	68396	Nat8	379.74	4.52
493	14470	Rabac1	2236.45	4.52
494	74890	Morn3	821.76	4.52
495	100502745	2900009J06Rik	764.18	4.52
496	99542	AI115009	648.89	4.51
497	353502	Hcfc1r1	3340.37	4.51
498	70726	Angptl6	883.36	4.51
499	66094	Lsm7	2921.98	4.50
500	74454	4933408J17Rik	922.51	4.50
501	30954	Siva1	2474.34	4.50
502	19175	Psmb6	3973.22	4.49
503	613254	AA465934	978.28	4.49
504	69770	1600002K03Rik	952.76	4.49
505	68198	Ndufb2	2409.84	4.49
506	14724	Gp1bb	1462.57	4.48
507	66913	Kdelr2	6851.68	4.47
508	100504477	Gm16677	733.47	4.47
509	66387	Nudt8	1645.68	4.47
510	72658	2700097O09Rik	2369.75	4.47
511	71200	Dydc2	1027.66	4.47
512	232987	B9d2	2541.64	4.47
513	213002	Ifitm6	449.58	4.46
514	100040294	Gm2694	1741.13	4.46
515	69432	1700026J14Rik	349.06	4.46
516	78330	Ndufv3	3367.87	4.46
517	68735	Mrps18c	3296.58	4.46
518	75124	Nxnl2	227.60	4.46
519	68090	Yif1a	1796.30	4.45
520	66242	Mrps16	2635.09	4.45
521	20669	Sox14	358.79	4.45
522	26446	Psmb3	3869.13	4.45
523	22381	Tceal9	4331.38	4.44
524	70575	Gfod2	2206.35	4.44
525	66162	Bola2	2203.82	4.44
526	24110	Usp18	742.02	4.43
527	70967	Eva1c	1513.60	4.43
528	75564	Rsph9	1261.30	4.43
529	407785	Ndufs6	2851.66	4.42
530	216227	Slc17a8	1877.94	4.42
531	80795	Selenok	4015.16	4.42
532	72748	Hdhd3	1293.86	4.41
533	30791	Slc39a1	2476.61	4.41
534	269854	Nat14	2003.77	4.40
535	20085	Rps19	2471.21	4.40
536	257635	Sdsl	712.73	4.40
537	66297	Pantr1	2327.24	4.39
538	70162	Hk1os	749.09	4.39
539	100038514	Gm11837	707.08	4.39
540	77803	Fam159b	831.67	4.39
541	56368	Cyb561d2	1367.82	4.39
542	243881	Cyp2b23	475.58	4.38
543	320846	A530058N18Rik	2069.27	4.38
544	104080	Nxph4	1532.25	4.38
545	72058	Igsf5	855.80	4.38
546	69159	Rhebl1	1198.33	4.38
547	69692	Hddc2	2865.21	4.37
548	98256	Kmo	378.20	4.37
549	353156	Egfl7	1573.32	4.37
550	66414	Ndufa12	4374.67	4.37

1279	56360	Acot9	1662.32	3.28
1280	67934	1700124L16Rik	429.78	3.28
1281	70450	Unc13d	907.74	3.27
1282	65019	Rpl23	2266.35	3.27
1283	402757	9830166K06Rik	660.28	3.27
1284	27207	Rps11	2449.37	3.27
1285	69749	Epb41l4aos	828.06	3.27
1286	12642	Ch25h	300.78	3.27
1287	72774	Neil1	518.60	3.27
1288	22223	Uchl1	1955.29	3.26
1289	14872	Gstt2	783.28	3.26
1290	244958	Mrap2	1570.62	3.26
1291	107993	Bfsp2	466.97	3.26
1292	17904	Myl6	2437.09	3.26
1293	66734	Map1lc3a	2295.64	3.25
1294	20021	Polr2c	3593.13	3.25
1295	22186	Uba52	1726.03	3.25
1296	52700	Txndc17	2629.76	3.25
1297	224613	Flywch1	2202.78	3.25
1298	16592	Fabp5	2432.03	3.25
1299	94242	Tinagl1	320.34	3.25
1300	237730	Fbll1	1303.39	3.25
1301	70134	2210011C24Rik	313.34	3.25
1302	69094	Tmem160	1180.17	3.25
1303	50799	Slc25a13	687.63	3.24
1304	66222	Serpinb1a	802.20	3.24
1305	68044	Chac2	1328.16	3.24
1306	53328	Pgrmc1	2786.39	3.24
1307	20686	Spa17	1173.80	3.24
1308	240327	Gm4951	921.72	3.24
1309	67341	Ascl4	796.09	3.24
1310	66230	Mrps28	1177.76	3.24
1311	72017	Cyb5r1	2055.61	3.24
1312	320333	D830030K20Rik	1314.90	3.24
1313	100041290	D4Ertd617e	443.37	3.24
1314	56529	Sec11a	1544.58	3.23
1315	100503915	Smpd5	179.80	3.23
1316	66199	Commd4	1515.92	3.23
1317	217653	Mis18bp1	557.88	3.23
1318	11769	Ap1s1	1377.16	3.23
1319	27050	Rps3	2128.46	3.23
1320	66462	2810428I15Rik	1358.58	3.23
1321	102566	Ano10	1405.91	3.23
1322	12576	Cdkn1b	2064.80	3.23
1323	56433	Vps29	3570.89	3.23
1324	211378	6720489N17Rik	1122.09	3.23
1325	100169889	Peg3os	807.55	3.22
1326	67210	Gatad1	6346.65	3.22
1327	67106	Zbtb8os	1506.03	3.22
1328	14297	Fxn	1594.98	3.22
1329	19286	Pts	2347.79	3.22
1330	70103	Znhit1	1268.66	3.22
1331	21854	Timm17a	3712.27	3.22
1332	330173	2610524H06Rik	1519.01	3.22
1333	78004	Prr15	971.92	3.22
1334	56455	Dynll1	2387.94	3.22
1335	14313	Fst	692.61	3.22
1336	100040671	Gm2897	662.35	3.22
1337	66111	Tmed3	1343.41	3.22
1338	67304	3110070M22Rik	607.86	3.22
1339	217707	Coq6	998.31	3.21
1340	20513	Slc1a6	1401.36	3.21
1341	69784	1500009L16Rik	1951.18	3.21
1342	269683	E130006D01Rik	445.31	3.21
1343	27414	Sergef	1307.03	3.21
1344	17083	Tmed1	851.83	3.21
1345	208092	Chmp6	1180.81	3.21
1346	54399	Bet1l	2095.77	3.20
1347	12306	Anxa2	1035.32	3.20

551	12922	Crhr2	571.63	4.37
552	67752	Ppp1r32	505.20	4.36
553	75881	4930579K19Rik	646.80	4.36
554	100502742	1700007L15Rik	662.54	4.36
555	20335	Sec61g	2652.45	4.36
556	67184	Ndufa13	3437.39	4.36
557	381077	Ccdc78	970.92	4.35
558	72648	Smc2os	395.68	4.35
559	14356	Timm10b	2874.66	4.35
560	208990	Npb	587.61	4.34
561	70536	Qpct	2083.69	4.34
562	71141	4933407L21Rik	956.70	4.34
563	66059	Krtcap2	2023.88	4.34
564	50721	Sirt6	3309.92	4.33
565	93835	Amn	974.20	4.33
566	77741	6720483E21Rik	454.41	4.33
567	75556	Cfap161	823.51	4.32
568	72027	Slc39a4	230.73	4.32
569	625018	C4a	558.69	4.32
570	100038570	Prcd	1071.80	4.31
571	69537	Dnase1l1	729.01	4.31
572	12491	Cd36	618.28	4.30
573	66449	Pam16	2414.51	4.30
574	654494	Gm7337	1592.95	4.30
575	14778	Gpx3	2161.13	4.30
576	21645	Tcte1	769.40	4.30
577	69097	Trim15	573.67	4.29
578	80284	Smim12	2431.02	4.29
579	434784	Ldoc1	952.17	4.29
580	69804	Tmem147	2461.44	4.28
581	105844	Card10	1256.12	4.28
582	67224	Med29	1984.40	4.28
583	56327	Arl2	3417.02	4.28
584	110323	Cox6b1	3605.88	4.28
585	97159	A430005L14Rik	2820.57	4.27
586	100038654	Gm10371	1047.96	4.26
587	72632	Smim18	1973.85	4.26
588	69920	Polr2i	2440.25	4.26
589	68337	Crip2	2064.86	4.25
590	11636	Ak1	3479.84	4.25
591	69126	1810022K09Rik	2529.40	4.25
592	666744	Gm8267	460.89	4.25
593	76497	Ppp1r11	3237.62	4.24
594	68603	Pmvk	3241.54	4.24
595	14028	Evx1	83.10	4.24
596	18081	Ninj1	1778.22	4.23
597	244810	AW551984	2424.93	4.23
598	17319	Mif	2253.26	4.23
599	69547	Nkpd1	614.81	4.23
600	67289	3110021A11Rik	223.97	4.23
601	18155	Pnoc	1501.94	4.23
602	67941	Rps27l	2112.05	4.23
603	18976	Pomc	1208.50	4.23
604	66118	Sarnp	4267.38	4.22
605	74097	Pop7	1957.50	4.22
606	100042757	Gm4013	618.01	4.22
607	69019	Spcs1	3540.31	4.22
608	21648	Dynlt1b	1869.17	4.22
609	69928	Apitd1	1436.95	4.22
610	15468	Prmt2	3186.22	4.21
611	110196	Fdps	2794.57	4.21
612	59091	Jph2	543.76	4.21
613	18102	Nme1	4678.91	4.21
614	76972	Snhg20	1013.50	4.21
615	68162	A930003A15Rik	414.93	4.21
616	69315	1700001L19Rik	2398.15	4.21
617	19200	Pstpip1	562.48	4.21
618	12417	Cbx3	6038.37	4.21
619	68995	Mcts1	3897.36	4.21

1348	20420	Shd	1277.41	3.20
1349	66143	Eef1e1	2190.25	3.20
1350	76916	Timmdc1	3231.97	3.20
1351	654822	D330041H03Rik	1101.79	3.20
1352	76302	Pcnp	3644.37	3.20
1353	75304	4930563E22Rik	639.21	3.20
1354	27370	Rps26	1569.93	3.19
1355	21331	T2	856.35	3.19
1356	19989	Rpl7	1911.66	3.19
1357	622404	Ccdc107	996.39	3.19
1358	66245	Hspbp1	1242.64	3.19
1359	26893	Cops6	3299.54	3.19
1360	11812	Apoc1	259.28	3.19
1361	69478	2300009A05Rik	1119.20	3.19
1362	78245	Acbd7	548.04	3.19
1363	54200	Sult2b1	739.41	3.19
1364	14300	Frg1	1721.28	3.18
1365	20974	Syngr3	2383.53	3.18
1366	78267	Klhdc8b	1347.38	3.18
1367	15388	Hnrnpl	1728.68	3.18
1368	74319	Mettl23	980.72	3.18
1369	80893	Tmprss5	261.33	3.18
1370	57808	Rpl35a	1641.15	3.18
1371	20254	Scg2	2659.36	3.18
1372	381104	Prickle4	532.35	3.18
1373	67186	Rplp2	1317.83	3.18
1374	69163	Mrpl44	2135.06	3.18
1375	26445	Psmb2	2346.48	3.17
1376	434756	Akap14	334.28	3.17
1377	100041286	Snhg15	878.19	3.17
1378	94045	P2rx5	400.92	3.17
1379	67681	Mrpl18	3618.92	3.17
1380	52443	Mrpl48	2316.21	3.17
1381	70025	Acot7	3766.45	3.17
1382	66467	Gtf2h5	2574.11	3.17
1383	627191	Syndig1l	1918.76	3.17
1384	71846	Syce2	665.71	3.17
1385	75533	Nme5	1101.19	3.17
1386	78651	Lsm6	3567.31	3.17
1387	21410	Hnf1b	467.71	3.17
1388	64293	Stk32b	1555.18	3.17
1389	107250	Kazald1	579.66	3.17
1390	22746	Zfp85	988.65	3.16
1391	18142	Npas1	925.36	3.16
1392	78329	2310010J17Rik	607.06	3.16
1393	100764	Rita1	1006.59	3.16
1394	103742	Mien1	1556.07	3.16
1395	230558	C8a	386.33	3.16
1396	195646	Hs3st2	1178.12	3.16
1397	69713	Pin4	1656.60	3.16
1398	68944	Tmco1	2731.96	3.16
1399	12652	Chga	2070.42	3.16
1400	66077	Aurkaip1	2128.07	3.16
1401	16977	Lrrc23	573.49	3.16
1402	97961	Nol12	1461.06	3.16
1403	12556	Cdh16	603.53	3.15
1404	216873	Spag7	1927.03	3.15
1405	14705	Bscl2	2354.85	3.15
1406	20347	Sema3b	650.37	3.15
1407	22192	Ube2m	2268.46	3.15
1408	14004	Chchd2	2123.96	3.15
1409	72931	Swi5	1539.50	3.15
1410	18127	Nos3	616.07	3.15
1411	14923	Guk1	3554.88	3.15
1412	192173	Fam195b	1626.07	3.15
1413	56486	Gabarap	3215.98	3.15
1414	30953	Schip1	2932.35	3.15
1415	76478	Haus8	917.10	3.15
1416	66340	Psenen	2119.32	3.15

620	71233	Enkur	906.52	4.20
621	69723	Rpain	1510.13	4.20
622	19023	Ppef2	521.76	4.20
623	66286	Sec11c	2655.42	4.20
624	271375	Cd200r2	758.95	4.19
625	68554	Cebpzos	2565.24	4.19
626	18654	Pgf	729.96	4.19
627	231070	Insig1	5474.19	4.19
628	11983	Atpif1	3858.23	4.19
629	11927	Atox1	2126.35	4.19
630	69833	Polr2f	1910.26	4.19
631	23988	Pin1	3107.40	4.19
632	12925	Crip1	840.68	4.18
633	12335	Capn3	919.34	4.18
634	11423	Ache	2722.10	4.18
635	15254	Hint1	3652.74	4.18
636	16854	Lgals3	335.36	4.17
637	75860	Tex26	481.37	4.17
638	16372	Irx2	960.94	4.16
639	12512	Cd63	2764.49	4.16
640	114679	Selenom	2284.85	4.16
641	75406	Ndufs7	2561.50	4.16
642	239463	Fam83a	881.24	4.16
643	66096	Lamtor4	2066.17	4.16
644	17992	Ndufa4	3662.40	4.16
645	12015	Bad	1602.77	4.15
646	15006	H2-Q1	1317.79	4.15
647	16913	Psmb8	731.93	4.15
648	14867	Gstm6	1263.75	4.15
649	19935	Mrpl23	1800.78	4.15
650	114585	D17H6S53E	3179.18	4.15
651	11567	Avil	384.81	4.15
652	667410	Gm8615	479.26	4.15
653	20429	Shox2	1124.64	4.15
654	26945	Tpsg1	137.49	4.15
655	67971	Tppp3	2306.97	4.14
656	13386	Dlk1	2089.00	4.14
657	432396	Zfp652os	822.19	4.14
658	11555	Adrb2	904.98	4.14
659	67270	Mrpl42	6983.35	4.14
660	21689	Tekt1	887.98	4.14
661	246735	AY074887	1986.53	4.14
662	226162	Dpcd	3223.78	4.14
663	606735	A330069E16Rik	1597.18	4.14
664	26901	Ss18l2	4821.37	4.14
665	75568	Capsl	592.75	4.12
666	21349	Tal1	764.42	4.12
667	213350	Pddc1	2524.46	4.12
668	16399	Itga2b	1562.35	4.12
669	70274	Ly6g6e	749.15	4.12
670	78112	4930452N14Rik	846.35	4.12
671	66373	Lsm5	1539.43	4.12
672	85308	Emc9	3301.42	4.12
673	12362	Casp1	685.68	4.12
674	14077	Fabp3	2855.40	4.12
675	18113	Nnmt	357.01	4.12
676	75857	4930568G15Rik	325.93	4.12
677	67693	Hypk	2920.06	4.11
678	170442	Bbox1	666.85	4.11
679	208171	Tmprss7	635.53	4.11
680	14991	H2-M3	839.49	4.11
681	246048	Chodl	985.88	4.11
682	75657	Speer4a	356.36	4.11
683	27412	Peg12	1014.76	4.10
684	19703	Renbp	579.16	4.10
685	77613	Prss36	1136.50	4.10
686	67994	Mrps11	2095.22	4.10
687	68671	Pcyt2	2734.76	4.09
688	66487	Smim4	4002.73	4.09

1417	67217	L3hypdh	957.50	3.15
1418	52466	Slc46a1	1067.85	3.15
1419	18733	Pirb	172.20	3.14
1420	78294	Rps27a	1644.82	3.14
1421	100041874	Gm3558	1154.20	3.14
1422	74686	Slc25a54	190.56	3.14
1423	14693	Gnb2	1668.22	3.14
1424	13590	Lefty1	207.79	3.14
1425	17035	Lxn	1748.62	3.13
1426	69731	Gemin7	1050.79	3.13
1427	474332	Dnm3os	812.30	3.13
1428	93696	Chrac1	1413.09	3.13
1429	229801	Tram1l1	1581.03	3.13
1430	67389	Fam132a	858.20	3.13
1431	71186	4933417D19Rik	367.99	3.13
1432	59031	Chst12	1050.61	3.13
1433	12861	Cox6a1	2256.92	3.13
1434	66971	Cdk5rap1	913.21	3.12
1435	66706	Ndufaf3	1398.42	3.12
1436	231871	Daglb	1594.51	3.12
1437	271424	Ip6k3	188.83	3.12
1438	74137	Nuak2	581.83	3.12
1439	29862	Ninj2	657.28	3.12
1440	328263	A530065N20Rik	969.29	3.12
1441	67864	Yipf4	1272.69	3.12
1442	67937	Tmem59l	1705.77	3.12
1443	67942	Atp5g2	1649.72	3.11
1444	71787	Trnau1ap	968.41	3.11
1445	72355	Cdpf1	1470.11	3.11
1446	20641	Snrpd1	2341.39	3.11
1447	11984	Atp6v0c	2316.87	3.11
1448	14432	Gap43	4007.09	3.11
1449	66375	Dhrs7	999.83	3.11
1450	74846	4930405A21Rik	498.94	3.11
1451	233065	Alkbh6	1624.82	3.11
1452	100504641	1700101I11Rik	491.42	3.11
1453	100503890	Pet100	1675.55	3.11
1454	14585	Gfra1	1554.15	3.11
1455	320842	C230035I16Rik	284.07	3.10
1456	18100	Mrpl40	1408.53	3.10
1457	68949	Zfas1	1442.11	3.10
1458	69263	Rfc3	1172.71	3.10
1459	14226	Fkbp1b	2154.92	3.10
1460	110826	Etfb	1146.42	3.10
1461	57890	Il17re	1015.23	3.10
1462	20170	Hps6	943.26	3.10
1463	13639	Efna4	189.03	3.10
1464	240479	Fam69c	593.28	3.09
1465	15901	Id1	593.75	3.09
1466	234023	Arglu1	1958.48	3.09
1467	244608	Ccdc113	581.51	3.09
1468	22040	Trex1	680.66	3.09
1469	66481	Rps21	1493.28	3.09
1470	69225	Naxd	1035.51	3.09
1471	320802	Ifitm10	2297.13	3.09
1472	67170	2610306M01Rik	736.98	3.09
1473	102060	Gadd45gip1	1402.86	3.09
1474	14635	Galk1	903.45	3.08
1475	100503636	1700123M08Rik	799.60	3.08
1476	237411	Zfp938	2150.62	3.08
1477	69028	Mitd1	1472.70	3.08
1478	216853	Wrap53	771.99	3.08
1479	100502630	Gm15545	641.36	3.08
1480	14618	Gjb1	740.01	3.08
1481	75538	Fam71e1	743.48	3.08
1482	67678	Lsm3	1229.83	3.08
1483	13214	Defb1	261.50	3.08
1484	14871	Gstt1	916.84	3.08
1485	208211	Alg1	844.97	3.08

689	665433	Hist1h2ao	621.69	4.09
690	71146	Golga7b	3072.56	4.09
691	75454	Phpt1	2872.91	4.08
692	66152	Uqcr10	2343.98	4.08
693	14198	Fhit	907.81	4.08
694	55932	Gbp3	510.77	4.08
695	66910	Tmem107	1307.97	4.08
696	69156	Comtd1	1415.25	4.07
697	225887	Ndufs8	2495.85	4.07
698	11459	Acta1	1238.01	4.07
699	12521	Cd82	622.42	4.07
700	217011	Nle1	862.68	4.07
701	211468	Kcnh8	790.52	4.06
702	54218	B3galt4	863.74	4.06
703	112422	Zfp979	974.81	4.06
704	192970	Dhrs11	755.32	4.06
705	74973	4930500L23Rik	680.98	4.06
706	17075	Epcam	275.65	4.06
707	18969	Pola2	2309.30	4.06
708	28080	Atp5o	3122.61	4.06
709	217203	Tmem106a	622.08	4.06
710	407790	Ndufa4l2	695.42	4.05
711	67067	Romo1	1885.29	4.05
712	73712	Dmkn	498.55	4.05
713	72900	Ndufv2	5995.02	4.05
714	11958	Atp5k	2766.25	4.05
715	74230	1700016K19Rik	791.15	4.05
716	69694	Tatdn1	4046.25	4.05
717	13389	Dll3	1022.06	4.04
718	66469	Fam213b	2446.37	4.04
719	231646	Myo1h	636.08	4.03
720	245827	Fat2	616.76	4.03
721	17299	Mettl1	1348.31	4.03
722	68342	Ndufb10	3581.67	4.02
723	70113	Odf3b	809.99	4.02
724	19171	Psmb10	2606.61	4.02
725	70448	Atad3aos	784.67	4.02
726	330305	Gm5111	330.01	4.02
727	100043040	1110002L01Rik	1149.86	4.02
728	282663	Serpinb1b	1113.30	4.02
729	66973	Mrps18b	2300.52	4.02
730	67877	Naa20	6206.82	4.02
731	75116	4930520O04Rik	670.52	4.01
732	99650	4933434E20Rik	5481.54	4.01
733	381792	2310040G24Rik	610.36	4.01
734	233016	Blvrb	1312.10	4.01
735	11461	Actb	9467.99	4.01
736	66836	Tmem223	2573.65	4.01
737	68566	Caly	2484.26	4.01
738	67313	5730559C18Rik	569.28	4.01
739	19240	Tmsb10	3036.77	4.00
740	20022	Polr2j	1681.22	4.00
741	73545	1700094D03Rik	1677.13	4.00
742	72832	Crtac1	2724.18	4.00
743	223267	Ggact	2732.42	4.00
744	75404	Arhgap36	1420.02	4.00
745	15374	Hn1	1737.62	4.00
746	14976	Pfdn6	2394.34	3.99
747	19288	Ptx3	697.80	3.99
748	16373	Irx3	732.19	3.98
749	56856	Insm2	452.79	3.98
750	80285	Parp9	752.39	3.98
751	21355	Tap2	634.27	3.98
752	12866	Cox7a2	2687.41	3.98
753	23923	Aadat	295.68	3.98
754	22044	Trh	711.55	3.98
755	107686	Snrpd2	2119.11	3.97
756	14029	Evx2	361.20	3.96
757	13135	Dad1	2958.71	3.96
1486	72096	Mettl10	1983.85	3.08
1487	66959	Dusp26	2708.62	3.08
1488	68920	1110065P20Rik	877.13	3.08
1489	112419	Ifit1bl2	670.74	3.08
1490	20646	Snrpn	1035.60	3.08
1491	20630	Snrpc	1382.45	3.08
1492	20922	Supt4a	1471.28	3.08
1493	56292	Naa10	1342.30	3.07
1494	66506	Psmg3	1192.62	3.07
1495	71893	Noxo1	524.76	3.07
1496	66384	Srp19	1917.32	3.07
1497	11690	Alox5ap	600.96	3.07
1498	66317	Wdr61	2106.95	3.07
1499	735274	Mir684-1	1245.22	3.07
1500	13202	Ddt	1414.30	3.07
1501	67399	Pdlim7	1100.17	3.07
1502	66042	Sostdc1	509.28	3.07
1503	227746	Rabepk	1620.39	3.07
1504	66234	Msmo1	3043.46	3.07
1505	20103	Rps5	1454.83	3.07
1506	18477	Prdx1	3164.46	3.07
1507	14612	Gja4	348.06	3.07
1508	64657	Mrps10	2155.33	3.07
1509	74316	Isca2	2214.82	3.07
1510	11520	Plin2	749.99	3.06
1511	59047	Pnkp	766.60	3.06
1512	30840	Fbxl6	746.69	3.06
1513	100322896	Dthd1	110.75	3.06
1514	76467	Msrb2	1501.81	3.06
1515	73648	2410012E07Rik	384.56	3.06
1516	13870	Ercc1	790.20	3.06
1517	12869	Cox8b	274.29	3.06
1518	625638	Fam43b	1279.55	3.06
1519	73723	Sh3bgrl3	1899.99	3.06
1520	328451	Gm5088	483.98	3.06
1521	97402	C86187	282.71	3.05
1522	226422	Rab29	612.63	3.05
1523	78617	Cstad	507.64	3.05
1524	66273	Aamdc	1980.42	3.05
1525	14371	Fzd9	430.68	3.05
1526	386612	Thoc6	679.52	3.05
1527	18552	Pcsk5	896.78	3.05
1528	13010	Cst3	1845.99	3.04
1529	71803	Slc25a18	919.18	3.04
1530	108653	Rimklb	1208.64	3.04
1531	68118	Atg101	1149.52	3.04
1532	68520	Zfyve21	794.53	3.04
1533	20604	Sst	2440.23	3.04
1534	13008	Csrp2	633.57	3.04
1535	15081	H3f3b	2199.75	3.04
1536	11792	Apex1	1498.60	3.04
1537	69149	Kbtbd3	1161.15	3.04
1538	66493	Mrpl51	2857.40	3.04
1539	108687	Edem2	786.37	3.04
1540	12484	Cd24a	1530.05	3.03
1541	56534	Hspb3	285.38	3.03
1542	76959	Chmp5	2258.54	3.03
1543	66995	Zcchc18	2963.36	3.03
1544	60345	Nrip2	1979.84	3.03
1545	228715	Smim26	1356.05	3.03
1546	12319	Car8	809.85	3.03
1547	545641	Gm5860	371.23	3.03
1548	50776	Polg2	910.02	3.03
1549	67178	Zmat5	1826.82	3.03
1550	68323	Nudt22	791.31	3.03
1551	105278	Cdk20	756.68	3.03
1552	75597	Ndufaf2	1669.77	3.03
1553	75424	Zfp820	722.70	3.03
1554	71864	Fam217a	389.01	3.03

758	66154	Tmem14c	2645.30	3.96
759	66845	Mrpl33	2655.00	3.96
760	66292	Mrps21	2668.91	3.95
761	14912	Nkx6-2	1109.69	3.95
762	230971	Megf6	614.87	3.95
763	66268	Pigyl	1761.55	3.95
764	69066	1810010H24Rik	959.58	3.95
765	55951	Mpc1	4957.41	3.95
766	23962	Oasl2	1348.38	3.94
767	69386	Hist1h4h	760.10	3.94
768	68436	Rpl34	2645.90	3.94
769	216767	Mrpl22	2300.98	3.94
770	18103	Nme2	2345.40	3.94
771	66098	Chchd6	1505.58	3.93
772	28106	Mydgf	1630.68	3.93
773	67680	Sdhb	4890.57	3.93
774	621580	Gm13308	987.54	3.93
775	20459	Ptk6	1131.22	3.93
776	279766	Rhbdd3	1273.31	3.92
777	239133	Dleu7	2161.06	3.92
778	73720	Cst6	2172.00	3.92
779	93739	Gabarapl2	3116.30	3.91
780	66489	Rpl35	2251.07	3.91
781	65255	Asb4	1373.38	3.91
782	64095	Gpr35	820.47	3.91
783	70957	Stamos	417.36	3.91
784	66377	Ndufc1	3038.92	3.91
785	72614	Pih1d2	788.81	3.90
786	211151	Churc1	2875.26	3.90
787	100504309	Toporsos	990.67	3.90
788	68667	Trpm4	1151.80	3.90
789	64242	Ngb	1158.03	3.90
790	17000	Ltbr	616.11	3.90
791	15904	Id4	2517.64	3.89
792	67704	1810037I17Rik	3445.59	3.88
793	22262	Uox	311.18	3.88
794	12051	Bcl3	558.56	3.88
795	66126	Elof1	2122.61	3.88
796	66290	Atp6v1g1	4077.31	3.88
797	19170	Psmb1	4314.47	3.88

1555	68971	Tamm41	1103.41	3.03
1556	72391	Cdkn3	385.91	3.02
1557	219072	Haus4	871.55	3.02
1558	27419	Naglu	650.74	3.02
1559	66291	Smim8	1484.78	3.02
1560	69090	Ascc1	1075.50	3.02
1561	195040	Tmem199	1444.52	3.02
1562	24064	Spry2	2094.47	3.02
1563	53598	Dctn3	1303.99	3.02
1564	378937	Lrrc24	904.19	3.02
1565	229714	Gpr61	879.14	3.02
1566	11898	Ass1	1653.19	3.02
1567	109054	Pfdn4	1608.49	3.02
1568	59095	Fxyd6	1580.57	3.02
1569	13650	Rhbdf1	426.16	3.02
1570	69372	Mocs3	714.10	3.02
1571	224224	Impg2	486.63	3.02
1572	56551	Txn2	1979.93	3.02
1573	19173	Psmb5	2471.44	3.02
1574	51813	Ccnc	2277.46	3.02
1575	73689	Bloc1s2	1722.23	3.01
1576	69953	2810025M15Rik	1171.20	3.01
1577	70054	Ccdc89	510.57	3.01
1578	69221	2410006H16Rik	1201.46	3.01
1579	140483	Hnmt	1474.47	3.01
1580	433365	Teddm1b	1053.19	3.00
1581	71711	Mus81	914.16	3.00
1582	101122	Rpusd3	699.41	3.00
1583	227733	Pip5kl1	523.26	3.00
1584	240121	Fsd1	1491.57	3.00
1585	11950	Atp5f1	3692.54	3.00
1586	24068	Sra1	1333.55	3.00
1587	66058	Tmem176a	904.81	3.00
1588	56462	Mtch1	2352.17	2.99
1589	76688	Arfrp1	1758.18	2.99
1590	66441	Magohb	1150.36	2.99
1591	26426	Nubp2	1218.04	2.99
1592	20826	Snu13	3391.71	2.99
1593	76813	Armc6	1024.26	2.99
1594	73824	Snhg6	946.93	2.99

Supplementary Table 3: Molecular targets enriched in DRN Vglut3 neurons in relation to gene ontology pathways.

	IDs	Symbols	combined_chisq	mean_l2fc
1	226278	Prlhr	1740.37	12.35
2	19222	Ptgir	887.40	9.84
3	14399	Gabra6	1119.16	8.70
4	22296	Vmn1r51	458.03	8.43
5	71111	Gpr39	670.64	7.21
6	140498	Rxfp2	1497.22	7.13
7	12311	Calcr	1783.04	7.07
8	16178	Il1r2	767.96	6.60
9	230777	Hcrtr1	1234.32	6.57
10	15564	Htr5b	1231.45	6.15
11	14130	Fcgr2b	905.92	5.47
12	30044	Opn4	542.88	5.24
13	208188	Ghsr	1199.84	5.21
14	236781	Gpr119	57.98	5.07
15	12777	Ccr10	721.71	5.06
16	11607	Agtr1a	868.26	4.87
17	50498	Ebi3	908.46	4.85
18	321019	Gpr183	596.46	4.83
19	319197	Gpr4	1642.89	4.67
20	13611	S1pr4	875.23	4.59
21	12922	Crhr2	571.63	4.37
22	12491	Cd36	618.28	4.30
23	271375	Cd200r2	758.95	4.19
24	16854	Lgals3	335.36	4.17
25	11555	Adrb2	904.98	4.14
26	70274	Ly6g6e	749.15	4.12
27	64095	Gpr35	820.47	3.91
28	11556	Adrb3	606.53	3.82
29	13733	Adgre1	467.63	3.81
30	237313	Il20ra	321.98	3.73
31	213527	Pth2r	503.09	3.69
32	14427	Galr1	640.69	3.68
33	94043	Tm2d1	2211.57	3.66
34	15465	Hrh1	1067.84	3.58
35	67169	Nradd	317.90	3.49
36	18167	Npy2r	1194.40	3.47
37	11548	Adra1b	1123.07	3.47
38	18168	Npy5r	587.58	3.40
39	100061	Lrrc19	408.51	3.33
40	237362	Npffr1	344.16	3.30
41	14585	Gfra1	1554.15	3.11
42	57890	Il17re	1015.23	3.10
43	14371	Fzd9	430.68	3.05
44	229714	Gpr61	879.14	3.02
45	110304	Glra3	625.90	2.96
46	380732	Milr1	639.47	2.93
47	239336	Rxfp3	750.11	2.92
48	15550	Htr1a	1462.21	2.92
49	74090	Paqr5	546.06	2.90
50	15552	Htr1d	644.85	2.88
51	13983	Esr2	910.59	2.80
52	22337	Vdr	817.64	2.80
53	52710	Slc52a2	886.32	2.79
54	54409	Ramp2	677.18	2.78
55	17202	Mc4r	446.91	2.70
56	16157	Il11ra1	734.32	2.70
57	69296	Tmigd3	303.09	2.69
58	18101	Nmbr	431.60	2.69
59	27007	Klrk1	85.28	2.68
60	17295	Met	372.44	2.59
61	11541	Adora2b	289.59	2.57
62	15980	Ifngr2	3412.11	2.56
63	18391	Sigmar1	1056.27	2.54
64	56089	Ramp3	750.41	2.53
65	12145	Cxcr5	413.18	2.53
66	12425	Cckar	677.53	2.47
67	14745	Lpar1	916.11	2.42

	IDs	Symbols	combined_chisq	mean_l2fc
346	268902	Robo2	643.29	-1.19
347	15565	Htr6	210.21	-1.20
348	100042894	Vmn2r46	105.23	-1.20
349	18844	Plxna1	643.07	-1.22
350	26362	Axl	347.73	-1.23
351	14799	Gria1	1055.06	-1.23
352	319757	Smo	424.26	-1.27
353	14800	Gria2	590.33	-1.28
354	208154	Btla	91.19	-1.29
355	18187	Nrp2	846.08	-1.30
356	11447	Chrnd	147.66	-1.30
357	14744	Gpr65	138.30	-1.30
358	57781	Cd200r1	162.58	-1.30
359	14275	Folr1	155.33	-1.31
360	381810	Lpar5	105.51	-1.33
361	12267	C3ar1	221.81	-1.36
362	387285	Hcrtr2	382.60	-1.36
363	22253	Unc5c	515.79	-1.36
364	99296	Hrh3	743.98	-1.38
365	15563	Htr5a	983.04	-1.38
366	12518	Cd79a	132.36	-1.39
367	23832	Xcr1	288.92	-1.39
368	14608	Gpr83	597.29	-1.39
369	14812	Grin2b	924.55	-1.41
370	330814	Adgrl1	1157.82	-1.41
371	70839	P2ry12	388.21	-1.42
372	171463	Il17rd	303.38	-1.43
373	14767	Nmur1	97.66	-1.44
374	50931	Il27ra	127.79	-1.44
375	18442	P2ry2	108.82	-1.45
376	93897	Fzd10	215.28	-1.45
377	238377	Gpr68	525.41	-1.47
378	74229	Paqr8	770.56	-1.48
379	16161	Il12rb1	117.79	-1.50
380	58861	Cysltr1	139.09	-1.50
381	13836	Epha2	105.92	-1.52
382	104443	Npffr2	106.59	-1.53
383	18845	Plxna2	1093.34	-1.55
384	235611	Plxnb1	376.15	-1.55
385	65079	Rtn4r	422.77	-1.56
386	12801	Cnr1	744.97	-1.56
387	14365	Fzd3	778.13	-1.57
388	74775	Lmbr1l	317.78	-1.61
389	16197	Il7r	145.85	-1.62
390	211577	Mrgprf	201.19	-1.63
391	13845	Ephb3	355.71	-1.64
392	14255	Flt3	361.06	-1.64
393	20660	Sorl1	895.38	-1.64
394	625068	Vmn2r84	115.22	-1.65
395	383563	Gpr25	452.32	-1.65
396	27218	Slamf1	115.30	-1.65
397	15466	Hrh2	456.37	-1.66
398	16184	Il2ra	283.20	-1.70
399	107448	Unc5a	799.70	-1.72
400	14586	Gfra2	954.08	-1.73
401	18186	Nrp1	723.10	-1.74
402	14657	Glra4	150.85	-1.74
403	16975	Lrp8	755.77	-1.74
404	16971	Lrp1	780.25	-1.75
405	14816	Grm1	879.13	-1.75
406	20661	Sort1	1181.46	-1.77
407	13869	Erbb4	976.69	-1.77
408	93675	Clec2i	184.42	-1.77
409	11539	Adora1	747.62	-1.78
410	11608	Agtr1b	136.62	-1.79
411	20358	Sema6a	1212.40	-1.80
412	20606	Sstr2	585.98	-1.81

68	239283	Oxgr1	311.96	2.42
69	16847	Lepr	587.74	2.36
70	51801	Ramp1	769.32	2.34
71	15979	Ifngr1	629.96	2.32
72	70495	Atp6ap2	1284.42	2.32
73	20698	Sphk1	396.96	2.29
74	338346	Gpr21	313.60	2.29
75	14400	Gabrb1	1095.56	2.29
76	18211	Ntrk1	270.81	2.28
77	18160	Npr1	346.73	2.27
78	16194	Il6ra	501.40	2.25
79	14694	Rack1	1011.64	2.22
80	16190	Il4ra	452.82	2.20
81	20605	Sstr1	647.77	2.20
82	13610	S1pr3	462.62	2.18
83	14788	Gpr162	833.87	2.13
84	19228	Pth1r	468.62	2.12
85	22045	Trhr	595.47	2.11
86	14064	F2rl2	406.03	2.10
87	59049	Slc22a17	1742.82	2.10
88	230735	Epha10	522.22	2.07
89	12672	Chrm4	743.73	2.06
90	19713	Ret	1261.68	2.05
91	269831	Tspan12	853.03	1.99
92	237175	Adgrg2	418.42	1.98
93	11435	Chrna1	572.50	1.98
94	677296	Fcrl6	507.32	1.97
95	17087	Ly96	259.04	1.96
96	100129	Gpr153	1088.57	1.95
97	57249	Gabrq	1008.04	1.90
98	53978	Lpar2	330.10	1.89
99	238252	Gpr135	332.86	1.84
100	72333	Palld	436.78	1.81
101	12921	Crhr1	404.20	1.76
102	18613	Pecam1	548.76	1.75
103	64450	Gpr85	936.68	1.74
104	71904	Paqr7	832.07	1.70
105	18389	Oprl1	963.50	1.70
106	12802	Cnr2	306.02	1.66
107	213208	Il20rb	209.37	1.62
108	12909	Crcp	578.25	1.62
109	19885	Rorc	244.00	1.56
110	244238	Mrgpre	695.13	1.55
111	436090	Gpr62	541.28	1.54
112	57914	Crlf2	315.80	1.54
113	79201	Tnfrsf23	253.52	1.52
114	54598	Calcrl	412.76	1.52
115	19122	Prnp	1523.43	1.50
116	16155	Il10rb	268.39	1.49
117	245527	Eda2r	93.78	1.49
118	22355	Vipr2	310.37	1.46
119	237213	Glra2	688.46	1.45
120	21936	Tnfrsf18	218.53	1.44
121	16177	Il1r1	355.17	1.44
122	18703	Pigr	190.19	1.43
123	18430	Oxtr	447.83	1.42
124	19268	Ptprf	749.61	1.42
125	50905	Il17rb	186.32	1.41
126	229357	Gpr149	520.29	1.39
127	107515	Lgr4	593.57	1.36
128	16169	Il15ra	258.53	1.34
129	14748	Gpr3	256.46	1.34
130	12765	Cxcr2	446.86	1.30
131	230828	Il22ra1	419.09	1.29
132	14406	Gabrg2	515.31	1.28
133	14654	Glra1	781.94	1.26
134	18440	P2rx6	192.55	1.25
135	26563	Ror1	355.69	1.23
136	11610	Agtrap	350.34	1.22

413	11480	Acvr2a	1093.22	-1.83
414	210933	Adgrb3	1495.93	-1.84
415	12209	Brs3	219.89	-1.89
416	80978	Mrgprh	1050.15	-1.91
417	14368	Fzd6	464.83	-1.91
418	14810	Grin1	1176.12	-1.92
419	11551	Adra2a	683.93	-1.92
420	331493	Gm5127	210.87	-1.92
421	75552	Paqr9	1213.34	-1.92
422	27494	Amot	547.93	-1.94
423	15559	Htr2b	118.00	-1.95
424	21899	Tlr6	1049.16	-1.95
425	329693	Fcrl5	197.91	-1.96
426	246256	Fcgr4	178.14	-1.97
427	12669	Chrm1	1036.99	-1.98
428	11990	Atrn	1941.22	-1.99
429	242700	Ifnlr1	190.76	-2.00
430	16186	Il2rg	175.83	-2.01
431	242425	Gabbr2	1279.90	-2.01
432	14370	Fzd8	388.75	-2.02
433	231876	Lmtk2	1752.28	-2.02
434	19216	Ptger1	604.85	-2.04
435	14408	Gabrr1	161.67	-2.04
436	13840	Epha6	1071.36	-2.05
437	328833	Treml2	179.84	-2.05
438	64297	Gprc5b	778.10	-2.06
439	21337	Tacr2	177.07	-2.06
440	17480	Mpl	169.20	-2.08
441	217305	Cd300ld	180.34	-2.10
442	319387	Adgrl3	1898.67	-2.11
443	78560	Adgra2	530.23	-2.12
444	18779	Pla2r1	318.50	-2.15
445	13176	Dcc	1619.79	-2.15
446	14276	Folr2	1496.90	-2.15
447	140497	Cd300c2	182.74	-2.16
448	16398	Itga2	167.11	-2.16
449	209590	Il23r	164.88	-2.17
450	14738	Gpr12	1114.60	-2.20
451	16001	Igf1r	1415.15	-2.22
452	16195	Il6st	1609.70	-2.23
453	18441	P2ry1	936.14	-2.24
454	12804	Cntfr	974.81	-2.24
455	13838	Epha4	1208.32	-2.25
456	14402	Gabrb3	2296.14	-2.25
457	21812	Tgfbr1	1124.60	-2.27
458	56644	Clec7a	230.12	-2.27
459	19207	Ptch2	248.17	-2.27
460	108068	Grm2	537.53	-2.29
461	12931	Crlf1	198.86	-2.30
462	21941	Tnfrsf8	222.00	-2.30
463	14102	Fas	157.16	-2.30
464	16974	Lrp6	1806.20	-2.31
465	11552	Adra2b	497.11	-2.32
466	12057	Opn1sw	213.55	-2.33
467	14129	Fcgr1	281.44	-2.34
468	243743	Plxna4	1784.60	-2.37
469	15557	Htr1f	376.96	-2.37
470	110835	Chrna5	682.45	-2.38
471	18053	Ngfr	525.02	-2.40
472	16768	Lag3	274.32	-2.43
473	16337	Insr	1624.01	-2.43
474	24088	Tlr2	218.57	-2.46
475	233079	Ffar2	264.50	-2.47
476	18667	Pgr	2161.86	-2.47
477	14254	Flt1	604.06	-2.50
478	216749	Nmur2	196.48	-2.51
479	170639	Olfr78	1186.11	-2.52
480	13841	Epha7	1654.18	-2.52
481	83433	Trem2	258.58	-2.54

137	239845	Gpr156	173.30	1.22
138	108069	Grm3	577.69	1.21
139	14763	Gpr37	338.20	1.14
140	18217	Ntsr2	471.02	1.12
141	16419	Itgb5	276.38	1.08
142	18216	Ntsr1	580.56	1.08
143	12215	Bsg	586.51	1.07
144	107817	Jmjd6	258.75	1.05
145	72674	Adipor1	723.40	1.05
146	14588	Gfra4	334.87	1.04
147	52389	Adgra1	746.56	1.04
148	14760	Gpr19	270.63	1.04
149	66205	Cd302	205.38	1.02
150	11553	Adra2c	387.31	1.01
151	13618	Ednrb	437.20	1.00
152	70355	Gprc5c	322.55	0.99
153	17199	Mc1r	361.65	0.98
154	110326	Tas1r1	185.15	0.98
155	16149	Cd74	342.60	0.95
156	12986	Csf3r	270.67	0.92
157	21940	Cd27	139.22	0.90
158	15975	Ifnar1	590.45	0.87
159	19204	Ptafr	218.56	0.86
160	68957	Paqr6	297.39	0.86
161	12426	Cckbr	482.72	0.85
162	58235	Nectin1	303.93	0.83
163	56632	Sphk2	323.97	0.83
164	207911	Mchr1	311.70	0.82
165	16412	Itgb1	463.70	0.81
166	20778	Scarb1	284.15	0.80
167	15560	Htr2c	657.86	0.80
168	68465	Adipor2	279.20	0.79
169	54393	Gabbr1	731.64	0.77
170	15976	Ifnar2	248.73	0.75
171	17005	Ltk	140.35	0.74
172	217369	Uts2r	663.24	0.74
173	16004	Igf2r	300.63	0.72
174	114229	Kiss1r	128.94	0.72
175	11444	Chrnb2	260.45	0.71
176	625131	Vmn2r87	101.94	0.69
177	16162	Il12rb2	125.30	0.69
178	13842	Epha8	350.82	0.66
179	21336	Tacr1	436.29	0.66
180	16423	Cd47	392.87	0.66
181	14182	Fgfr1	557.03	0.65
182	19219	Ptger4	170.17	0.63
183	235472	Prtg	185.61	0.57
184	21813	Tgfbr2	233.03	0.57
185	78134	Lpar4	213.05	0.56
186	93690	Gpr45	217.31	0.56
187	16180	Il1rap	198.39	0.54
188	245526	Pgr15l	474.65	0.54
189	19062	Inpp5k	185.79	0.50
190	213439	Gpr174	190.73	0.49
191	70693	Adgra3	191.89	0.49
192	56873	Lmbr1	197.82	0.49
193	245424	Gpr101	351.80	0.47
194	240888	Gpr161	211.23	0.46
195	12305	Ddr1	194.14	0.45
196	13848	Ephb6	239.55	0.44
197	13844	Ephb2	249.25	0.42
198	259104	Olfr613	209.01	0.38
199	52685	Cd300lg	176.08	0.37
200	14405	Gabrg1	433.74	0.37
201	140571	Plxnb3	245.25	0.37
202	243764	Chrm2	272.79	0.33
203	12505	Cd44	251.58	0.33
204	11554	Adrb1	417.12	0.32
205	240239	Gpr151	172.39	0.32
482	252837	Ackr4	212.09	-2.55
483	244058	Rgma	1315.97	-2.56
484	13490	Drd3	222.67	-2.57
485	243270	Hcar1	276.03	-2.58
486	12168	Bmpr2	3067.50	-2.60
487	12773	Ccr4	336.53	-2.63
488	23920	Insrr	353.75	-2.64
489	245537	Nlgn3	2670.25	-2.65
490	241263	Gpr158	2737.04	-2.65
491	12671	Chrm3	719.32	-2.66
492	57014	Htr3b	293.77	-2.68
493	107831	Adgrb1	1898.16	-2.69
494	237847	Rtn4rl1	1302.96	-2.70
495	53623	Gria3	2354.07	-2.72
496	14183	Fgfr2	1154.40	-2.81
497	21667	Tdgf1	252.33	-2.83
498	14813	Grin2c	673.86	-2.85
499	14289	Fpr2	1087.92	-2.88
500	16880	Lifr	1486.63	-2.89
501	16867	Lhcgr	281.54	-2.96
502	65969	Cubn	346.45	-2.98
503	21942	Tnfrsf9	278.37	-2.99
504	71790	Anxa9	239.60	-3.04
505	15170	Ptpn6	303.35	-3.05
506	21390	Tbxa2r	578.48	-3.06
507	257632	Nod2	267.91	-3.07
508	14428	Galr2	255.65	-3.09
509	17076	Ly75	314.99	-3.09
510	269902	Vmn2r57	279.94	-3.10
511	12772	Ccr2	342.74	-3.10
512	19882	Mst1r	290.98	-3.11
513	12984	Csf2rb2	440.20	-3.14
514	623734	Vmn2r85	279.71	-3.17
515	19217	Ptger2	416.76	-3.17
516	14917	Gucy2c	283.99	-3.19
517	11481	Acvr2b	1788.23	-3.20
518	69538	Antxr1	1202.91	-3.21
519	170483	Grin3b	292.95	-3.24
520	233571	P2ry6	322.56	-3.25
521	100042499	Vmn2r55	682.63	-3.26
522	18007	Neo1	3593.98	-3.27
523	20737	Spn	351.69	-3.28
524	140741	Gpr6	469.01	-3.29
525	18846	Plxna3	1134.00	-3.30
526	74144	Robo4	357.50	-3.36
527	545902	Ptprh	299.98	-3.38
528	241070	Gpr1	311.56	-3.38
529	13609	S1pr1	1417.96	-3.39
530	83771	Tas1r3	367.01	-3.39
531	13837	Epha3	714.08	-3.39
532	19206	Ptch1	2875.26	-3.39
533	16408	Itgal	327.84	-3.42
534	21939	Cd40	1481.85	-3.42
535	14725	Lrp2	1246.42	-3.43
536	14539	Opn1mw	337.03	-3.48
537	71862	Gpr160	1227.53	-3.50
538	435653	Fcrlb	396.34	-3.50
539	53791	Tlr5	1610.36	-3.55
540	13835	Epha1	349.18	-3.55
541	12374	Casr	355.50	-3.56
542	23890	Gpr34	396.01	-3.57
543	14765	Gpr50	422.49	-3.60
544	14131	Fcgr3	347.06	-3.62
545	11482	Acvrl1	411.65	-3.64
546	12517	Cd72	1240.50	-3.65
547	229214	Qrfpr	368.73	-3.67
548	73707	Gucy2g	438.79	-3.69
549	14362	Fzd1	1450.11	-3.72
550	14811	Grin2a	2868.01	-3.80

206	14766	Adgrg1	486.82	0.31
207	381413	Gpr176	220.22	0.30
208	18166	Npy1r	305.14	0.28
209	14802	Gria4	489.53	0.28
210	210801	Unc5d	409.69	0.27
211	18387	Oprk1	314.47	0.26
212	73010	Gpr22	321.80	0.25
213	54140	Avpr1a	282.45	0.24
214	18129	Notch2	233.76	0.24
215	16590	Kit	586.40	0.22
216	230103	Npr2	138.61	0.20
217	11682	Alk	226.05	0.20
218	23802	Amfr	244.81	0.19
219	18213	Ntrk3	257.16	0.19
220	21846	Tie1	326.96	0.18
221	14403	Gabrd	269.65	0.17
222	15561	Htr3a	250.29	0.15
223	107934	Celsr3	254.36	0.10
224	270190	Ephb1	278.26	0.07
225	19218	Ptger3	375.14	0.07
226	14658	Glrb	188.50	0.06
227	15566	Htr7	417.47	0.03
228	27403	Abca7	95.16	0.01
229	11517	Adcyap1r1	447.47	0.01
230	259097	Olfr558	41.11	-0.01
231	13492	Drd5	204.09	-0.04
232	14394	Gabra1	275.39	-0.05
233	54712	Plxnc1	474.38	-0.05
234	238725	Gpr150	121.11	-0.07
235	14823	Grm8	224.13	-0.07
236	98682	Mfsd6	363.72	-0.09
237	13839	Epha5	436.80	-0.09
238	64378	Gpr88	929.80	-0.09
239	216856	Nlgn2	143.33	-0.09
240	18438	P2rx4	189.20	-0.12
241	19274	Ptprm	537.58	-0.13
242	245650	Gucy2f	134.66	-0.18
243	108071	Grm5	594.95	-0.19
244	110542	Amhr2	110.17	-0.20
245	217303	Cd300a	265.12	-0.22
246	19281	Ptprt	577.20	-0.22
247	13617	Ednra	239.33	-0.23
248	12767	Cxcr4	215.80	-0.23
249	20187	Ryk	190.06	-0.23
250	14805	Grik1	243.71	-0.23
251	15985	Cd79b	78.89	-0.24
252	14806	Grik2	360.26	-0.28
253	14803	Grid1	394.55	-0.30
254	54608	Abhd2	292.98	-0.31
255	208898	Unc13c	636.77	-0.32
256	170744	Tlr8	64.59	-0.33
257	18189	Nrxn1	201.37	-0.34
258	13603	Opn3	335.34	-0.37
259	18198	Musk	308.98	-0.38
260	12483	Cd22	228.95	-0.40
261	14761	Gpr27	120.63	-0.44
262	14062	F2r	398.28	-0.44
263	268934	Grm4	373.33	-0.45
264	29820	Tnfrsf19	288.91	-0.45
265	212541	Rho	77.20	-0.47
266	14066	F3	240.23	-0.49
267	11438	Chrna4	565.66	-0.52
268	14397	Gabra4	506.45	-0.52
269	228139	P2rx3	134.04	-0.53
270	54672	Adgrg3	66.22	-0.54
271	94071	Clec2h	215.19	-0.55
272	18190	Nrxn2	244.10	-0.57
273	13489	Drd2	611.38	-0.57
274	65086	Lpar3	82.08	-0.58

551	14404	Gabre	545.05	-3.87
552	110789	Adgrv1	1348.21	-3.87
553	226304	Npbwr1	570.55	-3.91
554	16414	Itgb2	364.77	-3.91
555	21934	Tnfrsf11a	1253.93	-3.94
556	11440	Chrna6	663.27	-3.94
557	26564	Ror2	1859.93	-3.99
558	63873	Trpv4	479.66	-3.99
559	227326	Gpr55	429.92	-4.00
560	93896	Glp2r	451.53	-4.00
561	22095	Tshr	446.63	-4.04
562	140795	P2ry14	388.50	-4.04
563	12519	Cd80	847.77	-4.06
564	101613	Nlrp6	423.72	-4.08
565	170743	Tlr7	432.31	-4.08
566	58182	Prokr1	1623.03	-4.11
567	71914	Antxr2	415.28	-4.13
568	208795	Tmem63a	1325.40	-4.15
569	80891	Fcrls	520.34	-4.16
570	218624	Il31ra	590.82	-4.18
571	93694	Clec2d	489.57	-4.19
572	56544	Vmn2r1	1444.29	-4.20
573	14814	Grin2d	1447.50	-4.22
574	110902	Chrna2	696.96	-4.22
575	18763	Pkd1	2434.52	-4.25
576	54199	Ccrl2	1580.60	-4.25
577	18128	Notch1	1059.64	-4.26
578	21824	Thbd	764.78	-4.27
579	19220	Ptgfr	479.45	-4.28
580	14063	F2rl1	586.73	-4.28
581	54215	Cd160	508.12	-4.30
582	16154	Il10ra	484.02	-4.36
583	21898	Tlr4	509.53	-4.46
584	14739	S1pr2	490.41	-4.47
585	12769	Ccr9	547.55	-4.49
586	13867	Erbb3	519.52	-4.51
587	329064	Pkd2l1	1716.67	-4.53
588	19214	Ptgdr	645.82	-4.53
589	142980	Tlr3	1057.28	-4.54
590	12902	Cr2	498.87	-4.56
591	232790	Oscar	531.90	-4.57
592	71461	Ptk7	636.42	-4.60
593	94226	S1pr5	1276.74	-4.63
594	107449	Unc5b	1773.68	-4.67
595	17533	Mrc1	560.54	-4.68
596	21937	Tnfrsf1a	661.83	-4.75
597	114332	Lyve1	1464.76	-4.78
598	57265	Fzd2	631.16	-4.78
599	14747	Cmklr1	637.96	-4.83
600	14807	Grik3	4329.79	-4.92
601	21687	Tek	629.26	-4.92
602	22354	Vipr1	778.38	-5.06
603	13649	Egfr	1389.11	-5.08
604	12166	Bmpr1a	6251.56	-5.10
605	17171	Mas1	722.59	-5.10
606	11540	Adora2a	727.36	-5.11
607	14829	Grpr	774.40	-5.14
608	59289	Ackr2	1930.75	-5.15
609	329252	Lgr6	680.76	-5.16
610	20928	Abcc9	899.39	-5.17
611	18214	Ddr2	791.23	-5.18
612	14127	Fcer1g	686.59	-5.25
613	319480	Itga11	678.69	-5.29
614	74039	Nfam1	788.95	-5.30
615	108043	Chrnb3	1086.49	-5.34
616	116701	Fgfrl1	783.15	-5.36
617	14257	Flt4	782.08	-5.37
618	14652	Glp1r	746.89	-5.37
619	16174	Il18rap	993.81	-5.43

275	68799	Rgmb	334.59	-0.58
276	14407	Gabrg3	501.48	-0.58
277	217733	Tmem63c	250.19	-0.59
278	13488	Drd1	556.82	-0.60
279	14600	Ghr	321.40	-0.60
280	14401	Gabrb2	294.53	-0.60
281	11609	Agtr2	440.39	-0.61
282	224807	Tmem63b	229.68	-0.62
283	13051	Cx3cr1	286.35	-0.64
284	70771	Gpr173	209.12	-0.64
285	22359	Vldlr	402.91	-0.64
286	70086	Cysltr2	72.89	-0.65
287	100043123	Cd300ld4	105.00	-0.66
288	18595	Pdgfra	234.97	-0.66
289	237716	Gpr75	320.29	-0.67
290	269053	Gpr152	77.61	-0.67
291	16192	Il5ra	192.55	-0.68
292	108073	Grm7	364.80	-0.69
293	140570	Plxnb2	280.88	-0.69
294	14809	Grik5	301.73	-0.69
295	13982	Esr1	412.02	-0.70
296	17201	Mc3r	216.65	-0.70
297	19116	Prlr	400.07	-0.70
298	246746	Cd300lf	184.20	-0.72
299	12273	C5ar1	159.32	-0.73
300	18132	Notch4	253.44	-0.74
301	14366	Fzd4	224.29	-0.75
302	110886	Gabra5	847.53	-0.75
303	14369	Fzd7	264.76	-0.76
304	171469	Gpr37l1	531.98	-0.76
305	192167	Nlgn1	499.17	-0.77
306	242443	Grin3a	785.68	-0.80
307	16973	Lrp5	270.30	-0.80
308	269275	Acvr1c	296.79	-0.80
309	18793	Plaur	145.16	-0.81
310	16182	Il18r1	162.56	-0.83
311	20607	Sstr3	283.03	-0.84
312	57385	P2ry4	170.73	-0.84
313	16188	Il3ra	81.04	-0.87
314	22174	Tyro3	391.99	-0.87
315	67784	Plxnd1	309.25	-0.88
316	11536	Gpr182	217.27	-0.89
317	110637	Grik4	280.10	-0.89
318	224792	Adgrf5	438.94	-0.90
319	19876	Robo1	543.35	-0.90
320	11477	Acvr1	196.31	-0.90
321	12506	Cd48	79.50	-0.91
322	11549	Adra1a	510.26	-0.91
323	319430	C5ar2	253.65	-0.92
324	21897	Tlr1	91.40	-0.92
325	14395	Gabra2	498.23	-0.94
326	269604	Gpr157	259.87	-0.96
327	230775	Adgrb2	386.76	-0.97
328	99633	Adgrl2	691.31	-0.99
329	76229	Vmn2r29	211.92	-0.99
330	74603	Cd200r3	213.90	-1.03
331	277328	Trpa1	76.94	-1.05
332	18212	Ntrk2	1033.36	-1.05
333	12500	Cd3d	367.15	-1.09
334	12982	Csf2ra	264.48	-1.09
335	110168	Gpr18	438.14	-1.09
336	17082	Il1rl1	193.67	-1.12
337	14396	Gabra3	919.09	-1.12
338	16728	L1cam	605.67	-1.13
339	21814	Tgfbr3	428.62	-1.14
340	15551	Htr1b	409.55	-1.16
341	171095	Il17rc	274.23	-1.16
342	53883	Celsr2	755.71	-1.17
343	16420	Itgb6	195.82	-1.17

620	620246	Gpr52	910.48	-5.48
621	12614	Celsr1	731.39	-5.52
622	18414	Osmr	727.24	-5.54
623	22249	Unc13b	2804.87	-5.57
624	215798	Adgrg6	832.61	-5.59
625	170732	Trhr2	2893.00	-5.62
626	11443	Chrnb1	980.22	-5.66
627	107607	Nod1	901.01	-5.67
628	15562	Htr4	997.48	-5.70
629	234542	Rtbdn	905.07	-5.75
630	16172	Il17ra	1272.28	-5.78
631	18390	Oprm1	2345.36	-5.79
632	11479	Acvr1b	5371.42	-5.81
633	110834	Chrna3	1173.25	-5.86
634	121021	Cspg4	917.69	-5.94
635	67168	Lpar6	920.42	-5.99
636	12978	Csf1r	1066.77	-6.03
637	26364	Adgre5	964.02	-6.05
638	243277	Adgrd1	1000.19	-6.18
639	14184	Fgfr3	1353.24	-6.25
640	108015	Chrnb4	1252.19	-6.33
641	13857	Epor	981.83	-6.36
642	15446	Hpgd	1090.70	-6.37
643	12504	Cd4	1000.59	-6.37
644	18439	P2rx7	1047.02	-6.37
645	20608	Sstr4	1040.69	-6.39
646	269295	Rtn4rl2	2787.12	-6.41
647	21338	Tacr3	1399.17	-6.47
648	18596	Pdgfrb	1101.84	-6.50
649	12778	Ackr3	1124.61	-6.54
650	14409	Gabrr2	1501.36	-6.56
651	14367	Fzd5	1157.02	-6.63
652	14919	Gucy2e	1182.45	-6.67
653	18131	Notch3	1202.77	-6.69
654	18386	Oprd1	1279.77	-6.71
655	16164	Il13ra1	1244.90	-6.73
656	14160	Lgr5	1360.95	-6.84
657	53614	Reck	1485.14	-7.27
658	574402	Gpr17	1471.36	-7.29
659	12167	Bmpr1b	1403.97	-7.40
660	17289	Mertk	1290.63	-7.40
661	246313	Prokr2	1475.26	-7.50
662	16421	Itgb7	2043.40	-7.77
663	319239	Npsr1	2428.80	-7.80
664	11441	Chrna7	1926.06	-7.82
665	233919	Gpr26	1923.78	-7.93
666	15558	Htr2a	2080.60	-8.12
667	81006	Gpr63	1974.68	-8.30
668	80910	Gpr84	1981.69	-8.34
669	170757	Adgrl4	1908.94	-8.52
670	81897	Tlr9	2276.08	-8.60
671	14804	Grid2	2186.10	-8.75
672	11303	Abca1	2283.21	-9.01
673	229323	Gpr171	1999.72	-9.05
674	279572	Tlr13	2094.65	-9.85
675	76854	Gper1	2133.04	-10.02
676	12774	Ccr5	2482.96	-10.03
677	73182	Pear1	2099.25	-10.05
678	381489	Rxfp1	3042.30	-10.13
679	74191	P2ry13	2332.05	-10.15
680	13866	Erbb2	2767.05	-10.28
681	244646	Pkd1l3	2289.73	-11.80
682	13846	Ephb4	3158.97	-12.46
683	213788	Chrm5	4250.33	-12.65
684	16542	Kdr	4151.22	-13.10
685	73340	Nptxr	4062.25	-13.13
686	209776	Gpr139	4000.87	-13.24
687	13805	Eng	3848.33	-13.72
688	20897	Stra6	3032.09	-14.60

**344**	23796	Aplnr	113.92	-1.18
**345**	20354	Sema4d	504.25	-1.19
**689**	12503	Cd247	2710.40	-15.71
**690**	11550	Adra1d	4003.26	-15.72
**691**	320910	Itgb8	8453.46	-17.43

Supplementary Table 4: CEREP eurofins selectivity assay to CVN45502

Assay Name	Species	n	Concentration tested (µM) (CVN45502)	% Inhibition
5-Lipoxygenase	hum	2	10	14
ATPase, Ca2+, Skeletal muscle	pig	2	10	7
ATPase, Na+/K+, Heart	pig	2	10	-12
Carbonic Anhydrase II	hum	2	10	-4
Cholinesterase, Acetyl, ACES	hum	2	10	-8
Cyclooxygenase COX-1	hum	2	10	5
Cyclooxygenase COX-2	hum	2	10	-13
HMG-CoA Reductase	hum	2	10	-4
Monoamine Oxidase MAO-A	hum	2	10	11
Monoamine Oxidase MAO-B	hum	2	10	8
Nitric Oxide Synthase, neuronal (nNOS)	rat	2	10	22
Nitric Oxide Synthase, inducible (iNOS)	mouse	2	10	5
Peptidase, Factor Xa	hum	2	10	-2
Peptidase, Matrix metalloprotease-1 (MMP-1)	hum	2	10	18
Peptidase, Matrix metalloprotease-7 (MMP-7)	hum	2	10	6
Peptidase, Matrix metalloprotease-13 (MMP-13)	hum	2	10	-4
Peptidase, Matrix metalloprotease, neutral endopeptidase	hum	2	10	4
Peptidase, Renin	hum	2	10	10
Peptidase, Tumor necrosis factorα Converting enzyme (TACE)	hum	2	10	2
Phosphodiesterase PDE10A2	hum	2	10	35
Phosphodiesterase PDE3	hum	2	10	9
Phosphodiesterase PDE4	hum	2	10	36
Phosphodiesterase PDE5	hum	2	10	29
Phosphodiesterase PDE6	bov	2	10	3
Phospholipase PLA2-I	pig	2	10	10
Protein Serine/Threonine Kinase, ATK1 (PRKBA)	hum	2	10	-7
Protein Serine/Threonine Kinase, Calcium calmodulin II	rat	2	10	16
Protein Serine/Threonine Kinase, CDC2/CCNB1 (CDK1/cyclin	hum	2	10	14
Protein Serine/Threonine Kinase, CDK2/CCNE (cdk3/cyclinE)	hum	2	10	6
Protein Serine/Threonine Kinase, GSK3B	hum	2	10	8
Protein Serine/Threonine Kinase, IKK-1	hum	2	10	-14
Protein Serine/Threonine Kinase, MAP2K1 (MEK1)	hum	2	10	-4
Protein Serine/Threonine Kinase, MAPK1 (ERK2)	hum	2	10	5
Protein Serine/Threonine Kinase, MAPK14 (p38a)	hum	2	10	-1
Protein Serine/Threonine Kinase, MAPK3 (ERK1)	hum	2	10	8
Protein Serine/Threonine Kinase, MAPK8 (JNK1)	hum	2	10	6
Protein Serine/Threonine Kinase, PKC, non-selective	rat	2	10	-5
Protein Serine/Threonine Kinase, PRKACA (PKA)	hum	2	10	-5
Protein Serine/Threonine Phosphatase, PPP3CA (Calccineuri	hum	2	10	12
Protein Tyrosine Kinase, EGF receptor	hum	2	10	-22
Protein Tyrosine Kinase, ERBB2 (HER2)	hum	2	10	0
Protein Tyrosine Kinase, FES	hum	2	10	-13
Protein Tyrosine Kinase, Insulin receptor	hum	2	10	-14
Protein Tyrosine Kinase, LYN B	hum	2	10	6

Protein Tyrosine Kinase, SRC	hum	2	10	-7
Protein Tyrosine Kinase, ZA70 (ZAP-70)	hum	2	10	-1
Steroid 5α-Reductase	rat	2	10	6
Xanthine Oxidase	bov	2	10	16
Adenosine A1	hum	2	10	7
Adenosine A2A	hum	2	10	2
AdenosineA2B	hum	2	10	-7
Adenosine A3	hum	2	10	-2
Adrenergic α1, non-selective	rat	2	10	0
Adrenergic α2, non-selective	rat	2	10	0
Adrenergic β1	hum	2	10	-14
Adrenergic β2	hum	2	10	0
Adrenergic β3	hum	2	10	0
Anaphylatoxin C5a	hum	2	10	6
Androgen (Testosterone)	hum	2	10	-5
Angiotensin AT1	hum	2	10	-1
Angiotensin AT2	hum	2	10	5
APJ	hum	2	10	-4
Bradykinin B1	hum	2	10	4
Bradykinin B2	hum	2	10	-18
Calcium Channel L-type, Benzothiazepine	rat	2	10	22
Calcium Channel L-type, Dihydropyridine	rat	2	10	4
Calcium Channel L-type, Phenylalkylamine	rat	2	10	16
Calcium Channel N-type	rat	2	10	-1
Cannabinoid CB1	hum	2	10	8
Chemokine CXCR1/2 Il8, non-selective)	hum	2	10	2
Cholecystokinin CCK1	hum	2	10	-18
Cholecystokinin CCK2	hum	2	10	5
Dopamine D1	hum	2	10	2
Dopamine D2L	hum	2	10	7
Dopamine D3	hum	2	10	-2
Dopamine D4.2	hum	2	10	-9
Estrogen Erα	hum	2	10	2
Estrogen ERβ	hum	2	10	4
GABAA, Chloride Channel, TBOB	rat	2	10	21
GABAA, Flunitrazepam, central	rat	2	10	-10
GABAA, Muscimol, central	rat	2	10	-4
GABAB, Non-Selective	rat	2	10	9
GABAB1A	hum	2	10	-8
GABAB1B	hum	2	10	-1
Glucocorticoid	hum	2	10	13
Glutamate, AMPA	rat	2	10	0
Glutamate, Kainate	rat	2	10	1
Glutamate, NMDA, Agonism	rat	2	10	-5
Glutamate, NMDA, Glycine	rat	2	10	-7
Glutamate, NMDA, Phencyclidine	rat	2	10	-1
Glycine, Strychnine-Sensitive	rat	2	10	5
Growth Hormone Secretogogue (GHS, Ghrelin)	hum	2	10	-5

Histamine H1	hum	2	10	2
Histamine H2	hum	2	10	-14
Histamine H3	hum	2	10	-4
Imidazoline I2, central	rat	2	10	-9
Insulin	rat	2	10	2
Muscarinic M1	hum	2	10	-1
Muscarinic M2	hum	2	10	-7
Muscarinic M3	hum	2	10	-1
Muscarinic M4	hum	2	10	10
Nicotinic Acetylcholine receptor, non-specific	hum	2	10	-6
Opiate δ1 (OP1, DOP)	hum	2	10	-4
Opiate κ(OP2, KOP)	hum	2	10	3
Opiate μ(OP3, MOP)	hum	2	10	-3
Phorbol Ester	mouse	2	10	-4
Potassium Channel (KATP)	ham	2	10	9
Potassium Channel (SKCA)	rat	2	10	-7
Progesterone PR-B	hum	2	10	21
Serotonin (5-Hydroxytryptamine) 5-HT1, non-selective	rat	2	10	12
Serotonin (5-Hydroxytryptamine) 5-HT2, non-selective	rat	2	10	0
Serotonin (5-Hydroxytryptamine) 5-HT2B	hum	2	10	4
Serotonin (5-Hydroxytryptamine) 5-HT3	hum	2	10	-9
Serotonin (5-Hydroxytryptamine) 5-HT4	gp	2	10	-4
Sigma, Non-selective	gp	2	10	20
Sodium Channel, site 2	rat	2	10	4
Tachykinin NK1	hum	2	10	12
Tachykinin NK2	hum	2	10	-8
Tachykinin NK3	hum	2	10	27
Transporter, Dopamine (DAT)	hum	2	10	4
Transporter, GABA	rat	2	10	3
Transporter, Norepinephrine (NET)	hum	2	10	-6
Transporter, Serotonin (SERT)	hum	2	10	7
Vasopressin V1a	hum	2	10	-14
Vasopressin V1b	hum	2	10	-8
Vasopressin V2	hum	2	10	-4
Key				
bov=Bovine				
gp=Guinea pig				
ham=Hamster				
hum=Human				

Supplementary table 5_Source table

REAGENT OR RESOURCE	SOURCE	IDENTIFIER
**Antibodies and ISH probes**		
Slc32a1-C3 mouse	Acdbio	Catalog # 319191
Slc17a6-C3 mouse	Acdbio	Catalog # 319171
Slc17a8-C2-human	Acdbio	Catalog # 487431
Hcrtr1-C1-human	Acdbio	Catalog # 312588
Slc17a8-C3 mouse	Acdbio	Catalog # 431261
CalcR-C2 mouse	Acdbio	Catalog # 317518
Hcrtr1-C2 mouse	Acdbio	Catalog # 466638
Gpcr4-C2 mouse	Acdbio	Catalog # 427948
Anti-Hcrtr1-(human)- PA5-33838 (1:100)	Thermofisher	Catalog # PA5-33838; RRID: AB_2551207
Alexa Fluor 488 AffiPure Donkey Anti-Mouse IgG (H+L) (1:1000)	Thermofisher	Catalog # A-21202; RRID: AB_141607
Alexa Fluor 647 AffiPure Donkey Anti-Rabbitt IgG (H+L) (1:1000)	Thermofisher	Catalog # A-31573; RRID: AB_2536183
Alexa Fluor 488 AffiPure Donkey Anti-Rabbitt IgG (H+L) (1:1000)	Thermofisher	Catalog # A-21206; RRID: AB_2535792
Chicken-anti-GFP (1:2000)	AVES-LABS	Catalog # GFP-1020; RRID: AB_2307313
Anti-GFAP rabbit polyclonal antibody (1:5000)	Abcam	Catalog # AB7260
Anti-RFP (RABBIT) (1:2000)	ROCKLAND	Catalog # 600-401-379
**Biological samples**		
Human brain tissue	Tissues for Research (UK) and General Section of the Douglas-Bell Canada Brain Bank (Montreal)	
Mouse tissue samples	Tissues obtained in our lab (This study)	IACUC protocol 18066-H
**Reagents**		
Clozapine-N-Oxide	Sigma-Aldrich	Cat # 34233-69-7
Salmon Calcitonin	Sigma-Aldrich	Cat # SCP0121
SB-334867 Orexin Receptor Antagonist	Tocris	Cat # 1960
CVN45502 Orexin 1 receptor antagonist	MTA agreement with Cerevance	N/A
Orexin A	Sigma-Aldrich	Cat # O6012
Insulin	Sigma-Aldrich	Cat#I0516
Glucose	Sigma-Aldrich	Cat # SKU49163
ACSF	Tocris	Cat # 35-252
Choloroform	Bio-Lab	Cat# 3082301
Phenol:Chloroform:Isoamyl Alcohol (25:24:1)	Sigma-Aldrich	Cat#P2069
Methanol	Bio-Lab	Cat#136806
TRIzol™	Invitrogen	Cat# 15596026
Multiplex kit RNAscope	Acdbio	Cat # 323136
Agilent Envision Plus high Ph solution	Agilent	Cat # K800221-5
Agilent Envision plus secondary antibodies detection kit	Agilent	Cat # K400111-2
Plastics one cannulae	Plastics One	Custome size
Thor Optogenetic fibers	THOR Labs	200um Core, 0.39 NA fiber
Angiotensin 2	Sigma-Aldrich	Cat # 4474-91-3
IPTT-300 transponders	Fisher Scientific	Cat # NC9207011
Saline solution	Sigma-Aldrich	Cat # 7647-14-5
VECTASHIELD Antifade Mounting Medium with DAPI	Vector labs	Cat#H-1200
Fluoroshield with DAPI	Sigma-Aldrich	Cat#F6057
Dibenzyl ether	Sigma-Aldrich	Cas-103-50-4
Dicloromethane	Sigma-Aldrich	Cat # SKU270997
Heparin	Sigma-Aldrich	Cat # 1304016
Triton-X 100	Sigma-Aldrich	Cat # SKU93443
Bovine serum albumin	Sigma-Aldrich	Cat # SKU 10711454001
Protease Inhibitor Cocktail	Sigma-Aldrich	Cat#P8340
Tween-20	Sigma-Aldrich	Cat#P1379
Paraformaldehyde, 16%	Electron Microscopy Sciences	Cat#30525-89-4
Isoflurane, USP	Sigma-Aldrich	Cat # 1349003
Donkey serum	Sigma-Aldrich	Cat # D9663
**Experimental Models: Cell Lines**		
FFPE-CHEM1 cell lines	AMSBIO	N/A
HEK-cell lines	Invitrogen	Cat # R70007
**Experimental Models: Organisms/Strains**		
Mouse: C57BL/6J	Jackson Laboratory	Stock 000664
Mouse: Vglut3-IRES-Cre	Gift from Bradford Lowell	N/A
Mouse: Vglut-IRES-Cre	Jackson Laboratory	Stock 016963
Mouse: Ob/Ob	Jackson Laboratory	Stock 000662
**Viral vectors**		
AAV9-CAG-FLEX-GFP	Addgene	Cat # 51502
AAV5-DIO-Synaptophysin-venus-GFP	Addgene	Cat # 51502
AAV5-Ef1A-DIO-GTB	Addgene	Cat # 51503
AAV5-Ef1A-DIO-TVA-mCherry	Addgene	Cat # 37084
EnvA-SAD-Rb-deltaG-GFP	Salk Institute	Cat # 32635
AAV5-EF1a-DIO-hM3Dq-mCherry	Addgene	Cat # 44631
AAV5-EF1a-DIO-mCherry	Addgene	Cat # 44632
AAV5-EF1a-DIO-hChR2(H134R)-EYFP	Addgene	Cat # 50462
AAV5-EF1a-DIO-EYFP	Addgene	Cat # 20298
AAV5-EF1a-DIO-hM4Di-mCherry	Addgene	Cat # 27056
**Master mix/Reagents/Enzymes**		
EconoTaq® Green Master Mix	Lucigen	Cat# 30033-2
SYBR® Green MasterMix®	Applied Biosystems	Cat # 4304437
MultiScribe™ Reverse Transcriptase	Invitrogen	Cat#4311235
Proteinase K	New England Biolabs	Cat#P8107S
**Animal diet**		
Maintenance diet for mice	Research Diets	DS12492
High fat diet	Research Diets	DS12451
Peanut butter	Jif Creamy	N/A
**Experimental instruments**		
Lavision Light Sheet Microscope	Miltenyibiotec	Ultramicroscope II
Confocal Microscope	Zeiss	Zeiss LSM780
Quantstudio	Applied biosystems	Quantstudio 3 96 well qPCR
Magnetic ressonance body composition	EchoMRI	EchoMRI
Phenomaster TSE system	TSE Systems	Phenomaster Next Generation
**Optogenetic lasers**	Optoengine	473nm Optoengine laser
**Ethovision camera and arenas**	Noldus	EthoVisionXT
**Nanozoomer**	Hammamatsu	**Hamamatsu Nanozoomer 40x**
**Hybridization oven for RNAscope**	ACD Bio	HybridZ
**IPTT-300 system**	BMSD	Wi-fi decoder
**Nutator**	Fisher Scientific	Cat # S06622
**Vibratome**	Leica	Leica VT1000
**Softwares**		
ClearMap 2.0	ClearMAP	https://github.com/ChristophKirst/ClearMap2
ImageJ	NIH	https://imagej.nih.gov/ij/; RRID:SCR_003070
TrailMAP	Trailmap	https://github.com/AlbertPun/TRAILMAP
EthoVision XT 7	Noldus	https://www.noldus.com/ethovision; RRID:SCR_000441
Microsoft Excel	microsoft office home edition	N/A
Prism 8	Graphpad	http://www.graphpad.com/; RRID: SCR_002798
Salmon v0.8.2	Salmon	https://bioweb.pasteur.fr/packages/pack@salmon@0.8.2
Zen 2.31	Zeiss	https://www.zeiss.com/microscopy/en/products/software/zeiss-zen.html
TSE Pheomaster Software	TSE Systems	https://www.tse-systems.com/service/phenomaster/
DSeq2 v_1.20.0	R package	https://bioconductor.org/packages/release/bioc/html/DESeq2.html
Imaris 3D software (version 9.1)	Oxford Instruments	http://www.bitplane.com/imaris/imaris; Imaris, RRID:SCR_007370
Tximport version 1.8.0	R package	https://bioconductor.org/packages/release/bioc/html/tximport.html
Rsubread subjunct version 1,30,6	Subread	https://subread.sourceforge.net
Rrtracklayer version 1.40.6	Anaconda	https://anaconda.org/bioconda/bioconductor-rtracklayer/files?version=1.40.6
GSVA version 1.34.0 R package	R package	https://bioconductor.org/packages/release/bioc/html/GSVA.html
Pheatmap R package	R package	https://cran.r-project.org/web/packages/pheatmap/index.html
metap R package	R package	https://cran.r-project.org/web/packages/metap/index.html
complex Heatmap Bioconductor R package	R package	https://bioconductor.org/packages/release/bioc/html/ComplexHeatmap.html
Win non-lin software for LC/MS	Certara	https://www.certara.com/software/phoenix-winnonlin/

Supplementary Table 1. Scored Projection Targets of DRN^{Vglut3} Neurons Identified by Viral-Mediated Tracing and Whole-Brain Clearing.
Quantification of ascending projections from DRN^{Vglut3} neurons from the whole brain projection mapping study using the clearing/immunolabelling technology IDISCO+. No labelling (-), projection targets evidence (+), moderate projection target evidence (++), extensive projection target evidence (+++). Allen brain atlas annotation is used to determine the different regions with ascending projections and both region and acronym is detailed in the table.

Supplementary Table 2. Targets enriched in DRN^{Vglut3} neurons compared to the GENSAT database.
Table showing receptor targets enriched in DRN^{Vglut3} neurons compared to the GENSAT database with a p-value < 0.05. P value is calculated as a two-tailed Wald test. Chi-squared analysis was performed to define the enrichement of DRN^{Vglut3} targets in comparison to the targets expressed by different subsets of neurons in the GENSAT database. Second column shows the fold change in a logarithmic scale. Total of 4094 targets are shown.

Supplementary Table 3. Targets enriched in DRN^{Vglut3} neurons compared to the GENSAT database filtering by plasma membrane receptors and signaling receptors. Table showing receptor targets enriched in DRN^{Vglut3} neurons compared to the GENSAT database and filtering through two Gene Ontology pathways for plasma membrane and signaling receptors with a p-value < 0.05. P value is calculated as a two-tailed Wald test. Chi-squared analysis was performed to define the enrichement of DRN^{Vglut3} targets in comparison to the targets expressed by different subsets of neurons in the GENSAT database and the two enrichment GO paths. Second column shows the fold change in a logarithmic scale. Table shows 691 targets of interest for druggable targeting. From those targets 27 are enriched more than 10-fold in DRN neurons than the rest of profiles available in the GENSAT database and will be evaluated in the Allen Brain Atlas In situ hybridization database.

Supplementary Table 4. CEREP selectivity screen.
Table showing selectivity of CVN45502 at 10 um against a panel of 126 receptors, ion channels, transporters and enzymes conducted at Eurofins (www.eurofinsdiscoveryservices.com) using standard assay protocols.

Supplementary Table 5. Reagents.
Table showcasing all the materials used in the manuscript along with information on the company used to purchase them and their catalog numbers or webpages.

Supplementary Video 1. Ascending Projections from DRN^{Vglut3} Neurons in whole-mount after clearing/immunostaining pipeline IDISCO+.

Video of the 3D whole-brain projection map from DRN^{Vglut3} neuron tracing study in which Vglut3-IRES-Cre mice have been injected with an AAV9-DIO-GFP into the DRN. Staining using GFP antibody is initiated 6 weeks after viral injection allowing for sufficient time for proper axonal projections staining.
