# Supplementary material for: Pharmacological targeting of glutamatergic neurons within the brainstem for weight reduction
Source: Nat Metab. 2022 Nov 21;4(11):1495–513. doi: 10.1038/s42255-022-00677-8 (PMC9684079; doi:10.1038/s42255-022-00677-8)
Supplement: Supplementary file 2 — Reporting Summary [file 42255_2022_677_MOESM2_ESM.pdf]

nature portfolio

Corresponding author(s): Jeffrey M. Friedman

Last updated by author(s): 2022/09/29

Reporting Summary

Nature Portfolio wishes to improve the reproducibility of the work that we publish. This form provides structure for consistency and transparency in reporting. For further information on Nature Portfolio policies, see our Editorial Policies and the Editorial Policy Checklist.

Statistics

For all statistical analyses, confirm that the following items are present in the figure legend, table legend, main text, or Methods section.

n/a	Confirmed	
☐	☒	The exact sample size (*n*) for each experimental group/condition, given as a discrete number and unit of measurement
☒	☐	A statement on whether measurements were taken from distinct samples or whether the same sample was measured repeatedly
☐	☒	The statistical test(s) used AND whether they are one- or two-sided<br>*Only common tests should be described solely by name; describe more complex techniques in the Methods section.*
☒	☐	A description of all covariates tested
☐	☒	A description of any assumptions or corrections, such as tests of normality and adjustment for multiple comparisons
☐	☒	A full description of the statistical parameters including central tendency (e.g. means) or other basic estimates (e.g. regression coefficient) AND variation (e.g. standard deviation) or associated estimates of uncertainty (e.g. confidence intervals)
☐	☒	For null hypothesis testing, the test statistic (e.g. *F*, *t*, *r*) with confidence intervals, effect sizes, degrees of freedom and *P* value noted<br>*Give P values as exact values whenever suitable.*
☒	☐	For Bayesian analysis, information on the choice of priors and Markov chain Monte Carlo settings
☒	☐	For hierarchical and complex designs, identification of the appropriate level for tests and full reporting of outcomes
☒	☐	Estimates of effect sizes (e.g. Cohen's *d*, Pearson's *r*), indicating how they were calculated

Our web collection on statistics for biologists contains articles on many of the points above.

Software and code

Policy information about availability of computer code

Data collection	Zen 2.31 was used for confocal imaging, Inspector Pro (version 4.0.207) for light sheet imaging, Ethovision XT for open field measurements and TSE phenomaster suite for indirect calorimetry. R packages were used for molecular profiling GENSAT comparisons.
Data analysis	GraphPAD prism 8.0.2 (statistics/graphics) was used for all statistic measurements besides indirect calorimetry which used CalR and Molecular Profiling that used R.<br><br>Image J/FIJI v2.0.0.0-rc-69/1.52p was used for ISH and IHC cell counting and image reconstruction.<br><br>Imaris 9.1 was used for light sheet imaging representation and video generation.<br><br>Trailmap and Clearmap 2.0 was used for light sheet projection mapping quantifications. Code for Clearmap 2.0 can be found at https://github.com/CristophKirst/ClearMap2 and for TrailMap at https://github.com/AlbertPun/TRAILMAP. Code to account for imaging corrections specific to our data set are located in supplementary information and can be run in ClearMap2.0 as TubeMapTrailMAp.py<br><br>R packages DEseq2 v1.20.0, Salmon v0.8.2, Tximport version 1.8.0, Rsubread subjunct version 1.30.6, Rrtracklayer version 1.40.6, GSVA version 1.34.0, Pheatmap, Metap and complex Heatmap Bioconductor packages were used for molecular profiling characterization and comparison to GENSAT database. Code availability for the specific runs can be found at https://github.com/RockefellerUniversity/SchneebergerPane_NatureMetabolism2022.

For manuscripts utilizing custom algorithms or software that are central to the research but not yet described in published literature, software must be made available to editors and reviewers. We strongly encourage code deposition in a community repository (e.g. GitHub). See the Nature Portfolio guidelines for submitting code & software for further information.

Data

Policy information about availability of data

All manuscripts must include a data availability statement. This statement should provide the following information, where applicable:

- Accession codes, unique identifiers, or web links for publicly available datasets
- A description of any restrictions on data availability
- For clinical datasets or third party data, please ensure that the statement adheres to our policy

RNAseq data from the vTRAP experiment in DRNvglut3 neurons is publically available following the GSE87890 published in Nectow AR et al. 2017 Cell.
Allen Brain Atlas ISH Database is used for validation of ISH studies conducted with RNAscope technology and is publicly available.

Human research participants

Policy information about studies involving human research participants and Sex and Gender in Research.

Reporting on sex and gender	For ISH studies in human tissue 3 samples from frozen sections of non-diseased donors were used. In particular we used samples from a 59 year old male, from a 58 year old female and from a 78 year old female.
Population characteristics	Population selected was from healthy donors since the sole purpose of the experiments was to confirm the expression of the same target (Hcrtr1) in the same neurons Slc17A8 in humans.
Recruitment	Cerevance acquired the samples with full consent from the Tissues for Research biobank located in the UK and from the General Section of the Douglas-Bell Canada Brain Biobank.
Ethics oversight	Cerevance acquired samples already for more than 11000 donors at multiple Biobanks, all with appropriate ethical consents.

Note that full information on the approval of the study protocol must also be provided in the manuscript.

Field-specific reporting

Please select the one below that is the best fit for your research. If you are not sure, read the appropriate sections before making your selection.

☒ Life sciences ☐ Behavioural & social sciences ☐ Ecological, evolutionary & environmental sciences

For a reference copy of the document with all sections, see nature.com/documents/nr-reporting-summary-flat.pdf

Life sciences study design

All studies must disclose on these points even when the disclosure is negative.

Sample size	No statistical methods were used to calculate the sample size ahead of each study.<br>Sample size was determined based on prior studies and literature in the field as well as animal availability in transgenic breedings to meet age matchings. PMID: 2409368, PMID: 21209617, PMID: 22801496, PMID: 27616062
Data exclusions	No data were excluded
Replication	All data were successfully replicated and data from multiple experiments were pooled in acute experiments. These information is expanded in Statistics and Reproducibility section in the manuscript.<br>Chronic experiments in HFD were performed in large sample sizes and were only performed once given the amount of available drugs and for timing consideration, multiple experiments coming to similar biological conclusions or using different paradigms strengthen the conclusions.
Randomization	For all experiments mice were randomly assigned assuring that mice were age, sex and weight matched prior to weight loss assessments.
Blinding	Data were collected bind, and posthoc registered to the treatment conditions and analyzed to prevent bias.

Reporting for specific materials, systems and methods

We require information from authors about some types of materials, experimental systems and methods used in many studies. Here, indicate whether each material, system or method listed is relevant to your study. If you are not sure if a list item applies to your research, read the appropriate section before selecting a response.

Materials & experimental systems

n/a	Involved in the study	
	x	Antibodies
	x	Eukaryotic cell lines
x		Palaeontology and archaeology
	x	Animals and other organisms
x		Clinical data
x		Dual use research of concern

Methods

n/a	Involved in the study	
x		ChIP-seq
x		Flow cytometry
x		MRI-based neuroimaging

Antibodies

Antibodies used

The following primary antibodies were used, and unless otherwise indicated concentrations apply to all staining techniques. These reagents and others can be found now as Supplementary Table 5. We have used antibodies across multiple lots, and found the results to be comparable across animals and tissues:

Anti-Hcrtr1-(human)- PA5-33838 (1:100) Thermofisher Catalog # PA5-33838; RRID: AB_2551207

Alexa Fluor 488 AffiPure Donkey Anti-Mouse IgG (H+L) (1:1000) Thermofisher Catalog # A-21202; RRID: AB_141607

Alexa Fluor 647 AffiPure Donkey Anti-Rabbitt IgG (H+L) (1:1000) Thermofisher Catalog # A-31573; RRID: AB_2536183

Alexa Fluor 488 AffiPure Donkey Anti-Rabbitt IgG (H+L) (1:1000) Thermofisher Catalog # A-21206; RRID: AB_2535792

Chicken-anti-GFP (1:2000) AVES-LABS Catalog # GFP-1020; RRID: AB_2307313

Anti-GFAP rabbit polyclonal antibody (1:5000) Abcam Catalog # AB7260

Anti-RFP (RABBIT) Antibody Min X Hu Ms and Rt Serum Proteins (1:2000) ROCKLAND Catalog # 600-401-379

RNA-scope technologies (ACD-BIO) in situ probes:

Slc32a1-C3 mouse Acdbio Catalog # 319191

Slc17a6-C3 mouse Acdbio Catalog # 319171

Slc17a8-C2-human Acdbio Catalog # 487431

Hcrtr1-C1-human Acdbio Catalog # 312588

Slc17a8-C3 mouse Acdbio Catalog # 431261

CalcR-C2 mouse Acdbio Catalog # 317518

Hcrtr1-C2 mouse Acdbio Catalog # 466638

Gpcr4-C2 mouse Acdbio Catalog # 427948

Validation

Antibodies used are commercially available and have been thoroughly validated in the literature and by the manufacturer as it can be seen in their webpage.

Human antibody against Hcrtr1 was additionally validated in HEK Cells expressing Hcrtr1 or not (see Extended Data Fig 10).

RFP (PMIDs: 31257028, 33854415)
GFP (PMIDs 31372393, 31257028)
GFAP (PMIDs: 33034847, 33094475)
Alexa 488 donkey anti-Rabbit (PMID: 36130959, 36134661)
Alexa 647 donkey anti-Rabbit (PMID: 36163184, 36151203)
ALexa 488 donkey anti-Mouse (PMID: 35259845, 35240875)

RNAscope probes are all validated by Acdbio before being send and are computationally matched guaranteeing a 100% success..

Eukaryotic cell lines

Policy information about cell lines and Sex and Gender in Research

Cell line source(s)	HEK293FT (Invitrogen R70007), Formalin-Fixed Paraffin-Embedded cell line was obtained from AMSBIO. Data using these lines is presented in Extended Data Figure 10
Authentication	HEK 293FT was purchased by Cerevance from Invitrogen. FFPE line was purchased by Cerevance from AMSBIO. Cells were not authenticated by us.
Mycoplasma contamination	They were all negative from mycoplasma contamination.
Commonly misidentified lines (See ICLAC register)	No commonly misidentified lines.

Animals and other research organisms

Policy information about studies involving animals; ARRIVE guidelines recommended for reporting animal research, and Sex and Gender in Research

Laboratory animals	Adult male and female mice from strains: C57Bl6 (wild type #000664, The Jackson Laboratory), ob/ob (#000632, The Jackson Laboratory) Vglut3-IRES-Cre (Lou et al. 2013)-gift, Vglut2-IRES-Cre (#016963, The Jackson Laboratory). All animals were obtained at 5 weeks of age and HFD started at week 6 until week 22 of age to ensure DIO. Experiments not dependent on DIO were conducted between 12-14 weeks of age.
Wild animals	The study did not include wild animals
Reporting on sex	Most studies were conducted in male mice after basic responses on vglut3 neurons in body weight loss were confirmed for both sexes to minimize animal use.
Field-collected samples	this study did not involve field study samples
Ethics oversight	Rockefeller University IACUC

Note that full information on the approval of the study protocol must also be provided in the manuscript.
